# Supplementary material for: Generation Scotland: an update on Scotland’s longitudinal family health study
Source: BMJ Open. 2024 Jun 21;14(6):e084719. doi: 10.1136/bmjopen-2024-084719 (PMC11340249; doi:10.1136/bmjopen-2024-084719)
Supplement: Supplementary data [file bmjopen-2024-084719supp001.pdf]

Supplementary Material

Appendix A. List of current GS management and delivery team, investigators and members of the Scientific Steering Committee.

**GS Scientific Steering Committee**
Professor Dame Anna F Dominiczak (University of Glasgow & Chief Scientist Office, Scotland) – Chair<br>Professor Cathie Sudlow (University of Edinburgh) – GS Director<br>Professor Heather Whalley (University of Edinburgh) – GS Chief Scientist<br>Professor Julie Brittenden (University of Glasgow)<br>Dr Christian Cole (University of Dundee)<br>Professor Riccardo Marioni (University of Edinburgh)<br>Professor Zosia Miedzybrodzka (University of Aberdeen)<br>Professor Sandosh Padmanabhan (University of Glasgow)<br>Professor Blair Smith (University of Dundee)<br>Katie Wilde (University of Aberdeen)
**GS Scientific Leadership Team**
Professor Cathie Sudlow – GS Director and Principal Investigator (PI)<br>Professor David Porteous - Co-Investigator (Former Director and PI)<br>Professor Andrew McIntosh- Co-Investigator<br>Professor Riccardo Marioni- Co-Investigator<br>Professor Caroline Hayward- Co-Investigator<br>Professor Heather Whalley – GS Chief Scientist and Co-Investigator
**GS Management and Delivery Team**
Professor Cathie Sudlow (University of Edinburgh) – Director<br>Archie Campbell (University of Edinburgh) - Chief Technology Officer<br>Robin Flaig (University of Edinburgh) - Chief Operations Officer<br>Dr Daniel McCartney (University of Edinburgh) - Senior Bioinformatics Analyst<br>Professor Heather Whalley (University of Edinburgh) - Chief Scientist
Fiona Clark (University of Edinburgh) – Partnership Coordinator<br>Elly Darrah (University of Edinburgh) – Administrator<br>Liz Kirby (University of Edinburgh) - Research Support Officer<br>Hannah Milbourn (University of Edinburgh) - Health Data Scientist<br>Anne Richmond (University of Edinburgh) - Bioinformatics Analyst<br>Sarah Robertson (University of Edinburgh) - Young Person Engagement & Recruitment Co-ordinator<br>Rosie Tatham (University of Edinburgh) – Administrator<br>Alex Wood (University of Edinburgh) - Data Analyst / Developer<br>Dr Zhuoni Xiao (University of Edinburgh) – Research Fellow<br>Leah Young (University of Edinburgh) - Research Support Officer

Appendix B. Full list of available linked NHS datasets and descriptions.

[a] Scottish National healthcare data is collected as a series of Scottish Morbidity Records (SMR).

Linked Dataset	Coding	Date Range	N	Description
**Scottish Morbidity Records (SMR)**				
Outpatient Attendance (SMR00)[a]	None	1996-2020	21,270	Outpatient attendance information excluding A&E and genitourinary medicine.
General/Acute Inpatient and Day Case (SMR01)[a]	ICD9/ICD10	1980-2022	19,611	Acute inpatient and daycase hospital admissions: disease and procedure codes. Scotland-wide, around 750 000 admissions per annum since 1981.
Maternity Inpatient and Day Case (SMR02)[a]	ICD9/ICD10	1975-2020	8,238	Pregnancies and births: disease and procedure codes. Scotland-wide, around 58,000 deliveries per annum since 1975.
Mental Health Inpatient and Day Case (SMR04)[a]	ICD9/ICD10	1980-2020	578	Psychiatric hospital discharges and diagnostic information.
Scottish Cancer Registry (SMR06)[a]	ICD10	1996-2020	3,606	Personal, demographic, and diagnostic information on all new cases of cancer.
Scottish Birth Record (SMR11)[a]	ICD9/ICD10	1975-1992	3,246	Linked maternity, neonatal and stillbirth and infant death records from 1975, with records pertaining to mother and baby held together.
**Primary Care**				
General Practice (GP)	ReadV2	1980-2020	19,675	GP primary care attendances.
GP Out-of-Hours Data	ReadV2	2014-2022	8,533	Data on patients seen by GP Out of Hours (OOH) services across Scotland since 2014.
NHS24	Outcome Codes	2011-2022	12,326	Records of telehealth and telecare services provided through nhs24.scot website and NHS 24 111 phone service.
Accident and Emergency (A&E)	ICD10	2007-2020	15,248	Patient attendance information at Emergency Department (EDs), Minor Injuries Units (MIUs) and community hospital A&Es across NHS Scotland.
**Other Datasets**				

Routine laboratory testing	ReadV2	1997-2018	19,089	Laboratory test results from primary and secondary care provided by the Scottish Care Information (SCI) Store.
ICU Daily Data (SICSAG)	None	2019-2022	361	Scottish Intensive Care Society Audit Group (SICSAG) national database of patients admitted to adult Critical Care Units in Scotland since 1995.
ICU Episode Data (SICSAG)	None	2019-2022	361	
National Records of Scotland (NRS) Deaths Data	ICD10	2007-2022	1,659	All deaths occurring in Scotland. NRS Death Records are linked with NHS Scotland Scottish Morbidity Database.
Diabetes Registry (SCI-DC)	None	2019	1,423	The Scottish Care Information – Diabetes Collaboration (SCI-DC), established in 2002. Integrated electronic patient record for individuals with diabetes.
Pre-2009 Dispensing Data (PIS)	BNF	1989-2009	12,981	The Prescribing Information System (PIS) covers all NHS prescriptions prescribed, dispensed and reimbursed within the community setting. Pre-2009 data is poorly completed.
Post-2009 Dispensing Data (PIS)	BNF	2009-2020	21,486	
COVID-19 Vaccinations	None	2020-2022	19,128	Scottish Covid-19 vaccination data contains COVID-19 vaccination events since December 2020.
Testing (ECOSS)	None	2020-2022	16,537	Electronic Communication of Surveillance in Scotland (ECOSS) system surveillance data on COVID-19 infections reported from diagnostics and reference laboratories.
Dental (MIDAS)	SDR	1994-2019	19,870	Management Information & Dental Accounting System (MIDAS), the payment system for GDS dentists, information on NHS dentist appointments.
Scottish Drug Misuse Database	None	2001-2016	76	Scottish Drug Misuse Database (SDMD), attendances at broad range of services.

Appendix C. Secondary Care ICD-10 codes used to investigate cohort morbidities. Codes were derived from Gadd et al [43] using CALIBER code lists. ICD-10 codes are used for all records after 1994 when they replaced the use of ICD-9 codes. Codes were used to query SMR01 (General/Acute Inpatient and Day Case), SMR06 (Scottish Cancer Registry) and NRS death registration datasets.

**Secondary Care ICD-10 Codes**	
**Code**	**Description**
**Asthma**	
J45	Asthma
J46	Status asthmaticus
**Bowel cancer**	
C18	Malignant neoplasm of colon
**Breast cancer**	
C50	Malignant neoplasm of breast
**Chronic obstructive pulmonary disease**	
J40	Bronchitis, not specified as acute or chronic
J41	Simple and mucopurulent chronic bronchitis
J42	Unspecified chronic bronchitis
J43	Emphysema
J44	Other chronic obstructive pulmonary disease
**Dementia**	
F00	Dementia in Alzheimer's disease
F01	Vascular dementia
F03	Unspecified dementia
F051	Delirium superimposed on dementia
G30	Alzheimer's disease
**Depression**	
F32	Major depressive disorder
F33	Major depressive disorder
**Diabetes**	
E10	Type 1 diabetes mellitus
E11	Type 2 diabetes mellitus
E12	Malnutrition-related diabetes mellitus
E13	Other specified diabetes mellitus
E14	Unspecified diabetes mellitus
H360	Diabetic retinopathy
O243	Diabetes mellitus in pregnancy: Pre-existing diabetes mellitus, unspecified
N083	Glomerular disorders in diabetes mellitus
H280	Diabetic cataract
O242	Diabetes mellitus in pregnancy: Pre-existing malnutrition-related diabetes mellitus
M142	Diabetic arthropathy
G632	Diabetic polyneuropathy
O240	Diabetes mellitus in pregnancy: Pre-existing diabetes mellitus, insulin-dependent
O241	Diabetes mellitus in pregnancy: Pre-existing diabetes mellitus, non-insulin-dependent
G590	Diabetic mononeuropathy
**Hypertension**	
I10	Essential (primary) hypertension

I11	Hypertensive heart disease
I12	Hypertensive renal disease
I13	Hypertensive heart and renal disease
I15	Secondary hypertension
**Ischaemic heart disease**	
I20	Angina pectoris
I21	Acute myocardial infarction
I22	Subsequent myocardial infarction
I23	Certain current complications following acute myocardial infarction
I24	Other acute ischaemic heart diseases
I25	Chronic ischaemic heart disease
**Lung Cancer**	
C34	Malignant neoplasm of bronchus and lung
**Osteoarthritis**	
M15	Polyarthrosis
M16	Coxarthrosis [arthrosis of hip]
M17	Gonarthrosis [arthrosis of knee]
M18	Arthrosis of first carpometacarpal joint
M19	Other arthrosis
**Prostate cancer**	
C61	Malignant neoplasm of prostate
**Rheumatoid arthritis**	
M05	Seropositive rheumatoid arthritis
M06	Other rheumatoid arthritis
J99.0	Rheumatoid lung disease
**Stroke**	
I63	Cerebral infarction
I64	Stroke, not specified as haemorrhage or infarction
G46.7	Other lacunar syndromes
G46.6	Pure sensory lacunar syndrome
G46.5	Pure motor lacunar syndrome
G46.3	Brain stem stroke syndrome
G46.4	Cerebellar stroke syndrome
I69.4	Sequelae of stroke, not specified as haemorrhage or infarction
G46.8	Other vascular syndromes of brain in cerebrovascular diseases
**COVID-19**	
U071	COVID-19, virus identified

Appendix D. Secondary Care ICD-9 Codes used to investigate cohort morbidities. ICD-9 codes were used for records up to 1994 when they were replaced with ICD-10. Codes were used to query SMR01 (General/Acute Inpatient and Day Case), SMR06 (Scottish Cancer Registry) and NRS death records datasets.

**Secondary Care ICD-9 Codes**	
**Code**	**Description**
**Asthma**	
493	Asthma
**Bowel cancer**	
153	Malignant neoplasm of colon
154	Malignant neoplasm of rectum rectosigmoid junction and anus
**Breast cancer**	
174	Malignant neoplasm of female breast
**chronic obstructive pulmonary disease**	
490	Bronchitis, not specified as acute or chronic
491	Chronic bronchitis
492	Emphysema
496	Chronic airway obstruction, not elsewhere classified
**Dementia**	
290	Dementias
2912	Alcohol-induced persisting dementia
2941	Dementia in conditions classified elsewhere
331	Other cerebral degenerations
**Depression**	
2962	Major depressive disorder single episode
2963	Major depressive disorder recurrent episode
311	Depressive disorder not elsewhere classified
**Diabetes**	
250	Diabetes mellitus
249	Secondary diabetes mellitus
**Hypertension**	
401	Essential hypertension
402	Hypertensive heart disease
403	Hypertensive renal disease
404	Hypertensive heart and renal disease
405	Secondary hypertension
**Ischaemic heart disease**	
410	Acute myocardial infarction
411	Other acute and subacute forms of ischemic heart disease
412	Old myocardial infarction
413	Angina pectoris
414	Other forms of chronic ischemic heart disease
**Lung cancer**	
162	Malignant neoplasm of trachea bronchus and lung
**Osteoarthritis**	
715	Osteoarthrosis and allied disorders
**Prostate cancer**	

185	Malignant neoplasm of prostate
**Rheumatoid arthritis**	
714	Rheumatoid arthritis and other inflammatory polyarthropathies
**Stroke**	
430	Subarachnoid haemorrhage
431	Intracerebral haemorrhage
433	Occlusion and stenosis of precerebral arteries
434	Occlusion of cerebral arteries

Appendix E. Primary Care ReadCode v2 Codes used to investigate cohort morbidities. Codes were derived from Gadd et al [43] **using CALIBER code lists.**

**Primary Care ReadCode v2 Codes**	
**Code**	**Description**
**Asthma**	
14B4.	H/O: asthma
173A.	Exercise induced asthma
173c.	Occupational asthma
173d.	Work aggravated asthma
1780	Aspirin induced asthma
1O2..	Asthma confirmed
21262	Asthma resolved
212G.	Asthma resolved
H3120	Chronic asthmatic bronchitis
H33..	Asthma
H330.	Extrinsic (atopic) asthma
H3300	Extrinsic asthma without status asthmaticus
H3301	Extrinsic asthma with status asthmaticus
H330z	Extrinsic asthma NOS
H331.	Intrinsic asthma
H3310	Intrinsic asthma without status asthmaticus
H3311	Intrinsic asthma with status asthmaticus
H331z	Intrinsic asthma NOS
H332.	Mixed asthma
H333.	Acute exacerbation of asthma
H334.	Brittle asthma
H335.	Chronic asthma with fixed airflow obstruction
H33z.	Asthma unspecified
H33z0	Status asthmaticus NOS
H33z1	Asthma attack
H33z2	Late-onset asthma
H33zz	Asthma NOS
**Bowel cancer**	
B13..	Malignant neoplasm of colon
B130.	Malignant neoplasm of hepatic flexure of colon
B131.	Malignant neoplasm of transverse colon
B132.	Malignant neoplasm of descending colon
B133.	Malignant neoplasm of sigmoid colon
B134.	Malignant neoplasm of caecum
B135.	Malignant neoplasm of appendix
B136.	Malignant neoplasm of ascending colon
B137.	Malignant neoplasm of splenic flexure of colon
B139.	Hereditary nonpolyposis colon cancer
B13z.	Malignant neoplasm of colon NOS
B1z0.	Malignant neoplasm of intestinal tract, part unspecified
**Breast cancer**	
B3251	Malignant melanoma of breast

B3351	Malignant neoplasm of skin of chest, excluding breast
B3352	Malignant neoplasm of skin of breast
B34..	Malignant neoplasm of female breast
B3401	Malignant neoplasm of areola of female breast
B34y.	Malignant neoplasm of other site of female breast
B34yz	Malignant neoplasm of other site of female breast NOS
B34z.	Malignant neoplasm of female breast NOS
B35..	Malignant neoplasm of male breast
B58y0	Secondary malignant neoplasm of breast
B830.	Carcinoma in situ of breast
BB94.	[M]Juvenile breast carcinoma
Byu6.	[X]Malignant neoplasm of breast
ZV103	[V]Personal history of malignant neoplasm of breast
**Chronic obstructive pulmonary disease**	
14B3.	History of chronic obstructive pulmonary disease
H3...	Chronic obstructive pulmonary disease
H31..	Chronic bronchitis
H310.	Simple chronic bronchitis
H3100	Chronic catarrhal bronchitis
H310z	Simple chronic bronchitis NOS
H311.	Mucopurulent chronic bronchitis
H3110	Purulent chronic bronchitis
H3111	Fetid chronic bronchitis
H311z	Mucopurulent chronic bronchitis NOS
H312.	Obstructive chronic bronchitis
H3120	Chronic asthmatic bronchitis
H3121	Emphysematous bronchitis
H3122	Acute exacerbation of chronic obstructive airways disease
H3123	Bronchiolitis obliterans
H312z	Obstructive chronic bronchitis NOS
H313.	Mixed simple and mucopurulent chronic bronchitis
H31y.	Other chronic bronchitis
H31y1	Chronic tracheobronchitis
H31yz	Other chronic bronchitis NOS
H31z.	Chronic bronchitis NOS
H32..	Emphysema
H320.	Chronic bullous emphysema
H3200	Segmental bullous emphysema
H3201	Zonal bullous emphysema
H3202	Giant bullous emphysema
H3203	Bullous emphysema with collapse
H320z	Chronic bullous emphysema NOS
H321.	Panlobular emphysema
H322.	Centrilobular emphysema
H32y.	Other emphysema
H32y0	Acute vesicular emphysema
H32y1	Atrophic (senile) emphysema
H32y2	MacLeod's unilateral emphysema

H32yz	Other emphysema NOS
H32z.	Emphysema NOS
H36..	Mild chronic obstructive pulmonary disease
H37..	Moderate chronic obstructive pulmonary disease
H38..	Severe chronic obstructive pulmonary disease
H39..	Very severe chronic obstructive pulmonary disease
H3A..	End stage chronic obstructive airways disease
H3y..	Other specified chronic obstructive airways disease
H3y0.	Chronic obstruct pulmonary dis with acute lower respiratory infection
H3y1.	Chronic obstruct pulmonary dis with acute exacerbation, unspecified
H3z..	Chronic obstructive airways disease NOS
H4640	Chronic emphysema due to chemical fumes
H4641	Obliterative bronchiolitis due to chemical fumes
H5832	Eosinophilic bronchitis
Hyu30	[X]Other emphysema
Hyu31	[X]Other specified chronic obstructive pulmonary disease
**Dementia**	
1461	H/O: dementia
66h..	Dementia monitoring
6AB..	Dementia annual review
8CMZ.	Dementia care plan
9hD..	Exception reporting: dementia quality indicators
9hD0.	Excepted from dementia quality indicators: Patient unsuitable
9hD1.	Excepted from dementia quality indicators: Informed dissent
9Ou..	Dementia monitoring administration
9Ou1.	Dementia monitoring first letter
9Ou2.	Dementia monitoring second letter
9Ou3.	Dementia monitoring third letter
9Ou4.	Dementia monitoring verbal invite
9Ou5.	Dementia monitoring telephone invite
E00..	Senile and presenile organic psychotic conditions
E000.	Uncomplicated senile dementia
E001.	Presenile dementia
E0010	Uncomplicated presenile dementia
E0011	Presenile dementia with delirium
E0012	Presenile dementia with paranoia
E0013	Presenile dementia with depression
E001z	Presenile dementia NOS
E002.	Senile dementia with depressive or paranoid features
E0020	Senile dementia with paranoia
E0021	Senile dementia with depression
E002z	Senile dementia with depressive or paranoid features NOS
E003.	Senile dementia with delirium
E004.	Arteriosclerotic dementia
E0040	Uncomplicated arteriosclerotic dementia
E0041	Arteriosclerotic dementia with delirium
E0042	Arteriosclerotic dementia with paranoia

E0043	Arteriosclerotic dementia with depression
E004z	Arteriosclerotic dementia NOS
E00y.	Other senile and presenile organic psychoses
E00z.	Senile or presenile psychoses NOS
E041.	Dementia in conditions EC
Eu00.	[X]Dementia in Alzheimer's disease
Eu000	[X]Dementia in Alzheimer's disease with early onset
Eu001	[X]Dementia in Alzheimer's disease with late onset
Eu002	[X]Dementia in Alzheimer's dis, atypical or mixed type
Eu00z	[X]Dementia in Alzheimer's disease, unspecified
Eu01.	[X]Vascular dementia
Eu010	[X]Vascular dementia of acute onset
Eu011	[X]Multi-infarct dementia
Eu012	[X]Subcortical vascular dementia
Eu013	[X]Mixed cortical and subcortical vascular dementia
Eu01y	[X]Other vascular dementia
Eu01z	[X]Vascular dementia, unspecified
Eu02z	[X] Unspecified dementia
Eu041	[X]Delirium superimposed on dementia
F110.	Alzheimer's disease
F1100	Alzheimer's disease with early onset
F1101	Alzheimer's disease with late onset
Fyu30	[X]Other Alzheimer's disease
ZS7C5	Language disorder of dementia
**Depression**	
1465	H/O: depression
212S.	Depression resolved
8BK0.	Depression management programme
8CAa.	Patient given advice about management of depression
8HHq.	Referral for guided self-help for depression
9H90.	Depression annual review
9H91.	Depression medication review
9H92.	Depression interim review
9HA0.	On depression register
9k4..	Depression - enhanced services administration
9k40.	Depression - enhanced service completed
9kQ..	On full dose long term treatment depression - enh serv admin
9Ov..	Depression monitoring administration
9Ov0.	Depression monitoring first letter
9Ov1.	Depression monitoring second letter
9Ov2.	Depression monitoring third letter
9Ov3.	Depression monitoring verbal invite
9Ov4.	Depression monitoring telephone invite
E0013	Presenile dementia with depression
E0021	Senile dementia with depression
E0043	Arteriosclerotic dementia with depression
E112.	Single major depressive episode
E1120	Single major depressive episode, unspecified

E1121	Single major depressive episode, mild
E1122	Single major depressive episode, moderate
E1123	Single major depressive episode, severe, without psychosis
E1124	Single major depressive episode, severe, with psychosis
E1125	Single major depressive episode, partial or unspec remission
E1126	Single major depressive episode, in full remission
E112z	Single major depressive episode NOS
E113.	Recurrent major depressive episode
E1130	Recurrent major depressive episodes, unspecified
E1131	Recurrent major depressive episodes, mild
E1132	Recurrent major depressive episodes, moderate
E1133	Recurrent major depressive episodes, severe, no psychosis
E1134	Recurrent major depressive episodes, severe, with psychosis
E1135	Recurrent major depressive episodes,partial/unspec remission
E1136	Recurrent major depressive episodes, in full remission
E1137	Recurrent depression
E113z	Recurrent major depressive episode NOS
E118.	Seasonal affective disorder
E11y2	Atypical depressive disorder
E11z2	Masked depression
E130.	Reactive depressive psychosis
E135.	Agitated depression
E2003	Anxiety with depression
E291.	Prolonged depressive reaction
E2B..	Depressive disorder NEC
E2B1.	Chronic depression
Eu204	[X]Post-schizophrenic depression
Eu251	[X]Schizoaffective disorder, depressive type
Eu32.	[X]Depressive episode
Eu320	[X]Mild depressive episode
Eu321	[X]Moderate depressive episode
Eu322	[X]Severe depressive episode without psychotic symptoms
Eu323	[X]Severe depressive episode with psychotic symptoms
Eu324	[X]Mild depression
Eu325	[X]Major depression, mild
Eu326	[X]Major depression, moderately severe
Eu327	[X]Major depression, severe without psychotic symptoms
Eu328	[X]Major depression, severe with psychotic symptoms
Eu329	[X]Single major depr ep, severe with psych, psych in remiss
Eu32A	[X]Recurr major depr ep, severe with psych, psych in remiss
Eu32y	[X]Other depressive episodes
Eu32z	[X]Depressive episode, unspecified
Eu33.	[X]Recurrent depressive disorder
Eu330	[X]Recurrent depressive disorder, current episode mild
Eu331	[X]Recurrent depressive disorder, current episode moderate
Eu332	[X]Recurr depress disorder cur epi severe without psyc sympt
Eu333	[X]Recurrent depress disorder cur epi severe with psyc symp
Eu334	[X]Recurrent depressive disorder, currently in remission

Eu33y	[X]Other recurrent depressive disorders
Eu33z	[X]Recurrent depressive disorder, unspecified
Eu341	[X]Dysthymia
Eu412	[X]Mixed anxiety and depressive disorder
**Diabetes**	
C108.	Type 1 diabetes mellitus
C1080	Type 1 diabetes mellitus with renal complications
C1082	Type 1 diabetes mellitus with neurological complications
C1084	Unstable type 1 diabetes mellitus
C1085	Type 1 diabetes mellitus with ulcer
C1087	Type 1 diabetes mellitus with retinopathy
C1088	Type 1 diabetes mellitus - poor control
C1089	Type 1 diabetes mellitus maturity onset
C108A	Type 1 diabetes mellitus without complication
C108D	Type 1 diabetes mellitus with nephropathy
C108E	Type 1 diabetes mellitus with hypoglycaemic coma
C108F	Type 1 diabetes mellitus with diabetic cataract
C108H	Type 1 diabetes mellitus with arthropathy
C108J	Type 1 diabetes mellitus with neuropathic arthropathy
C109.	Type 2 diabetes mellitus
C1090	Type 2 diabetes mellitus with renal complications
C1091	Type 2 diabetes mellitus with ophthalmic complications
C1092	Type 2 diabetes mellitus with neurological complications
C1094	Type 2 diabetes mellitus with ulcer
C1095	Type 2 diabetes mellitus with gangrene
C1096	Type 2 diabetes mellitus with retinopathy
C1097	Type 2 diabetes mellitus - poor control
C109A	Type 2 diabetes mellitus with mononeuropathy
C109B	Type 2 diabetes mellitus with polyneuropathy
C109C	Type 2 diabetes mellitus with nephropathy
C109D	Type 2 diabetes mellitus with hypoglycaemic coma
C109E	Type 2 diabetes mellitus with diabetic cataract
C109F	Type 2 diabetes mellitus with peripheral angiopathy
C109G	Type 2 diabetes mellitus with arthropathy
C109H	Type 2 diabetes mellitus with neuropathic arthropathy
C109J	Insulin treated Type 2 diabetes mellitus
C109K	Hyperosmolar non-ketotic state in type 2 diabetes mellitus
C10E.	Type 1 diabetes mellitus
C10E0	Type 1 diabetes mellitus with renal complications
C10E1	Type 1 diabetes mellitus with ophthalmic complications
C10E2	Type 1 diabetes mellitus with neurological complications
C10E3	Type 1 diabetes mellitus with multiple complications
C10E4	Unstable type 1 diabetes mellitus
C10E5	Type 1 diabetes mellitus with ulcer
C10E6	Type 1 diabetes mellitus with gangrene
C10E7	Type 1 diabetes mellitus with retinopathy
C10E8	Type 1 diabetes mellitus - poor control
C10E9	Type 1 diabetes mellitus maturity onset

C10EA	Type 1 diabetes mellitus without complication
C10EB	Type 1 diabetes mellitus with mononeuropathy
C10EC	Type 1 diabetes mellitus with polyneuropathy
C10ED	Type 1 diabetes mellitus with nephropathy
C10EE	Type 1 diabetes mellitus with hypoglycaemic coma
C10EF	Type 1 diabetes mellitus with diabetic cataract
C10EG	Type 1 diabetes mellitus with peripheral angiopathy
C10EH	Type 1 diabetes mellitus with arthropathy
C10EJ	Type 1 diabetes mellitus with neuropathic arthropathy
C10EK	Type 1 diabetes mellitus with persistent proteinuria
C10EL	Type 1 diabetes mellitus with persistent microalbuminuria
C10EM	Type 1 diabetes mellitus with ketoacidosis
C10EN	Type 1 diabetes mellitus with ketoacidotic coma
C10EP	Type 1 diabetes mellitus with exudative maculopathy
C10EQ	Type 1 diabetes mellitus with gastroparesis
C10F.	Type 2 diabetes mellitus
C10F0	Type 2 diabetes mellitus with renal complications
C10F1	Type 2 diabetes mellitus with ophthalmic complications
C10F2	Type 2 diabetes mellitus with neurological complications
C10F3	Type 2 diabetes mellitus with multiple complications
C10F4	Type 2 diabetes mellitus with ulcer
C10F5	Type 2 diabetes mellitus with gangrene
C10F6	Type 2 diabetes mellitus with retinopathy
C10F7	Type 2 diabetes mellitus - poor control
C10F9	Type 2 diabetes mellitus without complication
C10FA	Type 2 diabetes mellitus with mononeuropathy
C10FB	Type 2 diabetes mellitus with polyneuropathy
C10FC	Type 2 diabetes mellitus with nephropathy
C10FD	Type 2 diabetes mellitus with hypoglycaemic coma
C10FE	Type 2 diabetes mellitus with diabetic cataract
C10FF	Type 2 diabetes mellitus with peripheral angiopathy
C10FG	Type 2 diabetes mellitus with arthropathy
C10FH	Type 2 diabetes mellitus with neuropathic arthropathy
C10FJ	Insulin treated Type 2 diabetes mellitus
C10FK	Hyperosmolar non-ketotic state in type 2 diabetes mellitus
C10FL	Type 2 diabetes mellitus with persistent proteinuria
C10FM	Type 2 diabetes mellitus with persistent microalbuminuria
C10FN	Type 2 diabetes mellitus with ketoacidosis
C10FP	Type 2 diabetes mellitus with ketoacidotic coma
C10FQ	Type 2 diabetes mellitus with exudative maculopathy
C10FR	Type 2 diabetes mellitus with gastroparesis
**Hypertension**	
14A2.	H/O: hypertension
21261	Hypertension resolved
212K.	Hypertension resolved
61462	Hypertension induced by oral contraceptive pill
6624	Borderline hypertension: yearly observation
6627	Good hypertension control

6628	Poor hypertension control
662b.	Moderate hypertension control
662c.	Hypertension six month review
662d.	Hypertension annual review
662F.	Hypertension treatment. started
662G.	Hypertensive treatment changed
662O.	On treatment for hypertension
662r.	Trial withdrawal of antihypertensive therapy
7Q01.	High cost hypertension drugs
8B26.	Antihypertensive therapy
8BL0.	Patient on maximal tolerated antihypertensive therapy
8I3N.	Hypertension treatment refused
9OI9.	Hypertension monitor deleted
F4042	Blind hypertensive eye
F4213	Hypertensive retinopathy
G2...	Hypertensive disease
G20..	Essential hypertension
G200.	Malignant essential hypertension
G201.	Benign essential hypertension
G202.	Systolic hypertension
G203.	Diastolic hypertension
G20z.	Essential hypertension NOS
G21..	Hypertensive heart disease
G210.	Malignant hypertensive heart disease
G2100	Malignant hypertensive heart disease without CCF
G2101	Malignant hypertensive heart disease with CCF
G211.	Benign hypertensive heart disease
G2110	Benign hypertensive heart disease without CCF
G2111	Benign hypertensive heart disease with CCF
G21z.	Hypertensive heart disease NOS
G21z0	Hypertensive heart disease NOS without CCF
G21z1	Hypertensive heart disease NOS with CCF
G21zz	Hypertensive heart disease NOS
G22..	Hypertensive renal disease
G220.	Malignant hypertensive renal disease
G221.	Benign hypertensive renal disease
G222.	Hypertensive renal disease with renal failure
G22z.	Hypertensive renal disease NOS
G23..	Hypertensive heart and renal disease
G230.	Malignant hypertensive heart and renal disease
G231.	Benign hypertensive heart and renal disease
G232.	Hypertensive heart&renal dis wth (congestive) heart failure
G233.	Hypertensive heart and renal disease with renal failure
G234.	Hyperten heart&renal dis+both(congestv)heart and renal fail
G23z.	Hypertensive heart and renal disease NOS
G24..	Secondary hypertension
G240.	Secondary malignant hypertension
G2400	Secondary malignant renovascular hypertension

G240z	Secondary malignant hypertension NOS
G241.	Secondary benign hypertension
G2410	Secondary benign renovascular hypertension
G241z	Secondary benign hypertension NOS
G244.	Hypertension secondary to endocrine disorders
G24z.	Secondary hypertension NOS
G24z0	Secondary renovascular hypertension NOS
G24z1	Hypertension secondary to drug
G24zz	Secondary hypertension NOS
G2y..	Other specified hypertensive disease
G2z..	Hypertensive disease NOS
G672.	Hypertensive encephalopathy
Gyu2.	[X]Hypertensive diseases
Gyu21	[X]Hypertension secondary to other renal disorders
L122.	Other pre-existing hypertension in preg/childbirth/puerp
L1220	Other pre-existing hypertension in preg/childb/puerp unspec
L1221	Other pre-existing hypertension in preg/childb/puerp - deliv
L1223	Other pre-exist hypertension in preg/childb/puerp-not deliv
L122z	Other pre-existing hypertension in preg/childb/puerp NOS
L127.	Pre-eclampsia or eclampsia with pre-existing hypertension
L127z	Pre-eclampsia or eclampsia + pre-existing hypertension NOS
L128.	Pre-exist hypertension compl preg childbirth and puerperium
L1280	Pre-exist hyperten heart dis compl preg childbth+puerperium
L1282	Pre-exist 2ndry hypertens comp preg childbth and puerperium
TJC7.	Adverse reaction to other antihypertensives
TJC7z	Adverse reaction to antihypertensives NOS
U60C5	[X]Oth antihyperten drug caus advers eff in therap use, NEC
**Ischaemic heart disease**	
G3...	Ischaemic heart disease
G30..	Acute myocardial infarction
G300.	Acute anterolateral infarction
G301.	Other specified anterior myocardial infarction
G301z	Anterior myocardial infarction NOS
G302.	Acute inferolateral infarction
G303.	Acute inferoposterior infarction
G304.	Posterior myocardial infarction NOS
G305.	Lateral myocardial infarction NOS
G307.	Acute subendocardial infarction
G3070	Acute non-Q wave infarction
G3071	Acute non-ST segment elevation myocardial infarction
G308.	Inferior myocardial infarction NOS
G309.	Acute Q-wave infarct
G30A.	Mural thrombosis
G30X0	Acute ST segment elevation myocardial infarction
G30yz	Other acute myocardial infarction NOS
G30z.	Acute myocardial infarction NOS
G31..	Other acute and subacute ischaemic heart disease
G310.	Postmyocardial infarction syndrome

G311.	Preinfarction syndrome
G3110	Myocardial infarction aborted
G3111	Unstable angina
G3112	Angina at rest
G3114	Worsening angina
G3115	Acute coronary syndrome
G31y0	Acute coronary insufficiency
G31yz	Other acute and subacute ischaemic heart disease NOS
G32..	Old myocardial infarction
G33..	Angina pectoris
G3300	Nocturnal angina
G330z	Angina decubitus NOS
G331.	Prinzmetal's angina
G332.	Coronary artery spasm
G33z.	Angina pectoris NOS
G33z3	Angina on effort
G33z4	Ischaemic chest pain
G33z6	New onset angina
G33z7	Stable angina
G33zz	Angina pectoris NOS
G340.	Coronary atherosclerosis
G3400	Single coronary vessel disease
G3401	Double coronary vessel disease
G341.	Aneurysm of heart
G3410	Ventricular cardiac aneurysm
G342.	Atherosclerotic cardiovascular disease
G343.	Ischaemic cardiomyopathy
G34z.	Other chronic ischaemic heart disease NOS
G34z0	Asymptomatic coronary heart disease
G35..	Subsequent myocardial infarction
G366.	Thrombosis of atrium, auricular appendage, and ventricle as current complications following acute myocardial infarction
G37..	Cardiac syndrome X
G38..	Postoperative myocardial infarction
G39..	Coronary microvascular disease
G3z..	Ischaemic heart disease NOS
**Lung cancer**	
B22..	Malignant neoplasm of trachea, bronchus and lung
B2211	Malignant neoplasm of hilus of lung
B222.	Malignant neoplasm of upper lobe, bronchus or lung
B2221	Malignant neoplasm of upper lobe of lung
B2231	Malignant neoplasm of middle lobe of lung
B224.	Malignant neoplasm of lower lobe, bronchus or lung
B2241	Malignant neoplasm of lower lobe of lung
B22z.	Malignant neoplasm of bronchus or lung NOS
B570.	Secondary malignant neoplasm of lung
**Osteoarthritis**	
14G2.	H/O: osteoarthritis

2G26.	O/E - hands - Heberden's nodes
7P204	Delivery of rehabilitation for osteoarthritis
N05..	Osteoarthritis and allied disorders
N050.	Generalised osteoarthritis - OA
N0500	Generalised osteoarthritis of unspecified site
N0501	Generalised osteoarthritis of the hand
N0502	Generalised osteoarthritis of multiple sites
N0503	Bouchard's nodes with arthropathy
N0504	Primary generalized osteoarthrosis
N0505	Secondary multiple arthrosis
N0506	Erosive osteoarthrosis
N0507	Heberden's nodes with arthropathy
N050z	Generalised osteoarthritis NOS
N051.	Localised, primary osteoarthritis
N0510	Localised, primary osteoarthritis of unspecified site
N0511	Localised, primary osteoarthritis of the shoulder region
N0512	Localised, primary osteoarthritis of the upper arm
N0513	Localised, primary osteoarthritis of the forearm
N0514	Localised, primary osteoarthritis of the hand
N0515	Localised, primary osteoarthritis of the pelvic region/thigh
N0516	Localised, primary osteoarthritis of the lower leg
N0517	Localised, primary osteoarthritis of the ankle and foot
N0518	Localised, primary osteoarthritis of other specified site
N0519	Primary coxarthrosis, bilateral
N051A	Coxarthrosis resulting from dysplasia, bilateral
N051B	Primary gonarthrosis, bilateral
N051C	Primary arthrosis of first carpometacarpal joints, bilateral
N051D	Localised, primary osteoarthritis of the wrist
N051E	Localised, primary osteoarthritis of toe
N051F	Localised, primary osteoarthritis of elbow
N051G	Osteoarthritis of spinal facet joint
N051z	Localised, primary osteoarthritis NOS
N052.	Localised, secondary osteoarthritis
N0520	Localised, secondary osteoarthritis of unspecified site
N0521	Localised, secondary osteoarthritis of the shoulder region
N0522	Localised, secondary osteoarthritis of the upper arm
N0523	Localised, secondary osteoarthritis of the forearm
N0524	Localised, secondary osteoarthritis of the hand
N0525	Localised, secondary osteoarthritis of pelvic region/thigh
N0526	Localised, secondary osteoarthritis of the lower leg
N0527	Localised, secondary osteoarthritis of the ankle and foot
N0528	Localised, secondary osteoarthritis of other specified site
N052z	Localised, secondary osteoarthritis NOS
N053.	Localised osteoarthritis, unspecified
N0530	Localised osteoarthritis, unspecified, of unspecified site
N0531	Localised osteoarthritis, unspecified, of shoulder region
N0532	Localised osteoarthritis, unspecified, of the upper arm
N0533	Localised osteoarthritis, unspecified, of the forearm

N0534	Localised osteoarthritis, unspecified, of the hand
N0535	Localised osteoarthritis, unspecified, pelvic region/thigh
N0536	Localised osteoarthritis, unspecified, of the lower leg
N0537	Localised osteoarthritis, unspecified, of the ankle and foot
N0538	Localised osteoarthritis, unspecified, of other spec site
N0539	Arthrosis of first carpometacarpal joint, unspecified
N053z	Localised osteoarthritis, unspecified, NOS
N054.	Oligoarticular osteoarthritis, unspecified
N0540	Oligoarticular osteoarthritis, unspec, of unspecified sites
N0541	Oligoarticular osteoarthritis, unspecified, of shoulder
N0542	Oligoarticular osteoarthritis, unspecified, of upper arm
N0544	Oligoarticular osteoarthritis, unspecified, of hand
N0545	Oligoarticular osteoarthritis, unspecified, of pelvis/thigh
N0546	Oligoarticular osteoarthritis, unspecified, of lower leg
N0547	Oligoarticular osteoarthritis, unspecified, of ankle/foot
N0548	Oligoarticular osteoarthritis, unspecified, other spec sites
N0549	Oligoarticular osteoarthritis, unspecified, multiple sites
N054z	Osteoarthritis of more than one site, unspecified, NOS
N05z.	Osteoarthritis NOS
N05z0	Osteoarthritis NOS, of unspecified site
N05z1	Osteoarthritis NOS, of shoulder region
N05z4	Osteoarthritis NOS, of the hand
N05z5	Osteoarthritis NOS, pelvic region/thigh
N05z6	Osteoarthritis NOS, of the lower leg
N05z7	Osteoarthritis NOS, of ankle and foot
N05z8	Osteoarthritis NOS, other specified site
N05z9	Osteoarthritis NOS, of shoulder
N05zA	Osteoarthritis NOS, of sternoclavicular joint
N05zB	Osteoarthritis NOS, of acromioclavicular joint
N05zC	Osteoarthritis NOS, of elbow
N05zD	Osteoarthritis NOS, of distal radio-ulnar joint
N05zE	Osteoarthritis NOS, of wrist
N05zF	Osteoarthritis NOS, of MCP joint
N05zG	Osteoarthritis NOS, of PIP joint of finger
N05zH	Osteoarthritis NOS, of DIP joint of finger
N05zJ	Osteoarthritis NOS, of hip
N05zK	Osteoarthritis NOS, of sacro-iliac joint
N05zL	Osteoarthritis NOS, of knee
N05zM	Osteoarthritis NOS, of tibio-fibular joint
N05zN	Osteoarthritis NOS, of ankle
N05zP	Osteoarthritis NOS, of subtalar joint
N05zQ	Osteoarthritis NOS, of talonavicular joint
N05zR	Osteoarthritis NOS, of other tarsal joint
N05zS	Osteoarthritis NOS, of 1st MTP joint
N05zT	Osteoarthritis NOS, of lesser MTP joint
N05zU	Osteoarthritis NOS, of IP joint of toe
N05zz	Osteoarthritis NOS
Nyu2.	[X]Arthrosis

Nyu20	[X]Other polyarthrosis
Nyu21	[X]Other primary coxarthrosis
Nyu22	[X]Other dysplastic coxarthrosis
Nyu24	[X]Other secondary coxarthrosis, bilateral
Nyu25	[X]Other primary gonarthrosis
Nyu27	[X]Other secondary gonarthrosis, bilateral
Nyu28	[X]Other secondary gonarthrosis
Nyu29	[X]Other primary arthrosis of first carpometacarpal joint
Nyu2D	[X]Other specified arthrosis
Nyu2E	[X]Other secondary coxarthrosis
**Prostate cancer**	
7B365	Radical prostatectomy without pelvic node excision
4M01.	Gleason prostate grade 5-7 (medium)
4M02.	Gleason prostate grade 8-10 (high)
4M00.	Gleason prostate grade 2-4 (low)
B46..	Malignant neoplasm of prostate
7B360	Radical prostatectomy - unspecified excision of pelvic nodes
ZV104	[V]Personal history of malignant neoplasm of prostate
7B367	Radical prostatectomy with pelvic lymphadenectomy
4M0..	Gleason grading of prostate cancer
7B200	Radical cystoprostatourethrectomy
7B366	Radical prostatectomy with pelvic node sampling
14270	H/O: prostate cancer
7B202	Radical cystoprostatectomy
**Rheumatoid arthritis**	
14G1.	H/O: rheumatoid arthritis
2G25.	O/E - hands - ulnar deviation
2G27.	O/E-hands-rheumatoid spindling
66H..	Rheumatoid arthrit. monitoring
7P203	Delivery of rehabilitation for rheumatoid arthritis
9mM..	Rheumatoid arthritis monitoring invitation
9mM0.	Rheumatoid arthritis monitoring invitation first letter
9mM1.	Rheumatoid arthritis monitoring invitation second letter
9mM2.	Rheumatoid arthritis monitoring invitation third letter
9mM3.	Rheumatoid arthritis monitoring verbal invitation
9mM4.	Rheumatoid arthritis monitoring telephone invitation
F3712	Polyneuropathy in rheumatoid arthritis
F3964	Myopathy due to rheumatoid arthritis
G5y8.	Rheumatoid myocarditis
G5yA.	Rheumatoid carditis
H570.	Rheumatoid lung
N005.	Adult Still's Disease
N04..	Rheumatoid arthritis and other inflammatory polyarthropathy
N040.	Rheumatoid arthritis
N0400	Rheumatoid arthritis of cervical spine
N0401	Other rheumatoid arthritis of spine
N0402	Rheumatoid arthritis of shoulder
N0403	Rheumatoid arthritis of sternoclavicular joint

N0404	Rheumatoid arthritis of acromioclavicular joint
N0405	Rheumatoid arthritis of elbow
N0406	Rheumatoid arthritis of distal radio-ulnar joint
N0407	Rheumatoid arthritis of wrist
N0408	Rheumatoid arthritis of MCP joint
N0409	Rheumatoid arthritis of PIP joint of finger
N040A	Rheumatoid arthritis of DIP joint of finger
N040B	Rheumatoid arthritis of hip
N040C	Rheumatoid arthritis of sacro-iliac joint
N040D	Rheumatoid arthritis of knee
N040E	Rheumatoid arthritis of tibio-fibular joint
N040F	Rheumatoid arthritis of ankle
N040G	Rheumatoid arthritis of subtalar joint
N040H	Rheumatoid arthritis of talonavicular joint
N040J	Rheumatoid arthritis of other tarsal joint
N040K	Rheumatoid arthritis of 1st MTP joint
N040L	Rheumatoid arthritis of lesser MTP joint
N040M	Rheumatoid arthritis of IP joint of toe
N040N	Rheumatoid vasculitis
N040P	Seronegative rheumatoid arthritis
N040Q	Rheumatoid bursitis
N040R	Rheumatoid nodule
N040S	Rheumatoid arthritis - multiple joint
N040T	Flare of rheumatoid arthritis
N041.	Felty's syndrome
N042.	Other rheumatoid arthropathy + visceral/systemic involvement
N0421	Rheumatoid lung disease
N0422	Rheumatoid nodule
N042z	Rheumatoid arthropathy + visceral/systemic involvement NOS
N047.	Seropositive errosive rheumatoid arthritis
N04X.	Seropositive rheumatoid arthritis
N04y0	Rheumatoid lung
N04y2	Adult-onset Still's disease
N3622	Swan-neck finger deformity
Nyu10	[X]Rheumatoid arthritis+involvement/other organs or systems
Nyu11	[X]Other seropositive rheumatoid arthritis
Nyu12	[X]Other specified rheumatoid arthritis
Nyu1G	[X]Seropositive rheumatoid arthritis unspecified
**Stroke**	
G6400	Cerebral infarction due to thrombosis of cerebral arteries
G6W..	Cereb infarct due unsp occlus/stenos precerebr arteries
Gyu63	[X]Cerebrl infarctn due/unspcf occlusn or sten/cerebrl artrs
G63y0	Cerebral infarct due to thrombosis of precerebral arteries
G683.	Sequelae of cerebral infarction
G64z3	Right sided cerebral infarction
Gyu6G	[X]Cereb infarct due unsp occlus/stenos precerebr arteries
G640.	Cerebral thrombosis
G64z.	Cerebral infarction NOS

G641.	Cerebral embolism
G63..	Infarction - precerebral
G64z0	Brainstem infarction
G6410	Cerebral infarction due to embolism of cerebral arteries
G64z4	Infarction of basal ganglia
G6X..	Cerebrl infarctn due/unspcf occlusn or sten/cerebrl artrs
G64z1	Wallenberg syndrome
G64..	Cerebral arterial occlusion
G63y1	Cerebral infarction due to embolism of precerebral arteries
G64z2	Left sided cerebral infarction
Gyu64	[X]Other cerebral infarction
14A7.	H/O: CVA/stroke
14AK.	H/O: Stroke in last year
1M4..	Central post-stroke pain
661M7	Stroke self-management plan agreed
661N7	Stroke self-management plan review
662e.	Stroke/CVA annual review
662M.	Stroke monitoring
662M1	Stroke 6 month review
662M2	Stroke initial post discharge review
7P242	Delivery of rehabilitation for stroke
8HHM.	Ref to multidisciplinary stroke function improvement service
8IEC.	Ref multidisciplinary stroke function improvement declined
9h2..	Exception reporting: stroke quality indicators
9h21.	Excepted from stroke quality indicators: Patient unsuitable
9h22.	Excepted from stroke quality indicators: Informed dissent
Fyu56	[X]Other lacunar syndromes
G66..	Stroke and cerebrovascular accident unspecified
G663.	Brain stem stroke syndrome
G664.	Cerebellar stroke syndrome
G665.	Pure motor lacunar syndrome
G666.	Pure sensory lacunar syndrome
G667.	Left sided CVA
G668.	Right sided CVA
G68X.	Sequelae of stroke, not specified as haemorrhage or infarction
Gyu6C	[X]Sequelae of stroke, not specified as haemorrhage or infarction
L440.	Stroke in the puerperium
ZV125	[V]Personal history of stroke
COVID-19	
1JX1.	Suspected disease caused by 2019-nCoV (novel coronavirus)
A7951	Disease caused by 2019-nCoV (novel coronavirus)
4J3R.	2019-nCoV (novel coronavirus) serology
4J3R1	2019-nCoV (novel coronavirus) detected

Appendix F. Summary of the top 50 most cited publications using GS data. A full list of publications can be found at https://www.ed.ac.uk/generation-scotland/what-found/publications.

First Author	Title	Year Published	Journal	Keywords	Citations
Wray	Genome-wide association analyses identify 44 risk variants and refine the genetic architecture of major depression.	2018	Nature Genetics	Cognition and Mental Health	2,243
Lee	Gene discovery and polygenic prediction from a genome-wide association study of educational attainment in 1.1 million individuals.	2018	Nature Genetics	Data	1,714
Howard	Genome-wide meta-analysis of depression identifies 102 independent variants and highlights the importance of the prefrontal brain regions.	2019	Nature Neuroscience	Cognition and Mental Health	1,483
Pardiñas	Common schizophrenia alleles are enriched in mutation-intolerant genes and in regions under strong background selection.	2018	Nature Genetics	Cognition and Mental Health	1,376
Okbay	Genome-wide association study identifies 74 loci associated with educational attainment.	2016	Nature	Cognition and Mental Health	1,292
Anttila	Analysis of shared heritability in common disorders of the brain.	2018	Science	Cognition and Mental Health	1,202
Pairo-Castineira	Genetic mechanisms of critical illness in Covid-19.	2020	Nature	COVID-19	1,162
UK10K Consortium	The UK10K project identifies rare variants in health and disease.	2015	Nature	Data	1,030
Rahbari	Timing, rates and spectra of human germline mutation.	2015	Nature Genetics	Data	859
Lazaridis	Genomic insights into the origin of farming in the ancient Near East.	2016	Nature	Data	850
Fitzgerald	Large-scale discovery of novel genetic causes of developmental disorders.	2014	Nature	Cognition and Mental Health	693
Repapi	Genome-wide association study identifies five loci associated with lung function.	2009	Nature Genetics	Respiratory Disease	646
Davies	Study of 300,486 individuals identifies 148 independent genetic loci influencing general cognitive function.	2018	Nature Communications	Cognition and Mental Health	551
Warren	Genome-wide association analysis identifies novel blood pressure loci and offers biological insights into cardiovascular risk.	2017	Nature genetics	Cardiovascular Disease	541
Roselli	Multi-Ethnic Genome-wide Association Study for Atrial Fibrillation.	2018	Nature Genetics	Cardiovascular	522
Grotzinger	Genomic structural equation modelling provides insights into the multivariate genetic architecture of complex traits.	2019	Nature Human Behaviour	Methodology	521
Liu	Biological and clinical insights from genomic analysis of plasma lipids in >300,000 individuals.	2017	Nature	Cardiovascular Disease	511

Wuttke	A catalogue of genetic loci associated with kidney function from analyses of a million individuals.	2019	Nature Genetics	Kidney Disease	509
Day	Genomic analyses identify hundreds of variants associated with age at menarche and support a role for puberty timing in cancer risk.	2017	Nature Genetics	Cancer	466
Kwong	Mental health before and during the COVID-19 pandemic in two longitudinal UK population cohorts.	2020	British Journal of Psychiatry	COVID-19	461
Soler Artigas	Genome-wide association and large-scale follow-up identifies 16 new loci influencing lung function.	2011	Nature Genetics	Respiratory Disease	458
Messner	Ultra-high-throughput clinical proteomics reveals classifiers of COVID-19 infection.	2020	Cell Systems	COVID-19	433
Bethlehem	Brain charts for the human lifespan.	2022	Nature	Brain Charts	416
Davies	Genetic contributions to variation in general cognitive function: a meta-analysis of genome-wide association studies in the CHARGE consortium (N=53,949).	2015	Molecular Psychiatry	Cognition and Mental Health	416
Davies	Genome-wide association study of cognitive functions and educational attainment in UK Biobank (N=112,151).	2016	Molecular Psychiatry	Cognition and Mental Health	370
Clarke	Genome-wide association study of alcohol consumption and genetic overlap with other health-related traits in UK Biobank (N=112,117).	2017	Molecular Psychiatry	Alcohol	368
Schmidt	PCSK9 genetic variants and risk of type 2 diabetes: a Mendelian randomisation study.	2016	The Lancet Diabetes & Endocrinology	Diabetes	366
Day	Large-scale genomic analyses link reproductive aging to hypothalamic signalling, breast cancer susceptibility and BRCA1-mediated DNA repair.	2015	Nature Genetics	Cancer	366
Shrine	New genetic signals for lung function highlight pathways and pleiotropy, and chronic obstructive pulmonary disease associations across multiple ancestries.	2019	Nature Genetics	Cardiovascular	332
Luciano	Association analysis in over 329,000 individuals identifies 116 independent variants influencing neuroticism.	2017	Nature Genetics	Cognition and Mental Health	312
Turcot	Protein-altering variants associated with body mass index implicate pathways that control energy intake and expenditure in obesity.	2017	Nature Genetics	Lifestyle	300
Rietveld	Common genetic variants associated with cognitive performance identified using the proxy-phenotype method.	2014	PNAS 2014	Cognition and Mental Health	296

Surendran	Trans-ancestry meta-analyses identify rare and common variants associated with blood pressure and hypertension.	2016	Nature Genetics	Cardiovascular Disease	294
de Moor	Meta-analysis of Genome-wide Association Studies for Neuroticism, and the Polygenic Association with Major Depressive Disorder.	2015	JAMA Psychiatry	Cognition and Mental Health	293
Christopher son	Large-scale analyses of common and rare variants identify 12 new loci associated with atrial fibrillation.	2017	Nature Genetics	Cardiovascular Disease	290
Wain	Genome-wide association analyses for lung function and chronic obstructive pulmonary disease identify new loci and potential druggable targets.	2017	Nature genetics	Respiratory Disease	283
Brown	Vascular Consequences of Pre-eclampsia.	2015	Journal of Hypertensio n Volume	Cardiovascular Disease	283
Smith, D. J.	Genome-wide analysis of over 106,000 individuals identifies 9 neuroticism-associated loci.	2016	Molecular Psychiatry	Cognition and Mental Health	266
Graham	The power of genetic diversity in genome-wide association studies of lipids.	2021	Nature	GWAS	261
Chen	The Trans-Ancestral Genomic Architecture of Glycaemic Traits.	2021	Nature Genetics	Other	250
Tin	Target genes, variants, tissues and transcriptional pathways influencing human serum urate levels.	2019	Nature Genetics	Kidney Disease	250
Schumann	KLB is associated with alcohol drinking, and its gene product β-Klotho is necessary for FGF21 regulation of alcohol preference.	2016	PNAS	Alcohol	226
Abul-Husn	Personalized Medicine and the Power of Electronic Health Records.	2019	Cell	Ethical, Legal and Social Issues	218
Clarke	Common polygenic risk for autism spectrum disorder (ASD) is associated with cognitive ability in the general population.	2015	Molecular Psychiatry	Cognition and Mental Health	217
Wessel	Low-frequency and rare exome chip variants associate with fasting glucose and type 2 diabetes susceptibility.	2015	Nature Communicat ions	Diabetes	215
Welsh	Cardiac Troponin T and Troponin I in the general population: contrasting genetic determinants and outcomes.	2019	Circulation	Cardiovascular	214
Graff	Genome-wide physical activity interactions in adiposity — a meta-analysis of 200,452 adults.	2017	PLoS Genetics	Obesity	200
Justice	Genome-Wide Meta-Analysis of 241,258 Adults Accounting for Smoking Behaviour Identifies Novel Loci for Obesity Traits.	2017	Nature Communicat ions	Smoking, Obesity	200
Marioni	Molecular genetic contributions to socioeconomic status and intelligence.	2014	Intelligence	Cognition and Mental Health	196

Power	Genome-wide Association for Major Depression Through Age at Onset Stratification: Major Depressive Disorder Working Group of the Psychiatric Genomics Consortium.	2016	Biological Psychiatry	Cognition and Mental Health	193
