# Supplementary material for: Generation Scotland: an update on Scotland’s longitudinal family health study
Source: BMJ Open. 2024 Jun 21;14(6):e084719. doi: 10.1136/bmjopen-2024-084719 (PMC11340249; doi:10.1136/bmjopen-2024-084719)
Supplement: Author's manuscript [file bmjopen-2024-084719.draft_revisions.pdf]

BMJ Open

BMJ Open is committed to open peer review. As part of this commitment we make the peer review history of every article we publish publicly available.

When an article is published we post the peer reviewers' comments and the authors' responses online. We also post the versions of the paper that were used during peer review. These are the versions that the peer review comments apply to.

The versions of the paper that follow are the versions that were submitted during the peer review process. They are not the versions of record or the final published versions. They should not be cited or distributed as the published version of this manuscript.

BMJ Open is an open access journal and the full, final, typeset and author-corrected version of record of the manuscript is available on our site with no access controls, subscription charges or pay-per-view fees (http://bmjopen.bmj.com).

If you have any questions on BMJ Open's open peer review process please email info.bmjopen@bmj.com

BMJ Open

Cohort Profile: Generation Scotland – an update on Scotland's longitudinal family health study

Journal:	*BMJ Open*
Manuscript ID	bmjopen-2024-084719
Article Type:	Cohort profile
Date Submitted by the Author:	26-Jan-2024
Complete List of Authors:	Milbourn, Hannah; The University of Edinburgh Centre for Genomic and Experimental Medicine, Institute of Genetics and Cancer<br>McCartney, Daniel; The University of Edinburgh Centre for Genomic and Experimental Medicine, Institute of Genetics and Cancer<br>Richmond, Anne; The University of Edinburgh Centre for Genomic and Experimental Medicine, Institute of Genetics and Cancer<br>Campbell, Archie; The University of Edinburgh Centre for Genomic and Experimental Medicine, Institute of Genetics and Cancer<br>Whalley, Heather; The University of Edinburgh Centre for Genomic and Experimental Medicine, Institute of Genetics and Cancer<br>Flaig, Robin; The University of Edinburgh Usher Institute of Population Health Sciences and Informatics, Centre for Medical Informatics<br>Robertson, Sarah; The University of Edinburgh Centre for Genomic and Experimental Medicine, Institute of Genetics and Cancer<br>Fawns-Ritchie, Chloe; University of Dundee Division of Psychology, School of Humanities, Social Sciences and Law; The University of Edinburgh Psychology Department<br>Hayward, Caroline; The University of Edinburgh Centre for Genomic and Experimental Medicine, Institute of Genetics and Cancer<br>Marioni, Riccardo; The University of Edinburgh Centre for Genomic and Experimental Medicine, Institute of Genetics and Cancer<br>McIntosh, Andrew; The University of Edinburgh Division of Psychiatry<br>Porteous, David; The University of Edinburgh Centre for Genomic and Experimental Medicine, Institute of Genetics and Cancer<br>Sudlow, Cathie; The University of Edinburgh Usher Institute of Population Health Sciences and Informatics
Keywords:	EPIDEMIOLOGY, GENETICS, Electronic Health Records, PUBLIC HEALTH

SCHOLARONE™
Manuscripts

I, the Submitting Author has the right to grant and does grant on behalf of all authors of the Work (as defined in the below author licence), an exclusive licence and/or a non-exclusive licence for contributions from authors who are: i) UK Crown employees; ii) where BMJ has agreed a CC-BY licence shall apply, and/or iii) in accordance with the terms applicable for US Federal Government officers or employees acting as part of their official duties; on a worldwide, perpetual, irrevocable, royalty-free basis to BMJ Publishing Group Ltd ("BMJ") its licensees and where the relevant Journal is co-owned by BMJ to the co-owners of the Journal, to publish the Work in this journal and any other BMJ products and to exploit all rights, as set out in our licence.

The Submitting Author accepts and understands that any supply made under these terms is made by BMJ to the Submitting Author unless you are acting as an employee on behalf of your employer or a postgraduate student of an affiliated institution which is paying any applicable article publishing charge ("APC") for Open Access articles. Where the Submitting Author wishes to make the Work available on an Open Access basis (and intends to pay the relevant APC), the terms of reuse of such Open Access shall be governed by a Creative Commons licence – details of these licences and which Creative Commons licence will apply to this Work are set out in our licence referred to above.

Other than as permitted in any relevant BMJ Author's Self Archiving Policies, I confirm this Work has not been accepted for publication elsewhere, is not being considered for publication elsewhere and does not duplicate material already published. I confirm all authors consent to publication of this Work and authorise the granting of this licence.

For peer review only

Cohort Profile: Generation Scotland – an update on Scotland's longitudinal family health study

Hannah Milbourn, Daniel McCartney, Anne Richmond, Archie Campbell, Heather Whalley, Robin Flaig, Sarah Robertson, Chloe Fawns-Ritchie, Caroline Hayward, Riccardo E Marioni, Andrew M McIntosh, David J Porteous, Cathie Sudlow

Ms Hannah Milbourn

Centre for Genomic and Experimental Medicine, Institute of Genetics and Cancer, University of Edinburgh, Western General Hospital, Edinburgh, UK, EH4 2XU

Hannah.Milbourn@ed.ac.uk

ORCID: 0000-0003-4479-8635

Dr Daniel McCartney

Centre for Genomic and Experimental Medicine, Institute of Genetics and Cancer, University of Edinburgh, Western General Hospital, Edinburgh, UK, EH4 2XU

Daniel.McCartney@ed.ac.uk

ORCID: 0000-0003-3242-0360

Ms Anne Richmond

Centre for Genomic and Experimental Medicine, Institute of Genetics and Cancer, University of Edinburgh, Western General Hospital, Edinburgh, UK, EH4 2XU

Anne.Richmond@ed.ac.uk

ORCID: 0000-0002-9504-510X

Mr Archie Campbell

Centre for Genomic and Experimental Medicine, Institute of Genetics and Cancer, University of Edinburgh, Western General Hospital, Edinburgh, UK, EH4 2XU

Archie.Campbell@ed.ac.uk

ORCID: 0000-0003-0198-5078

Professor Heather Whalley

Centre for Genomic and Experimental Medicine, Institute of Genetics and Cancer, University of Edinburgh, Western General Hospital, Edinburgh, UK, EH4 2XU

Heather.Whalley@ed.ac.uk

ORCID: 0000-0002-4505-8869

For peer review only

Ms Robin Flaig

Centre for Medical Informatics, Usher Institute, University of Edinburgh, Nine, Edinburgh Bioquarter, 9 Little France Road, Edinburgh, UK, EH16 4UX

Robin.Flaig@ed.ac.uk

ORCID: 0000-0001-8007-1279

Ms Sarah Robertson

Centre for Genomic and Experimental Medicine, Institute of Genetics and Cancer, University of Edinburgh, Western General Hospital, Edinburgh, UK, EH4 2XU

Sarah.Robertson@ed.ac.uk

Dr Chloe Fawns-Ritchie

Division of Psychology, School of Humanities, Social Sciences and Law, University of Dundee, Scrymgeour Building, Dundee, UK, DD1 4HN

Department of Psychology, University of Edinburgh, 7 George Square, Edinburgh, UK, EH6 5PQ

cfawnsritchie001@dundee.ac.uk

ORCID: 0000-0002-7493-2228

Professor Caroline Hayward

Centre for Genomic and Experimental Medicine, Institute of Genetics and Cancer, University of Edinburgh, Western General Hospital, Edinburgh, UK, EH4 2XU

Caroline.Hayward@ed.ac.uk

ORCID: 0000-0002-9405-9550

Professor Riccardo E Marioni

Centre for Genomic and Experimental Medicine, Institute of Genetics and Cancer, University of Edinburgh, Western General Hospital, Edinburgh, UK, EH4 2XU

Riccardo.Marioni@ed.ac.uk

ORCID: 0000-0003-4430-4260

Professor Andrew M McIntosh

Division of Psychiatry, University of Edinburgh, Royal Edinburgh Hospital, Edinburgh, UK, EH10 5HF

Andrew.McIntosh@ed.ac.uk

ORCID: 0000-0002-0198-4588

Professor David J Porteous

For peer review only

Centre for Genomic and Experimental Medicine, Institute of Genetics and Cancer, University of Edinburgh, Western General Hospital, Edinburgh, UK, EH4 2XU

David.Porteous@ed.ac.uk

ORCID: 0000-0003-1249-6106

Professor Cathie LM Sudlow

Centre for Medical Informatics, Usher Institute, University of Edinburgh, Nine, Edinburgh Bioquarter, 9 Little France Road, Edinburgh, UK, EH16 4UX

Cathie.Sudlow@ed.ac.uk

ORCID: 0000-0002-7725-7520

For peer review only

Abstract

Purpose: Generation Scotland (GS) is a large family-based cohort study established as a longitudinal resource for research into the genetic, lifestyle and environmental determinants of physical and mental health. It comprises extensive genetic, sociodemographic and clinical data from volunteers in Scotland.

Participants: A total of 24,084 adult participants, including 5,501 families, were recruited between 2006 and 2011. Participants completed a health questionnaire and attended an in-person clinic visit where detailed baseline data was collected on lifestyle information, cognitive function, personality traits and mental and physical health. Genotype array data is available for 20,026 (83%) participants, and blood-based DNA methylation (DNAm) data for 18,869 (78%). Linkage to routine NHS datasets has been possible for 93% (N=22,402) of the cohort, creating a longitudinal resource that includes primary care, hospital attendance, prescription and mortality records. Multimodal brain imaging is available in 1,069 individuals.

Findings to date: GS has been widely used by researchers across the world to study the genetic and environmental basis of common complex diseases. Over 350 peer-reviewed papers have been published using GS data, contributing to research areas such as ageing, cancer, cardiovascular disease and mental health. Re-contact studies have built on the GS cohort to collect additional prospective data to study chronic pain, major depressive disorder and COVID-19.

Future plans: To create a larger, richer, longitudinal resource, "Next Generation Scotland" (NGS) launched in May 2022 to expand the existing cohort by a target of 20,000 additional volunteers, now including anyone aged 12 years upwards. New participants complete online consent and questionnaires and provide postal saliva samples for DNA, from which genotype and salivary DNA methylation array data will be generated. Up to date information about the cohort data and how to access it can be found on the GS website (www.generationscotland.org).

For peer review only

Strengths and Limitations

- The GS cohort has a wide range of demographic, lifestyle, health, and genetic data, and includes participants from a wide range of sociodemographic backgrounds.
- Linkage to a variety of longstanding, routine NHS datasets, which are particularly rich and diverse in Scotland, creates a breadth of longitudinal phenotype information.
- Planned linkages to medical images and radiology reports from across the NHS in Scotland will enrich the linked health data, while additional linkages to administrative data (e.g., education records) will broaden the information on participants and support research on the wider determinants of health and well-being.
- The use of self-report questionnaires is associated with inherent biases; however, these are minimised by using validated questionnaires applied widely in research and confirmation of outcomes through linkage to medical records.

Introduction

Generation Scotland (GS) is a longitudinal health study established as a family- and population-based resource for the study of the genetic, lifestyle and environmental determinants of common complex diseases. Non-communicable diseases, such as cancer, diabetes, stroke, heart, liver and lung disease are the leading cause of morbidity and mortality in Scotland [1,2]. The majority of common health disorders of public health concern are a result of a complex interaction between genes and environment. The GS cohort is rich in genetic and phenotypic information through data collection, sample assays and linkage to routine electronic health records. As a bio-resource for medical research, GS aims to support research to establish the determinants of physical and mental health and improve the prevention, diagnosis and treatment of common diseases.

GS was founded as a multi-institutional, cross-disciplinary collaboration between the Universities of Aberdeen, Dundee, Edinburgh and Glasgow, and the National Health Service (NHS) Scotland, with key resources, expertise and input from the Medical Research Council Human Genetics Unit, the National eScience Centre and the Scottish School of Primary Care. Between 2006 and 2011, over 24,000 adult volunteer participants completed questionnaires, attended a clinic visit or participated by post, and consented to genetic studies, linkage to their medical records and to be re-contacted for future research [3].

In 2022 GS launched Next Generation Scotland (NGS), aiming to expand the existing cohort by recruiting 20,000 new participants, newly including 12-17 year olds, meeting an unmet need to study adolescent health. Data is collected via an online questionnaire and saliva sample collection by post.

An earlier cohort profile paper described the baseline recruitment [4]. Here we report data enrichment of the cohort, including new biological data, longitudinal data linkage and re-contact studies. We highlight the extent and nature of the data now available to researchers, summarise the use and impact of GS since commencement in 2006, and outline the current and future plans for NGS.

For peer review only

Cohort Description

Participant recruitment

The original GS: Scottish Family Health Study (SFHS) protocol and baseline data profile have been described previously [3,4]. Briefly, potential participants aged 35-65 years (study probands) were selected from lists of collaborating general medical practices in Scotland. They were invited to participate in the study and asked to identify at least one adult (18+ years old) first-degree relative to invite to the study. This included volunteers from the Glasgow and Tayside areas from 2006-2011 and was extended to include Ayrshire, Arran and Grampian in 2010. Participants completed a pre-clinic questionnaire (PCQ) before attending a research clinic in Glasgow, Dundee, Perth, Aberdeen or Kilmarnock. In total, 126,000 individuals were invited to participate, of whom 6,665 responded and met the study criteria (response rate of 5.3%). An additional 17,419 family members were recruited via these probands. The original GS:SFHS cohort therefore consists of 24,084 participants.

Baseline data collection

All 24,084 participants completed a PCQ collecting a range of demographic, social characteristics, personal behaviours, and self-reported health data. Information collected included smoking status, alcohol consumption, and personal and family disease history. Information was also collected on the birthplaces, by local council area, of participants and their parents and grandparents born in Scotland. In 2009, revisions were made to several questions within the PCQ for machine readability; the period prior to this is was termed phase 1 (N=9,967) and the period thereafter phase 2 (N=14,117).

Most participants (21,476) also attended a research clinic, where physical measurements included height, weight, heart rate, systolic and diastolic blood pressure, electrocardiogram (ECG), and body composition analysis. Standardised and well-validated assessments of cognitive function, personality, and mental health included the 28-item General Health Questionnaire, Eysenck personality questionnaire, Structured Clinical Interview for DSM Disorders, Mood Disorder Questionnaire, Schizotypal Personality Questionnaire, Mill Hill Vocabulary test and WAIS-III logical memory test. All baseline measures collected are reported in the previous cohort profile [4].

Cohort characteristics at recruitment

Of the 24,084 participants within the original GS:SFHS cohort, 59% are female, with an average age at recruitment of 49 years. In total, 87% of participants were born in Scotland and 97% born within the UK. The cohort includes a range of socio-demographic characteristics, although compared to the Scottish population participants have a higher education level and lower deprivation index (Table 1).

Family groups of at least two first-degree relatives were identified and assigned a shared family identity number. Pedigrees were constructed using relationship information provided by study participants and validated with genetic kinship information following genotyping. The cohort contained 1,361 singletons (with no relatives in the study) and 5,501 families of at least two people, with a mean size of 4.1 family members.

For peer review only

Longitudinal Data linkage

Linkage to extensive and longstanding NHS Scotland records, both retrospective and prospective, creates a longitudinal cohort from baseline. The linkage data available within GS has not previously been described. At the time of writing, participants have up to 16-years follow-up data since recruitment in 2006. Routine NHS data is obtained through collaboration with the Health Informatics Centre (HIC) at the University of Dundee, with linkage performed using the community health index (CHI) number. CHI numbers are used across NHS Scotland services and are unique to each GP-registered individual living in Scotland. In total, 93% of GS participants consented to linkage and had a CHI number available. For individuals with CHI linkage, 89% also have genome-wide genotype data available (see Laboratory Samples and Molecular Assays below).

Table 2 shows the range of datasets linked to the GS cohort, their periods of coverage and the numbers of participants with linked data available for each. Additional details are provided in Appendix A. Beyond linkages to hospital episodes, primary care, cancer and death registries and community electronic prescribing, GS has linkage to a range of other datasets, for example routine laboratory tests, dental data (from the Management Information & Dental Accounting System, MIDAS) and the Scottish Drug Misuse Database (SDMD), offering unique phenotype information distinct from other population-based cohort research resources. COVID-19 testing, diagnoses and vaccination records are also available for the period of 2020 to 2022.

Regular data refreshes are received and new datasets are added to enhance and continue the follow-up of participants over time. Planned additional linkages include incorporating NHS Scotland routine NHS radiology images, including X-rays, computerised tomography (CT) and magnetic resonance imaging (MRI) scans (Scottish Medical Imaging, SMI), imaging reports, and retinal scans, which will provide new research opportunities not available in other population-based cohorts. Text-based radiology report linkage has already been applied to a study of stroke phenotyping in GS participants [5].

Cohort morbidities

Participant self-reported disease prevalence (at recruitment) is shown in Table 3 alongside longitudinal data on morbidities obtained through data linkage to primary care (GP) data, Scottish morbidity records (SMR) and National Records of Scotland (NRS) death records. Data is available up to 2020 for GP records, cancer registries and 2022 for hospital admissions (SMR01) and mortality records. Diagnoses of 3,006 hypertension, 2,197 asthma, 2,371 depression, 2,558 osteoarthritis and 1,701 heart disease cases are reported across all primary and secondary care, and mortality linked data sources.

As an extended example, Figure 1 shows the proportion of diabetes cases captured in secondary care and deaths records as described above and enhanced with additional data sources available within GS (The Scottish Care Information – Diabetes Collaboration (SCI-DC), the Prescribing Information System (PIS) and routine laboratory testing data). A total of 1,861 diabetes (type 1 and 2) cases were recorded in at least one source, cohort prevalence of 7.7%. The SCI-DC captures 74% of all recorded cases. Prescriptions of Metformin Hydrochloride or Insulin within the PIS captured 73% of diabetes cases. Linked routine laboratory testing data contained results for any glycated haemoglobin (HbA1c) tests conducted as an indication of average blood sugar levels. HbA1c level above 6.5% was classified as diabetic (28% of cases captured) [6]. We note that lower proportions of cases within the self-reported source reflects that these were collected at baseline while other

For peer review only

sources extend to 2020/2022. Use of a combination of data sources provides an opportunity to capture a range of cases and develop detailed phenotype definitions.

Laboratory samples and molecular assays

Participants who attended a research clinic also provided biological samples (including blood and urine) for genotyping and other assays (N=23,979). Saliva was provided for DNA extraction by a subset of participants not attending a clinic (2,608 sent a saliva sample by post) and was used for DNA extraction for an additional 984 participants from whom blood could not be obtained (total 3,592). DNA was extracted from blood and saliva for 85% of participants (N= 20,471). Basic biochemistry assays were performed on the baseline serum samples measuring creatinine, glucose, potassium, sodium, urea, and cholesterol levels. Here we provide an update on the genotyping methods conducted since baseline collection.

Genomics

Genome-wide genotyping data is available for 20,026 (83%) of the original GS:SFHS participants [14]. Samples were genotyped using the Illumina HumanOmniExpressExome8v1-2_A and HumanOmniExpressExome-8v1_A and the Beadstudio-Gencall v3 genotype calling algorithm. Quality control measures were implemented, filtering out samples with a call rate of <98% and SNPs with a call rate of <98%, HWE of <1x10-06 and MAF of ≤1%, leaving 20,026 samples and 630,207 SNPs. Phasing of the genotyped SNPs was carried out using SHAPEIT2 [7].

Genetic profiles have been imputed using three different reference panels: 1000 Genomes (1000G) [8], Haplotype Reference Consortium (HRC) [9] and Trans-Omics for Precision Medicine (TOPMed) (Table 4) [10]. After imputation, further quality control procedures have removed duplicate and monomorphic SNPs and those with an imputation quality score of <0.4.

Methylomics

DNA methylation (DNAm) data has been generated using the Illumina HumanMethylationEPIC BeadChip array for 18,869 GS samples at > 850,000 CpG sites, from blood collected at the baseline appointment (2006-2011). At the time of writing, this is the largest DNA methylation dataset from a single population-based cohort. These samples were processed in four batches between 2017 and 2021, and are referred to as set 1 (N=5,087), set 2 (N=459), set 3 (N=4,450) and set 4 (N=8,873). A subsequent genome-wide DNA methylation measurement is also available for 880 individuals across set 2 (N=508) and set 3 (N=372), from additional blood collected between 2015 and 2019. The DNA methylation resource will be described in detail in a separate report. Briefly, quality control was carried out in R using the packages ShinyMethyl and WateRmelon. Probes with a bead count of less than three or a high detection P value (>0.05) in more than 5% of samples were removed. Outlier probes were also removed based on visual inspection of the log median intensity of the methylated versus unmethylated signal per array. Samples were removed where there were sex mismatches or where 1% or more of cytosine-guanine dinucleotides had a high detection P value (>0.05). A superset of 18,869 baseline samples has also been generated from the four individual sets, comprising 831,733 CpGs that passed quality control in all sets.

Proteomics and metabolomics

Protein levels have been quantified in plasma samples from 1,065 participants using the 5k SOMAscan v4 array from SomaLogic Inc. Tandem mass spectrometry (MS/MS) has been performed on a subset of 860 participants' blood samples for which peripheral blood mononuclear cells

For peer review only

(PBMCs) were available. Quantification of 54 urinary metabolite biomarkers in 2,743 GS participants' samples has been conducted by Nightingale Health using nuclear magnetic resonance (NMR).

Re-contact studies

Participants provided broad consent permitting use of data and samples for 'future medical research into health, illness and medical treatment'. This included consent to be re-contacted for new studies, which has led to additional data collections since recruitment, summarised in Table 5. Data from re-contact studies can be linked to GS data and are retained by GS to be made available for other researchers through the GS access process.

The Stratifying Resilience and Depression Longitudinally (STRADL) sub-study built on the GS cohort to subtype major depressive disorder (MDD) based on its aetiology, using detailed clinical, cognitive, and brain imaging assessments. From 2015-2017, 9,905 GS participants completed a remote depression-focused questionnaire (including psychological resilience, coping style, and response to psychological distress) and a subset (N=1,189) attended a face-to-face assessment to conduct cognitive testing, multimodal MRI brain scans (N=1,085) and further bio-sample collection [11].

In 2016, the DOLORisk study enhanced GS to study neuropathic pain (NP). The study received responses to a survey regarding presence or absence of chronic pain and NP from 7,238 of 20,221 members of the GS cohort invited to participate (35.8% response rate), with a follow-up repeat survey (N= 5,292 responses) after 18 months (Table 5) [12].

GS is a member of the European Prevention of Alzheimer's Dementia Consortium (EPAD), an interdisciplinary research initiative with partners across European organisations aiming to improve the understanding of the early stages of Alzheimer's disease [13]. In 2016, 53 GS participants attended a 'screening visit' for the collection of fasting blood samples and a brain scan (MRI) with follow up visits after 6 months, 1 year, 2 years, 3 years, and 4 years.

GS is partnering with Healthy AGeing in Scotland (HAGIS), a study of the health, economic and social circumstances of people over 50 years old in Scotland [14]. HAGIS is part of the Health & Retirement Study family of longitudinal ageing studies which currently consists of longitudinal ageing studies in 16 countries around the world. GS re-contacted 14,891 individuals in 2021-22, with 2,826 (19.0%) taking part in the HAGIS: COVID-19 Impact & Recovery Study.

Additional data collections conducted by the GS team include the CovidLife surveys launched in April 2020 in response to the COVID-19 pandemic. The aim was to determine the impact of the pandemic on health and well-being. In total, 18,518 adult members of the UK public, including 4,968 GS participants, participated in the surveys. Three CovidLife surveys [15] and a Rural CovidLife survey [16] specific to rural Scottish volunteers (Total N=3,365, GS participants N=712). In addition, three TeenCovidLife surveys [17], for young people aged 12-18 (N = 7,058), were run between April 2020 and June 2021. GS was part of the National Core Studies Longitudinal Health and Wellbeing programme established as part of the UK's pandemic response, including the coronavirus post-acute long-term effects: constructing an evidence base (CONVALESCENCE) long COVID study [18]. GS is also a participating cohort in COVIDMENT, a large-scale collaborative project between Northern European countries using data-rich population-based registry resources, biobanks, and ongoing questionnaire data to further understanding of the mental health impact of the COVID pandemic [19].

For peer review only

New Recruitment and Data Collection Plans

In 2019, funding was obtained from the Wellcome Trust to expand the GS cohort using online recruitment, consent and data collection, alongside remote postal saliva sample collection, and extension to younger individuals (>12 years). Because of the COVID-19 outbreak in 2020, field studies other than those directly relating to the pandemic were paused. Active recruitment of new volunteers to join GS started in May 2022. NGS aims to recruit 20,000 new participants and will use established methods for linkage to routine NHS data to create a larger, richer, longitudinal resource. Original GS cohort members are also being contacted with the option to move online to complete new questionnaires and invite family members to join the next phase of the study. New recruits and returning GS members will complete a baseline questionnaire to collect lifestyle measures and medical history. Saliva samples are being collected by post for genotyping of new participants. At the time of writing over 9,000 new participants have been recruited, adding to the 2006-2011 cohort recruits.

Adolescence and early adulthood are critical periods in the development of mental and physical health [20]. The extension to younger individuals, along with potentially other family members, will make the cohort a valuable resource for research into genetic and environmental determinants of health among adolescents and young adults. There are few comparable genetic cohorts using routine data linkage in young people. Early approved studies are planned to focus on mental health, sleep and loneliness in this age group.

New questionnaires will be regularly added to the online portal to enable ongoing engagement with participants and collect enhanced data such as cognitive testing. Researchers will be able to submit approved research questions for prospective data collections. Through a broad range of recruitment strategies, participant involvement and engagement and the use of remote data collection, we hope to improve geographic coverage and sociodemographic diversity across Scotland, aiming to engage groups typically under-represented in large-scale studies. Completion of the expansion phase, combined with the original GS participants, should create an overall cohort of over 40,000 individuals across Scotland with rich genetic and phenotypic data.

Participant and Patient Involvement

A key component of the GS:SFHS was to conduct a public consultation programme which was used to ask the public their thoughts on genetics in healthcare and research and use this to develop principles of participation and data access [21,22]. Regular newsletters are distributed to participants to provide updates on the latest cohort information and recent findings. Patient and public involvement and engagement (PPIE) is being developed within the new NGS cohort recruitment. A survey receiving 1,000 responses invited participants to become GS ambassadors in their local area, take part in focus groups and test new questionnaires. These volunteers have already helped with survey testing and provided feedback on recruitment materials. Development of a Young Persons Advisory Group (YPAG) has helped direct teen recruitment activities and will shape future GS health research with teenagers themselves.

Findings to Date

The GS cohort has facilitated research contributions to a wide range of health conditions and scientific areas including aging, cancer, cardiovascular disease, mental health and the role of DNA methylation in understanding and predicting disease. Over 350 papers have been published using GS data (Figure 2). Appendix E lists the 50 most cited papers using GS data and a full and growing

For peer review only

publication list can be found on the GS website (https://www.ed.ac.uk/generation-scotland/what-found/publications). Examples of some key contributions are summarised below.

Welsh et al used residual blood samples from GS participants (N=19,501) to assay cardiac troponin T (cTnT) and troponin I (cTnI), proteins essential for heart contraction, and to investigate their association with cardiovascular outcomes [23]. The research team identified deaths or hospitalisations of interest using Scotland's morbidity records and deaths data from GS recruitment to September 2017. They found that cTnT and cTnI were both associated with heart failure and cardiovascular disease death. Individuals with high levels of cTnT were more likely to suffer from heart disease, stroke or other heart conditions. Troponin level testing is inexpensive, and this study demonstrated the potential benefit of testing for future health screening.

Given its size, the GS DNA methylation resource is well-placed to serve as a training dataset for the development of risk predictors. Cheng et al used GS methylation data to develop and validate a model for 10-year risk prediction of type 2 diabetes [24]. They combined standard risk prediction information such as age, sex, body mass index (BMI) and family history of the disease with DNA methylation data, which improved prediction for likelihood of developing diabetes. The results were tested using a hypothetical screening scenario of 10,000 people, which correctly classified an additional 449 individuals using methylation data compared with traditional risk factors alone.

Green et al investigated the aetiology of major depressive disorder among individuals from the STRADL re-contact study [25]. They reported the associations of serological and methylomic signatures of C-Reactive Protein (considered to represent acute and chronic measures of inflammation respectively) with depression status/symptoms, and structural neuroimaging phenotypes. The study provided evidence for the involvement of peripheral inflammation in brain morphology and depression symptoms, and demonstrated the combined use of survey, neuroimaging, serological and methylation data from the GS cohort.

GS facilitated a pilot study to investigate the use of newborn blood spots in longitudinal research. Heel prick blood spots are used routinely to test for treatable neo-natal metabolic conditions and have been retained in Scotland for all children born since 1965. Researchers showed that archival blood spots contain enough information to link to the volunteer health records and samples were of sufficient quality to generate biologically meaningful results [26]. For example, epigenetic signatures of perinatal maternal smoking status could be identified. This pilot study confirmed the feasibility of the use of these archived newborn blood spots in a population-level retrospective birth cohort study. It has the potential to scale to a linked collection of 3 million archived blood spots across Scotland, making it one of only two such resources available worldwide [27]. Future work is dependent on a Scottish government led public consultation to review the current pause on research access [28].

For peer review only - http://bmjopen.bmj.com/site/about/guidelines.xhtml

Strengths and Limitations

Important strengths of GS are the breadth of demographic, lifestyle and health factors, and inclusion of participants from a wide range of sociodemographic backgrounds. The cohort is rich in genetic and linkage data. Scotland is ideally suited to a longitudinal cohort study given its comparatively static and stable population and relatively high prevalence of common conditions and adverse lifestyle risk factors [1,2]. The family-based recruitment approach delivers increased kinship among participants and pedigree mapping enables measurement of heritability and familial aggregation of traits.

Linkage to a variety of routine NHS datasets creates a wealth of research opportunities, while participants' consent for future re-contact studies provides potential for additional data collections. Planned linkages to routine NHS medical images, radiology reports and administrative data, such as education, income and benefits, will provide uniquely rich information about the participants and its relationship with future health and wellbeing, further enhancing the research potential of the cohort.

There are some limitations of the GS cohort. The cohort is relatively small, by contemporary standards for population-based cohorts, which can limit the statistical power to address some research questions definitively (e.g., to study rare diseases or small effect sizes). However, this issue can often be addressed through joint analyses with other population-based cohorts and participation in genetic data consortia, which GS actively contributes to. The current expansion of the cohort will also help to address this limitation. Many phenotypes are assessed using self-reported measures. The bias of self-reported measures is minimised in GS by using validated questionnaires applied widely in research and confirmation of outcomes through linkage to medical records. Compared to the Scottish population, individuals in the cohort are generally older, more likely to be female and less socially deprived. However, it is hoped that increased diversity of the cohort will be achieved with the current expansion to reach a total cohort size of over 40,000 individuals in Scotland. GS aims to be the UK's largest multi-generational longitudinal life-course study of genetic, epigenetic, clinical, lifestyle and environmental health determinants.

Data Access and Collaborations

Researchers can submit proposals to access GS data and samples through our website (https://www.ed.ac.uk/generation-scotland/for-researchers). This also includes data from re-contact studies which can be accessed through a single application to GS. Research proposals are subject to review by the GS access process, under the guidance of the Scientific Steering Committee, based on criteria set out in the management, access and publications policy. We welcome proposals for data and sample access, and for prospective data collections using the NGS online portal. Further information about the cohort, details of the application process, and conditions for access are available at the study website.

Generation Scotland also collaborates with – and makes its data and/or metadata available via – the Dementias Platform UK (DPUK), UK Longitudinal Linkage Collaboration (UK LLC), CLOSER, BC Platforms and Health Data Research UK (HDR UK) Innovation Gateway. Access requests can be made through DPUK and UK LLC using the standard GS access process as well as directly to GS. All applications via these platforms are reviewed by the GS access process. Study metadata is available through CLOSER Discovery, BC Platforms and HDR UK.

GS genetic data has contributed to large-scale consortia including Cohorts for Heart and Aging Research in Genomic Epidemiology (CHARGE)[29], Chronic Kidney Disease Genetics (CKDGen)[30],

For peer review only - http://bmjopen.bmj.com/site/about/guidelines.xhtml

Genetic Investigation of ANthropometric Traits (GIANT)[31], SpiroMeta[32], Global Biobank Meta-analysis Initiative (GBMI)[33], COVID-19 host genetics initiative (HGI)[34], Global Lipids Genetics Consortium (GLGC)[35] and The Psychiatric Genomics Consortium (PGC) [36].

Funding

GS:SFHS was funded by a grant from the Chief Scientist Office of the Scottish Government Health Directorates [CZD/16/6] and the Scottish Funding Council [HR03006]. Genotyping of the GS:SFHS samples was carried out by the Genetics Core Laboratory at the Edinburgh Clinical Research Facility, University of Edinburgh, Scotland and was funded by the Medical Research Council UK and the Wellcome Trust (Wellcome Trust Strategic Award "STratifying Resilience and Depression Longitudinally" (STRADL) Reference 104036/Z/14/Z). NGS is funded by the Wellcome Trust (216767/Z/19/Z). DNAm analysis was funded by the Wellcome Trust (Reference 220857/Z/20/Z).

Competing Interests

Riccardo E Marioni is a scientific advisor to Optima Partners and the Epigenetic Clock Development Foundation. Daniel McCartney is a part-time employee of Optima Partners Ltd.

Acknowledgements

We are grateful to all the people and their families who have taken part in GS to date, the general practitioners and the Scottish School of Primary Care for their help in recruiting them, the GS Scientific Steering Committee, and the whole GS team, which includes interviewers, computer and laboratory technicians, clerical workers, research scientists, volunteers, managers, receptionists, healthcare assistants and nurses. A list of current staff working on developing and maintaining the GS resource and members of the Scientific Steering Committee is provided in Appendix F.

For peer review only

References

1 The Scottish Government. The Scottish Health Survey 2021 Edition. 2021. http://www.gov.scot/publications/scottish-health-survey-2018-volume-1-main-report/ (accessed 29 March 2023)

2 The Scottish Government. Public Health Priorities for Scotland. 2018. https://www.gov.scot/binaries/content/documents/govscot/publications/corporate-report/2018/06/scotlands-public-health-priorities/documents/00536757-pdf/00536757-pdf/govscot%3Adocument/00536757.pdf (accessed 29 March 2023)

3 Smith BH, Campbell H, Blackwood D, *et al.* Generation Scotland: The Scottish Family Health Study; a new resource for researching genes and heritability. *BMC Med Genet*. 2006;7:1–9.

4 Smith BH, Campbell A, Linksted P, *et al.* Cohort Profile: Generation Scotland: Scottish Family Health Study (GS:SFHS). The study, its participants and their potential for genetic research on health and illness. *Int J Epidemiol*. 2013;42:689–700.

5 Davidson EM, Casey A, Grover C, *et al.* The epidemiological characteristics of stroke phenotypes defined with ICD-10 and free-text: a cohort study linked to electronic health records. *medRxiv*. 2023;2023.04.03.23288096.

6 Bennett CM, Guo M, Dharmage SC. HbA(1c) as a screening tool for detection of Type 2 diabetes: a systematic review. *Diabet Med*. 2007;24:333–43.

7 Delaneau O, Howie B, Cox AJ, *et al.* Haplotype estimation using sequencing reads. *Am J Hum Genet*. 2013;93:687–96.

8 Altshuler DM, Durbin RM, Abecasis GR, *et al.* An integrated map of genetic variation from 1,092 human genomes. *Nature 2012 491:7422*. 2012;491:56–65.

9 McCarthy S, Das S, Kretzschmar W, *et al.* A reference panel of 64,976 haplotypes for genotype imputation. *Nat Genet*. 2016;48:1279–83.

10 Taliun D, Harris DN, Kessler MD, *et al.* Sequencing of 53,831 diverse genomes from the NHLBI TOPMed Program. *Nature 2021 590:7845*. 2021;590:290–9.

11 Habota T, Sandu AL, Waiter GD, *et al.* Cohort profile for the STratifying Resilience and Depression Longitudinally (STRADL) study: A depression-focused investigation of Generation Scotland, using detailed clinical, cognitive, and neuroimaging assessments. *Wellcome Open Res*. 2021;4. doi: 10.12688/WELLCOMEOPENRES.15538.2

12 Hébert HL, Veluchamy A, Baskozos G, *et al.* Cohort profile: DOLORisk Dundee: a longitudinal study of chronic neuropathic pain. *BMJ Open*. 2021;11:e042887.

13 Solomon A, Kivipelto M, Molinuevo JL, *et al.* European Prevention of Alzheimer's Dementia Longitudinal Cohort Study (EPAD LCS): study protocol. *BMJ Open*. 2018;8:e021017.

14 Douglas E, Rutherford A, Bell D. Pilot study protocol to inform a future longitudinal study of ageing using linked administrative data: Healthy AGeing in Scotland (HAGIS). *BMJ Open*. 2018;8:e018802.

For peer review only

15 Fawns-Ritchie C, Altschul DM, Campbell A, *et al.* CovidLife: a resource to understand mental health, well-being and behaviour during the COVID-19 pandemic in the UK. *Wellcome Open Res*. 2021;6:176.

16 Stevenson AJ, Huggins CF, Forbes A, *et al.* RuralCovidLife: A new resource for the impact of the pandemic on rural Scotland. *Wellcome Open Res*. 2022;6:317.

17 Huggins CF, Fawns-Ritchie C, Altschul DM, *et al.* TeenCovidLife: a resource to understand the impact of the COVID-19 pandemic on adolescents in Scotland. *Wellcome Open Res*. 2021;6. doi: 10.12688/WELLCOMEOPENRES.17252.2

18 Bowyer RCE, Huggins C, Toms R, *et al.* Characterising patterns of COVID-19 and long COVID symptoms: Evidence from nine UK longitudinal studies. *Eur J Epidemiol*. 2023;38:199–210.

19 Unnarsdóttir AB, Lovik A, Fawns-Ritchie C, *et al.* Cohort Profile: COVIDMENT: COVID-19 cohorts on mental health across six nations. *Int J Epidemiol*. 2022;51:e108–22.

20 Solmi M, Radua J, Olivola M, *et al.* Age at onset of mental disorders worldwide: large-scale meta-analysis of 192 epidemiological studies. *Mol Psychiatry*. 2022;27:281–95.

21 Haddow G, Cunningham-Burley S, Bruce A, *et al.* Generation Scotland: consulting publics and specialists at an early stage in a genetic database's development. *Crit Public Health*. 2008;18:139–49.

22 Haddow G, Cunningham-Burley S, Murray L. Can the governance of a population genetic data bank effect recruitment? Evidence from the public consultation of Generation Scotland. *Public Understanding of Science*. 2011;20:117–29.

23 Welsh P, Preiss D, Hayward C, *et al.* Cardiac Troponin T and Troponin I in the General Population. *Circulation*. 2019;139:2754–64.

24 Cheng Y, Gadd DA, Gieger C, *et al.* Development and validation of DNA methylation scores in two European cohorts augment 10-year risk prediction of type 2 diabetes Check for updates. *Nat Aging*. 2023;3:450–8.

25 Green C, Shen X, Stevenson AJ, *et al.* Structural brain correlates of serum and epigenetic markers of inflammation in major depressive disorder. *Brain Behav Immun*. 2021;92:39–48.

26 Cunningham-Burley S, McCartney DL, Campbell A, *et al.* Feasibility and ethics of using data from the Scottish newborn blood spot archive for research. *Communications Medicine 2022 2:1*. 2022;2:1–8.

27 Nørgaard-Pedersen B, Hougaard DM. Storage policies and use of the Danish Newborn Screening Biobank. *J Inherit Metab Dis*. 2007;30:530–6.

28 The Scottish Government. Guthrie Cards in Scotland: Ethical, Legal and Social Issues. 2013. https://www.gov.scot/publications/guthrie-cards-scotland-ethical-legal-social-issues/documents/ (accessed 12 October 2023)

29 Psaty BM, O'Donnell CJ, Gudnason V, *et al.* Cohorts for Heart and Aging Research in Genomic Epidemiology (CHARGE) Consortium. *Circ Cardiovasc Genet*. 2009;2:73–80.

30 Köttgen A, Pattaro C. The CKDGen Consortium: ten years of insights into the genetic basis of kidney function. *Kidney Int*. 2020;97:236–42.

For peer review only

31 Yengo L, Vedantam S, Marouli E, *et al.* A saturated map of common genetic variants associated with human height. *Nature*. 2022;610:704–12.

32 Repapi E, Sayers I, Wain L V, *et al.* Genome-wide association study identifies five loci associated with lung function. *Nat Genet*. Published Online First: 2009. doi: 10.1038/ng.501

33 Zhou W, Kanai M, Wu K-HH, *et al.* Global Biobank Meta-analysis Initiative: Powering genetic discovery across human disease. *Cell Genomics*. 2022;2. doi: 10.1016/j.xgen.2022.100192

34 The COVID-19 Host Genetics Initiative. The COVID-19 Host Genetics Initiative, a global initiative to elucidate the role of host genetic factors in susceptibility and severity of the SARS-CoV-2 virus pandemic. *European Journal of Human Genetics 2020 28:6*. 2020;28:715–8.

35 Graham SE, Clarke SL, Wu K-HH, *et al.* The power of genetic diversity in genome-wide association studies of lipids. *Nature*. 2021;600:675–9.

36 Sullivan PF, Agrawal A, Bulik CM, *et al.* Psychiatric Genomics: An Update and an Agenda. *Am J Psychiatry*. 2018;175:15.

37 National Records of Scotland. Scotland 2011 census table: DC1117SC - Age by sex. 2011. https://www.scotlandscensus.gov.uk/documents/2011-census-table-data-scotland/

38 National Records of Scotland. Scotland 2011 census table: LC2101SC - Ethnic group by age. 2011. https://www.scotlandscensus.gov.uk/documents/2011-census-table-data-scotland/

39 National Records of Scotland. Scotland 2011 census table: LC5102SC - Highest level of qualification by sex by age. 2011. https://www.scotlandscensus.gov.uk/documents/2011-census-table-data-scotland/

40 Roughsedge E. Population Estimates by Scottish Index of Multiple Deprivation (SIMD). 2022. https://www.nrscotland.gov.uk/statistics-and-data/statistics/statistics-by-theme/population/population-estimates/2011-based-special-area-population-estimates/population-estimates-by-simd-2016

41 Roughsedge E. Population Estimates by Urban Rural Classification (2011 Data Zone based). 2022. https://www.nrscotland.gov.uk/statistics-and-data/statistics/statistics-by-theme/population/population-estimates/2011-based-special-area-population-estimates/population-estimates-by-urban-rural-classification

42 The Scottish Government. Scotland's Labour Market: People, Places and Regions – Protected Characteristics. Statistics from the Annual Population Survey 2021. 2022. https://www.gov.scot/publications/scotlands-labour-market-people-places-regions-protected-characteristics-statistics-annual-population-survey-2021/pages/1/

43 Gadd DA, Hillary RF, McCartney DL, *et al.* Epigenetic scores for the circulating proteome as tools for disease prediction. *bioRxiv*. 2021;8:2020.12.01.404681.

For peer review only

Table 1: Demographic and lifestyle characteristics of GS:SFHS cohort (N=24,084) and comparison to the Scottish population.

[a] Based on 2011 Scottish Census [37–39].

[b] Based on National Records of Scotland (NRS) Population Estimates by Scottish Index of Multiple Deprivation (SIMD) [40].

[c] Based on NRS Scottish Government Urban Rural Classification 2020 [41].

[d] Based on the ONS Annual Population Survey 2021 [42]. Individuals aged 18-64.

[e] Based on The Scottish Health Survey 2021 [1].

Characteristic	N	% (or median and IQR)	Scotland average
Sex			
Female	14,157	58.8%	51.5%[a]
Male	9,927	41.2%	48.5% [a]
Age			
Median Age (interquartile range, IQR)	24,084	49 (36 - 59)	41.3[a]
Ethnicity			
White	22,826	94.8%	96.0% [a]
Other	262	1.1%	4.0% [a]
Missing	996	4.1%	
SIMD quintiles			
5 (Least Deprived)	6,571	27.3%	20.6%[b]
4	5,419	22.5%	21.1% [b]
3	3,437	14.3%	19.8% [b]
2	2,987	12.4%	19.3% [b]
1 (Most Deprived)	2,724	11.3%	19.2% [b]
Missing	2,946	12.2%	
Rural Urban Classification codes			
1 - Large Urban Areas	7,369	30.6%	37.8% [c]
2	6,679	27.7%	33.9% [c]
3	2,032	8.4%	8.6% [c]
4	1,006	4.2%	2.6% [c]
5	2,662	11.1%	11.6% [c]
6 - Remote Rural Areas	1,390	5.8%	5.5% [c]
Missing	2,946	12.2%	
Employment Status (up to 75 years)			
Employed (full or part-time)	14,808	64.6%	73.2% [d]
Unemployed	970	4.2%	3.90% [d]
Retired	3,080	13.4%	
Other	1,808	7.9%	
Missing	2,255	9.8%	
Education - Highest qualification attained			
No qualification	3,145	13.3%	26.8% [a]
Lower secondary school	3,611	15.3%	23.1% [a]
Higher secondary school	2,452	10.4%	14.3% [a]
College level	6,450	27.3%	9.7% [a]
University level	7,330	31.0%	26.1% [a]
Other	624	2.6%	

Missing	1,738	7.2%	
Smoking Status			
Current Smoker	3,997	16.6%	11.3% [e]
Ex-Smoker	6,964	28.9%	23.2% [e]
Non-Smoker	12,227	50.8%	65.6% [e]
Missing	896	3.7%	
Alcohol consumption			
Median alcohol units per week (IQR)	21,737	8 (2 - 15)	6.1 [e]

Table 2: Summary of linked data sources. Total number of participants and proportion of individuals with genome wide genotyping data available for all linked data and their periods of coverage. Linkage dataset coverage ranges from 1975 to 2022.

[a] Scottish national telehealth and telecare organisation.
[b] Scottish Intensive Care Society Audit Group (SICSAG).
[c] National Records of Scotland (NRS).
[d] Scottish Care Information – Diabetes Collaboration (SCI-DC).
[e] Prescribing Information System (PIS). Shaded portion represents incomplete coverage for data up to 2009.
[f] Electronic Communication of Surveillance in Scotland (ECOSS).
[g] Management Information & Dental Accounting System (MIDAS).

For peer review only

	Total Participants	Genome-wide genotype data available N	%
CHI Linkage	22,403	19,960	89.1%
**Scottish Morbidity Records**			
Outpatient Attendance (SMR00)	21,271	19,159	90.1%
General/Acute Inpatient and Day Case (SMR01)	18,249	16,467	90.2%
Maternity Inpatient and Day Case (SMR02)	8,239	7,537	91.5%
Mental Health Inpatient and Day Case (SMR04)	578	516	89.3%
Scottish Cancer Registry (SMR06)	3,606	3,207	88.9%
Scottish Birth Record (SMR11)	3,246	2,864	88.2%
**Primary Care**			
General Practice (GP)	19,676	17,823	90.6%
GP Out of Hours	8,533	7,700	90.2%
NHS24[a]	12,326	11,108	90.1%
Accident and Emergency (A&E)	15,249	13,778	90.4%
**Other Datasets**			
Routine Laboratory Test Results	19,090	17,521	91.8%
ICU Episode Data (SICSAG)[b]	361	324	89.8%
Deaths (NRS deaths data)[c]	1,659	1,376	82.9%
Diabetes Registry (SCI-DC)[d]	1423	1,241	87.2%
Prescription Dispensing (PIS)[e]	21,486	19,347	90.0%
COVID-19 Vaccinations	19,128	17,358	90.7%
COVID-19 Testing (ECOSS)[f]	16,537	14,995	90.7%
Dental (MIDAS)[g]	19,871	17,904	90.1%
Scottish Drug Misuse Database (SDMD)	76	63	82.9%

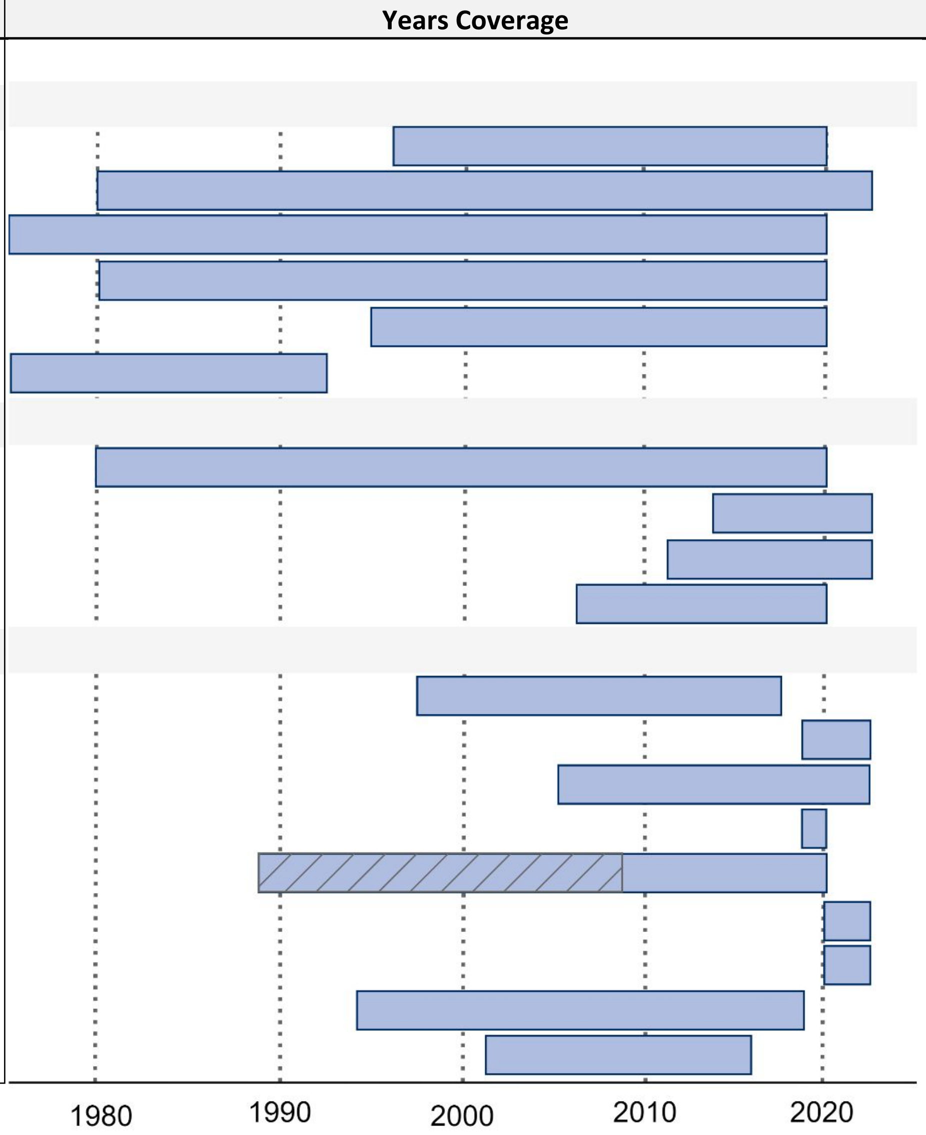

Table 3: Self-reported prevalence of common morbidities at baseline and morbidities in GS participants using linked data up to 2022. ICD and Read Codes were derived from Gadd et al [43] using CALIBER code lists, detailed in full in appendix Tables B-D.

[a] Phase 1 participants (N=9,967) were asked if they have been diagnosed with depression and in phase 2 (N=14,117) if they had been diagnosed with severe depression

[b] Chronic obstructive pulmonary disease (COPD) data for phase 2 only (N=14,117).

[c] Participants were asked if they have been diagnosed with Alzheimer's disease.

[d] Dataset date coverage: GP data (1980-2020), SMR01 (1980-2022), National Records of Scotland (NRS) deaths data (2007-2022), SMR06 (1996-2020), SMR04 (1980-2020).

[e] Deduplicated across data sources.

	**Baseline Self-Reported Morbidities 2006-2011**		**Morbidity Diagnoses from NHS Linked Data Sources 1980-2022[d]**				
**Disease Outcome**	**N**	**% Participants**	**Primary Care (GP Data)**	**General/Acute Inpatient and Day Case (SMR01)**	**NRS Deaths Data**	**Scottish Cancer Registry (SMR06)**	**Total[e]**
Hypertension	3,257	13.8%	1,669	2,436	160		3,464
Asthma	2,652	11.2%	1,719	1,111	10		2,292
Depression[a]	2,196	9.3%	2,139	334	<10		2,346
Osteoarthritis	1,783	7.5%	1,861	1,639	<10		2,817
Heart Disease	935	3.9%	960	1,673	342		1,885
Diabetes	804	3.4%	536	968	149		1,200
Rheumatoid arthritis	431	1.8%	199	207	16		322
Stroke	352	1.5%	514	434	72		753
Breast Cancer	345	1.5%	355	596	82	541	650
COPD[b]	276	1.2%	554	693	150		952
Bowel Cancer	142	0.6%	155	245	39	213	298
Prostate Cancer	104	0.4%	164	271	53	252	323
Lung Cancer	63	0.3%	94	205	152	174	234
Dementia[c]	41	0.2%	198	245	175		356
COVID-19			<10	145	38		162

Table 4: GS genotyping and imputation summary for three imputation panels.

	1000G	HRC	TOPMed
Reference panel version	1000G Phase 1 v3	HRC.r1-1	TOPMed Freeze5
Imputation software	IMPUTE2	PBWT	Minimac4
Build	GRCh37	GRCh37	GRCh38
Number of SNPs (post QC)	9,438,897	24,161,581	64,616,987
Indels	✕	✕	✓

Table 5: Summary of re-contact studies and the number of participating GS volunteers. The number of eligible GS participants invited to each study varies due to study criteria. All eligible GS participants consented to re-contact.

Study Eligibility Criteria:

[a] Known email or postal address.

[b] Participated in the baseline DOLORisk survey and consented to re-contact for the follow-up survey.

[c] Living in Scotland and had a valid CHI number.

[d] Indicated willingness to undergo face-to-face assessment in the remote STRADL questionnaire.

[e] Resident in Scotland, known email address or postal address.

[f] Living in rural Scotland.

[g] Aged 50+, no diagnosis of dementia or medical/psychiatric disorders.

[h] Aged 50+, living in Scotland.

Re-contact study		Study Dates	GS participants eligible for re-contact	GS Participants (% Responded)
DOLORisk	Baseline questionnaire	May 16-Dec 16	20,221[a]	7,238 (35.8%)
	Follow up survey	Jun 18-Jun 19	6,657[b]	5,292 (79.5%)
STRADL	Remote questionnaire	2015-2017	21,525[c]	9,905 (46.0%)
	Face-to-face clinic visit		9,618[d]	1,189 (12.4%)
COVIDLife	COVIDLife1 survey	Apr 20-Jun 21	22,796[e]	4,968 (21.8%)
	Rural COVIDLife survey	Oct 20-Nov 20	1,559[f]	712 (45.7%)
EPAD	Alzheimer's dementia	2016-2018	3,779[g]	53 (1.4%)
HAGIS	COVID impact survey	2021-2022	14,891[h]	2,826 (19.0%)

For peer review only

Figure 1: 1A. Proportion of diabetes cases captured in each data source (total cases N=1,861). 1B. Venn diagram of the concordance of cases among data sources (N=1,852, excluding routine laboratory testing data for glycated haemoglobin (HbA1c) and deaths data).

SCI-DC - Scottish Care Information - Diabetes Collaboration.
PIS - Prescribing Information System.
SMR01 - Scottish Morbidity Records: General/Acute Inpatient and Day Case.
HbA1c - Routine laboratory testing data for haemoglobin A1c (level above 6.5%).
Self-report- prevalence reported by participant at baseline.
Deaths data – National records of Scotland deaths data.

Figure 2: Yearly number of access requests and publications using GS data over time.

For peer review only

Appendices

Appendix A. Full list of available linked NHS datasets and descriptions.

Scottish National healthcare data is collected as a series of Scottish Morbidity Records (SMR).

Linked Dataset	Coding	Date Range	N	Description
**Scottish Morbidity Records (SMR)**				
Outpatient Attendance (SMR00)[a]	None	1996-2020	21,270	Outpatient attendance information excluding A&E and genitourinary medicine.
General/Acute Inpatient and Day Case (SMR01)[a]	ICD9/ICD10	1980-2022	19,611	Acute inpatient and daycase hospital admissions: disease and procedure codes. Scotland-wide, around 750 000 admissions per annum since 1981.
Maternity Inpatient and Day Case (SMR02)[a]	ICD9/ICD10	1975-2020	8,238	Pregnancies and births: disease and procedure codes. Scotland-wide, around 58,000 deliveries per annum since 1975.
Mental Health Inpatient and Day Case (SMR04)[a]	ICD9/ICD10	1980-2020	578	Psychiatric hospital discharges and diagnostic information.
Scottish Cancer Registry (SMR06)[a]	ICD10	1996-2020	3,606	Personal, demographic, and diagnostic information on all new cases of cancer.
Scottish Birth Record (SMR11)[a]	ICD9/ICD10	1975-1992	3,246	Linked maternity, neonatal and stillbirth and infant death records from 1975, with records pertaining to mother and baby held together.
**Primary Care**				
General Practice (GP)	ReadV2	1980-2020	19,675	GP primary care attendances.
GP Out-of-Hours Data	ReadV2	2014-2022	8,533	Data on patients seen by GP Out of Hours (OOH) services across Scotland since 2014.
NHS24	Outcome Codes	2011-2022	12,326	Records of telehealth and telecare services provided through nhs24.scot website and NHS 24 111 phone service.
Accident and Emergency (A&E)	ICD10	2007-2020	15,248	Patient attendance information at Emergency Department (EDs), Minor Injuries Units (MIUs) and community hospital A&Es across NHS Scotland.
**Other Datasets**				
Routine laboratory testing	ReadV2	1997-2018	19,089	Laboratory test results from primary and secondary care provided by the Scottish Care Information (SCI) Store.
ICU Daily Data (SICSAG)	None	2019-2022	361	Scottish Intensive Care Society Audit Group (SICSAG) national database of patients admitted to adult Critical Care Units in Scotland since 1995.
ICU Episode Data (SICSAG)	None	2019-2022	361	
National Records of Scotland (NRS) Deaths Data	ICD10	2007-2022	1,659	All deaths occurring in Scotland. NRS Death Records are linked with NHS Scotland Scottish Morbidity Database.
Diabetes Registry (SCI-DC)	None	2019	1,423	The Scottish Care Information – Diabetes Collaboration (SCI-DC), established in 2002. Integrated electronic patient record for individuals with diabetes.
Pre-2009 Dispensing Data (PIS)	BNF	1989-2009	12,981	The Prescribing Information System (PIS) covers all NHS prescriptions prescribed, dispensed and reimbursed within the community setting. Pre-2009 data is poorly completed.
Post-2009 Dispensing Data (PIS)	BNF	2009-2020	21,486	
COVID-19 Vaccinations	None	2020-2022	19,128	Scottish Covid-19 vaccination data contains COVID-19 vaccination events since December 2020.

Testing (ECOSS)	None	2020-2022	16,537	Electronic Communication of Surveillance in Scotland (ECOSS) system surveillance data on COVID-19 infections reported from diagnostics and reference laboratories.
Dental (MIDAS)	SDR	1994-2019	19,870	Management Information & Dental Accounting System (MIDAS), the payment system for GDS dentists, information on NHS dentist appointments.
Scottish Drug Misuse Database	None	2001-2016	76	Scottish Drug Misuse Database (SDMD), attendances at broad range of services.

For peer review only

Appendix. B Secondary Care ICD-10 codes used to investigate cohort morbidities. Codes were derived from Gadd et al [43] **using CALIBER code lists. ICD-10 codes are used for all records after 1994 when they replaced the use of ICD-9 codes. Codes were used to query SMR01 (General/Acute Inpatient and Day Case), SMR06 (Scottish Cancer Registry) and NRS death registration datasets.**

**Secondary Care ICD-10 Codes**	
**Code**	**Description**
**Asthma**	
J45	Asthma
J46	Status asthmaticus
**Bowel cancer**	
C18	Malignant neoplasm of colon
**Breast cancer**	
C50	Malignant neoplasm of breast
**Chronic obstructive pulmonary disease**	
J40	Bronchitis, not specified as acute or chronic
J41	Simple and mucopurulent chronic bronchitis
J42	Unspecified chronic bronchitis
J43	Emphysema
J44	Other chronic obstructive pulmonary disease
**Dementia**	
F00	Dementia in Alzheimer’s disease
F01	Vascular dementia
F03	Unspecified dementia
F051	Delirium superimposed on dementia
G30	Alzheimer's disease
**Depression**	
F32	Major depressive disorder
F33	Major depressive disorder
**Diabetes**	
E10	Type 1 diabetes mellitus
E11	Type 2 diabetes mellitus
E12	Malnutrition-related diabetes mellitus
E13	Other specified diabetes mellitus
E14	Unspecified diabetes mellitus
H360	Diabetic retinopathy
O243	Diabetes mellitus in pregnancy: Pre-existing diabetes mellitus, unspecified
N083	Glomerular disorders in diabetes mellitus
H280	Diabetic cataract
O242	Diabetes mellitus in pregnancy: Pre-existing malnutrition-related diabetes mellitus
M142	Diabetic arthropathy
G632	Diabetic polyneuropathy
O240	Diabetes mellitus in pregnancy: Pre-existing diabetes mellitus, insulin-dependent
O241	Diabetes mellitus in pregnancy: Pre-existing diabetes mellitus, non-insulin-dependent
G590	Diabetic mononeuropathy
**Hypertension**	
I10	Essential (primary) hypertension

I11	Hypertensive heart disease
I12	Hypertensive renal disease
I13	Hypertensive heart and renal disease
I15	Secondary hypertension
**Ischaemic heart disease**	
I20	Angina pectoris
I21	Acute myocardial infarction
I22	Subsequent myocardial infarction
I23	Certain current complications following acute myocardial infarction
I24	Other acute ischaemic heart diseases
I25	Chronic ischaemic heart disease
**Lung Cancer**	
C34	Malignant neoplasm of bronchus and lung
**Osteoarthritis**	
M15	Polyarthrosis
M16	Coxarthrosis [arthrosis of hip]
M17	Gonarthrosis [arthrosis of knee]
M18	Arthrosis of first carpometacarpal joint
M19	Other arthrosis
**Prostate cancer**	
C61	Malignant neoplasm of prostate
**Rheumatoid arthritis**	
M05	Seropositive rheumatoid arthritis
M06	Other rheumatoid arthritis
J99.0	Rheumatoid lung disease
**Stroke**	
I63	Cerebral infarction
I64	Stroke, not specified as haemorrhage or infarction
G46.7	Other lacunar syndromes
G46.6	Pure sensory lacunar syndrome
G46.5	Pure motor lacunar syndrome
G46.3	Brain stem stroke syndrome
G46.4	Cerebellar stroke syndrome
I69.4	Sequelae of stroke, not specified as haemorrhage or infarction
G46.8	Other vascular syndromes of brain in cerebrovascular diseases
**COVID-19**	
U071	COVID-19, virus identified

Appendix C. Secondary Care ICD-9 Codes used to investigate cohort morbidities. ICD-9 codes were used for records up to 1994 when they were replaced with ICD-10. Codes were used to query SMR01 (General/Acute Inpatient and Day Case), SMR06 (Scottish Cancer Registry) and NRS death records datasets.

**Secondary Care ICD-9 Codes**	
**Code**	**Description**
**Asthma**	
493	Asthma
**Bowel cancer**	
153	Malignant neoplasm of colon
154	Malignant neoplasm of rectum rectosigmoid junction and anus
**Breast cancer**	
174	Malignant neoplasm of female breast
**chronic obstructive pulmonary disease**	
490	Bronchitis, not specified as acute or chronic
491	Chronic bronchitis
492	Emphysema
496	Chronic airway obstruction, not elsewhere classified
**Dementia**	
290	Dementias
2912	Alcohol-induced persisting dementia
2941	Dementia in conditions classified elsewhere
331	Other cerebral degenerations
**Depression**	
2962	Major depressive disorder single episode
2963	Major depressive disorder recurrent episode
311	Depressive disorder not elsewhere classified
**Diabetes**	
250	Diabetes mellitus
249	Secondary diabetes mellitus
**Hypertension**	
401	Essential hypertension
402	Hypertensive heart disease
403	Hypertensive renal disease
404	Hypertensive heart and renal disease
405	Secondary hypertension
**Ischaemic heart disease**	
410	Acute myocardial infarction
411	Other acute and subacute forms of ischemic heart disease
412	Old myocardial infarction
413	Angina pectoris
414	Other forms of chronic ischemic heart disease
**Lung cancer**	
162	Malignant neoplasm of trachea bronchus and lung
**Osteoarthritis**	
715	Osteoarthrosis and allied disorders
**Prostate cancer**	

185	Malignant neoplasm of prostate
**Rheumatoid arthritis**	
714	Rheumatoid arthritis and other inflammatory polyarthropathies
**Stroke**	
430	Subarachnoid haemorrhage
431	Intracerebral haemorrhage
433	Occlusion and stenosis of precerebral arteries
434	Occlusion of cerebral arteries

For peer review only

Appendix D. Primary Care ReadCode v2 Codes used to investigate cohort morbidities. Codes were derived from Gadd et al [43] using CALIBER code lists.

**Primary Care ReadCode v2 Codes**	
**Code**	**Description**
**Asthma**	
14B4.	H/O: asthma
173A.	Exercise induced asthma
173c.	Occupational asthma
173d.	Work aggravated asthma
1780	Aspirin induced asthma
1O2..	Asthma confirmed
21262	Asthma resolved
212G.	Asthma resolved
H3120	Chronic asthmatic bronchitis
H33..	Asthma
H330.	Extrinsic (atopic) asthma
H3300	Extrinsic asthma without status asthmaticus
H3301	Extrinsic asthma with status asthmaticus
H330z	Extrinsic asthma NOS
H331.	Intrinsic asthma
H3310	Intrinsic asthma without status asthmaticus
H3311	Intrinsic asthma with status asthmaticus
H331z	Intrinsic asthma NOS
H332.	Mixed asthma
H333.	Acute exacerbation of asthma
H334.	Brittle asthma
H335.	Chronic asthma with fixed airflow obstruction
H33z.	Asthma unspecified
H33z0	Status asthmaticus NOS
H33z1	Asthma attack
H33z2	Late-onset asthma
H33zz	Asthma NOS
**Bowel cancer**	
B13..	Malignant neoplasm of colon
B130.	Malignant neoplasm of hepatic flexure of colon
B131.	Malignant neoplasm of transverse colon
B132.	Malignant neoplasm of descending colon
B133.	Malignant neoplasm of sigmoid colon
B134.	Malignant neoplasm of caecum
B135.	Malignant neoplasm of appendix
B136.	Malignant neoplasm of ascending colon
B137.	Malignant neoplasm of splenic flexure of colon
B139.	Hereditary nonpolyposis colon cancer
B13z.	Malignant neoplasm of colon NOS
B1z0.	Malignant neoplasm of intestinal tract, part unspecified
**Breast cancer**	
B3251	Malignant melanoma of breast

For peer review only

B3351	Malignant neoplasm of skin of chest, excluding breast
B3352	Malignant neoplasm of skin of breast
B34..	Malignant neoplasm of female breast
B3401	Malignant neoplasm of areola of female breast
B34y.	Malignant neoplasm of other site of female breast
B34yz	Malignant neoplasm of other site of female breast NOS
B34z.	Malignant neoplasm of female breast NOS
B35..	Malignant neoplasm of male breast
B58y0	Secondary malignant neoplasm of breast
B830.	Carcinoma in situ of breast
BB94.	[M]Juvenile breast carcinoma
Byu6.	[X]Malignant neoplasm of breast
ZV103	[V]Personal history of malignant neoplasm of breast
**Chronic obstructive pulmonary disease**	
14B3.	History of chronic obstructive pulmonary disease
H3...	Chronic obstructive pulmonary disease
H31..	Chronic bronchitis
H310.	Simple chronic bronchitis
H3100	Chronic catarrhal bronchitis
H310z	Simple chronic bronchitis NOS
H311.	Mucopurulent chronic bronchitis
H3110	Purulent chronic bronchitis
H3111	Fetid chronic bronchitis
H311z	Mucopurulent chronic bronchitis NOS
H312.	Obstructive chronic bronchitis
H3120	Chronic asthmatic bronchitis
H3121	Emphysematous bronchitis
H3122	Acute exacerbation of chronic obstructive airways disease
H3123	Bronchiolitis obliterans
H312z	Obstructive chronic bronchitis NOS
H313.	Mixed simple and mucopurulent chronic bronchitis
H31y.	Other chronic bronchitis
H31y1	Chronic tracheobronchitis
H31yz	Other chronic bronchitis NOS
H31z.	Chronic bronchitis NOS
H32..	Emphysema
H320.	Chronic bullous emphysema
H3200	Segmental bullous emphysema
H3201	Zonal bullous emphysema
H3202	Giant bullous emphysema
H3203	Bullous emphysema with collapse
H320z	Chronic bullous emphysema NOS
H321.	Panlobular emphysema
H322.	Centrilobular emphysema
H32y.	Other emphysema
H32y0	Acute vesicular emphysema
H32y1	Atrophic (senile) emphysema
H32y2	MacLeod's unilateral emphysema

For peer review only

H32yz	Other emphysema NOS
H32z.	Emphysema NOS
H36..	Mild chronic obstructive pulmonary disease
H37..	Moderate chronic obstructive pulmonary disease
H38..	Severe chronic obstructive pulmonary disease
H39..	Very severe chronic obstructive pulmonary disease
H3A..	End stage chronic obstructive airways disease
H3y..	Other specified chronic obstructive airways disease
H3y0.	Chronic obstruct pulmonary dis with acute lower respiratory infection
H3y1.	Chronic obstruct pulmonary dis with acute exacerbation, unspecified
H3z..	Chronic obstructive airways disease NOS
H4640	Chronic emphysema due to chemical fumes
H4641	Obliterative bronchiolitis due to chemical fumes
H5832	Eosinophilic bronchitis
Hyu30	[X]Other emphysema
Hyu31	[X]Other specified chronic obstructive pulmonary disease
**Dementia**	
1461	H/O: dementia
66h..	Dementia monitoring
6AB..	Dementia annual review
8CMZ.	Dementia care plan
9hD..	Exception reporting: dementia quality indicators
9hD0.	Excepted from dementia quality indicators: Patient unsuitable
9hD1.	Excepted from dementia quality indicators: Informed dissent
9Ou..	Dementia monitoring administration
9Ou1.	Dementia monitoring first letter
9Ou2.	Dementia monitoring second letter
9Ou3.	Dementia monitoring third letter
9Ou4.	Dementia monitoring verbal invite
9Ou5.	Dementia monitoring telephone invite
E00..	Senile and presenile organic psychotic conditions
E000.	Uncomplicated senile dementia
E001.	Presenile dementia
E0010	Uncomplicated presenile dementia
E0011	Presenile dementia with delirium
E0012	Presenile dementia with paranoia
E0013	Presenile dementia with depression
E001z	Presenile dementia NOS
E002.	Senile dementia with depressive or paranoid features
E0020	Senile dementia with paranoia
E0021	Senile dementia with depression
E002z	Senile dementia with depressive or paranoid features NOS
E003.	Senile dementia with delirium
E004.	Arteriosclerotic dementia
E0040	Uncomplicated arteriosclerotic dementia
E0041	Arteriosclerotic dementia with delirium
E0042	Arteriosclerotic dementia with paranoia

For peer review only

E0043	Arteriosclerotic dementia with depression
E004z	Arteriosclerotic dementia NOS
E00y.	Other senile and presenile organic psychoses
E00z.	Senile or presenile psychoses NOS
E041.	Dementia in conditions EC
Eu00.	[X]Dementia in Alzheimer's disease
Eu000	[X]Dementia in Alzheimer's disease with early onset
Eu001	[X]Dementia in Alzheimer's disease with late onset
Eu002	[X]Dementia in Alzheimer's dis, atypical or mixed type
Eu00z	[X]Dementia in Alzheimer's disease, unspecified
Eu01.	[X]Vascular dementia
Eu010	[X]Vascular dementia of acute onset
Eu011	[X]Multi-infarct dementia
Eu012	[X]Subcortical vascular dementia
Eu013	[X]Mixed cortical and subcortical vascular dementia
Eu01y	[X]Other vascular dementia
Eu01z	[X]Vascular dementia, unspecified
Eu02z	[X] Unspecified dementia
Eu041	[X]Delirium superimposed on dementia
F110.	Alzheimer's disease
F1100	Alzheimer's disease with early onset
F1101	Alzheimer's disease with late onset
Fyu30	[X]Other Alzheimer's disease
ZS7C5	Language disorder of dementia
**Depression**	
1465	H/O: depression
212S.	Depression resolved
8BK0.	Depression management programme
8CAa.	Patient given advice about management of depression
8HHq.	Referral for guided self-help for depression
9H90.	Depression annual review
9H91.	Depression medication review
9H92.	Depression interim review
9HA0.	On depression register
9k4..	Depression - enhanced services administration
9k40.	Depression - enhanced service completed
9kQ..	On full dose long term treatment depression - enh serv admin
9Ov..	Depression monitoring administration
9Ov0.	Depression monitoring first letter
9Ov1.	Depression monitoring second letter
9Ov2.	Depression monitoring third letter
9Ov3.	Depression monitoring verbal invite
9Ov4.	Depression monitoring telephone invite
E0013	Presenile dementia with depression
E0021	Senile dementia with depression
E0043	Arteriosclerotic dementia with depression
E112.	Single major depressive episode
E1120	Single major depressive episode, unspecified

For peer review only

E1121	Single major depressive episode, mild
E1122	Single major depressive episode, moderate
E1123	Single major depressive episode, severe, without psychosis
E1124	Single major depressive episode, severe, with psychosis
E1125	Single major depressive episode, partial or unspec remission
E1126	Single major depressive episode, in full remission
E112z	Single major depressive episode NOS
E113.	Recurrent major depressive episode
E1130	Recurrent major depressive episodes, unspecified
E1131	Recurrent major depressive episodes, mild
E1132	Recurrent major depressive episodes, moderate
E1133	Recurrent major depressive episodes, severe, no psychosis
E1134	Recurrent major depressive episodes, severe, with psychosis
E1135	Recurrent major depressive episodes,partial/unspec remission
E1136	Recurrent major depressive episodes, in full remission
E1137	Recurrent depression
E113z	Recurrent major depressive episode NOS
E118.	Seasonal affective disorder
E11y2	Atypical depressive disorder
E11z2	Masked depression
E130.	Reactive depressive psychosis
E135.	Agitated depression
E2003	Anxiety with depression
E291.	Prolonged depressive reaction
E2B..	Depressive disorder NEC
E2B1.	Chronic depression
Eu204	[X]Post-schizophrenic depression
Eu251	[X]Schizoaffective disorder, depressive type
Eu32.	[X]Depressive episode
Eu320	[X]Mild depressive episode
Eu321	[X]Moderate depressive episode
Eu322	[X]Severe depressive episode without psychotic symptoms
Eu323	[X]Severe depressive episode with psychotic symptoms
Eu324	[X]Mild depression
Eu325	[X]Major depression, mild
Eu326	[X]Major depression, moderately severe
Eu327	[X]Major depression, severe without psychotic symptoms
Eu328	[X]Major depression, severe with psychotic symptoms
Eu329	[X]Single major depr ep, severe with psych, psych in remiss
Eu32A	[X]Recurr major depr ep, severe with psych, psych in remiss
Eu32y	[X]Other depressive episodes
Eu32z	[X]Depressive episode, unspecified
Eu33.	[X]Recurrent depressive disorder
Eu330	[X]Recurrent depressive disorder, current episode mild
Eu331	[X]Recurrent depressive disorder, current episode moderate
Eu332	[X]Recurr depress disorder cur epi severe without psyc sympt
Eu333	[X]Recurrent depress disorder cur epi severe with psyc symp
Eu334	[X]Recurrent depressive disorder, currently in remission

For peer review only

Eu33y	[X]Other recurrent depressive disorders
Eu33z	[X]Recurrent depressive disorder, unspecified
Eu341	[X]Dysthymia
Eu412	[X]Mixed anxiety and depressive disorder
**Diabetes**	
C108.	Type 1 diabetes mellitus
C1080	Type 1 diabetes mellitus with renal complications
C1082	Type 1 diabetes mellitus with neurological complications
C1084	Unstable type 1 diabetes mellitus
C1085	Type 1 diabetes mellitus with ulcer
C1087	Type 1 diabetes mellitus with retinopathy
C1088	Type 1 diabetes mellitus - poor control
C1089	Type 1 diabetes mellitus maturity onset
C108A	Type 1 diabetes mellitus without complication
C108D	Type 1 diabetes mellitus with nephropathy
C108E	Type 1 diabetes mellitus with hypoglycaemic coma
C108F	Type 1 diabetes mellitus with diabetic cataract
C108H	Type 1 diabetes mellitus with arthropathy
C108J	Type 1 diabetes mellitus with neuropathic arthropathy
C109.	Type 2 diabetes mellitus
C1090	Type 2 diabetes mellitus with renal complications
C1091	Type 2 diabetes mellitus with ophthalmic complications
C1092	Type 2 diabetes mellitus with neurological complications
C1094	Type 2 diabetes mellitus with ulcer
C1095	Type 2 diabetes mellitus with gangrene
C1096	Type 2 diabetes mellitus with retinopathy
C1097	Type 2 diabetes mellitus - poor control
C109A	Type 2 diabetes mellitus with mononeuropathy
C109B	Type 2 diabetes mellitus with polyneuropathy
C109C	Type 2 diabetes mellitus with nephropathy
C109D	Type 2 diabetes mellitus with hypoglycaemic coma
C109E	Type 2 diabetes mellitus with diabetic cataract
C109F	Type 2 diabetes mellitus with peripheral angiopathy
C109G	Type 2 diabetes mellitus with arthropathy
C109H	Type 2 diabetes mellitus with neuropathic arthropathy
C109J	Insulin treated Type 2 diabetes mellitus
C109K	Hyperosmolar non-ketotic state in type 2 diabetes mellitus
C10E.	Type 1 diabetes mellitus
C10E0	Type 1 diabetes mellitus with renal complications
C10E1	Type 1 diabetes mellitus with ophthalmic complications
C10E2	Type 1 diabetes mellitus with neurological complications
C10E3	Type 1 diabetes mellitus with multiple complications
C10E4	Unstable type 1 diabetes mellitus
C10E5	Type 1 diabetes mellitus with ulcer
C10E6	Type 1 diabetes mellitus with gangrene
C10E7	Type 1 diabetes mellitus with retinopathy
C10E8	Type 1 diabetes mellitus - poor control
C10E9	Type 1 diabetes mellitus maturity onset

C10EA	Type 1 diabetes mellitus without complication
C10EB	Type 1 diabetes mellitus with mononeuropathy
C10EC	Type 1 diabetes mellitus with polyneuropathy
C10ED	Type 1 diabetes mellitus with nephropathy
C10EE	Type 1 diabetes mellitus with hypoglycaemic coma
C10EF	Type 1 diabetes mellitus with diabetic cataract
C10EG	Type 1 diabetes mellitus with peripheral angiopathy
C10EH	Type 1 diabetes mellitus with arthropathy
C10EJ	Type 1 diabetes mellitus with neuropathic arthropathy
C10EK	Type 1 diabetes mellitus with persistent proteinuria
C10EL	Type 1 diabetes mellitus with persistent microalbuminuria
C10EM	Type 1 diabetes mellitus with ketoacidosis
C10EN	Type 1 diabetes mellitus with ketoacidotic coma
C10EP	Type 1 diabetes mellitus with exudative maculopathy
C10EQ	Type 1 diabetes mellitus with gastroparesis
C10F.	Type 2 diabetes mellitus
C10F0	Type 2 diabetes mellitus with renal complications
C10F1	Type 2 diabetes mellitus with ophthalmic complications
C10F2	Type 2 diabetes mellitus with neurological complications
C10F3	Type 2 diabetes mellitus with multiple complications
C10F4	Type 2 diabetes mellitus with ulcer
C10F5	Type 2 diabetes mellitus with gangrene
C10F6	Type 2 diabetes mellitus with retinopathy
C10F7	Type 2 diabetes mellitus - poor control
C10F9	Type 2 diabetes mellitus without complication
C10FA	Type 2 diabetes mellitus with mononeuropathy
C10FB	Type 2 diabetes mellitus with polyneuropathy
C10FC	Type 2 diabetes mellitus with nephropathy
C10FD	Type 2 diabetes mellitus with hypoglycaemic coma
C10FE	Type 2 diabetes mellitus with diabetic cataract
C10FF	Type 2 diabetes mellitus with peripheral angiopathy
C10FG	Type 2 diabetes mellitus with arthropathy
C10FH	Type 2 diabetes mellitus with neuropathic arthropathy
C10FJ	Insulin treated Type 2 diabetes mellitus
C10FK	Hyperosmolar non-ketotic state in type 2 diabetes mellitus
C10FL	Type 2 diabetes mellitus with persistent proteinuria
C10FM	Type 2 diabetes mellitus with persistent microalbuminuria
C10FN	Type 2 diabetes mellitus with ketoacidosis
C10FP	Type 2 diabetes mellitus with ketoacidotic coma
C10FQ	Type 2 diabetes mellitus with exudative maculopathy
C10FR	Type 2 diabetes mellitus with gastroparesis
**Hypertension**	
14A2.	H/O: hypertension
21261	Hypertension resolved
212K.	Hypertension resolved
61462	Hypertension induced by oral contraceptive pill
6624	Borderline hypertension: yearly observation
6627	Good hypertension control

For peer review only

6628	Poor hypertension control
662b.	Moderate hypertension control
662c.	Hypertension six month review
662d.	Hypertension annual review
662F.	Hypertension treatment. started
662G.	Hypertensive treatment changed
662O.	On treatment for hypertension
662r.	Trial withdrawal of antihypertensive therapy
7Q01.	High cost hypertension drugs
8B26.	Antihypertensive therapy
8BL0.	Patient on maximal tolerated antihypertensive therapy
8I3N.	Hypertension treatment refused
9OI9.	Hypertension monitor deleted
F4042	Blind hypertensive eye
F4213	Hypertensive retinopathy
G2...	Hypertensive disease
G20..	Essential hypertension
G200.	Malignant essential hypertension
G201.	Benign essential hypertension
G202.	Systolic hypertension
G203.	Diastolic hypertension
G20z.	Essential hypertension NOS
G21..	Hypertensive heart disease
G210.	Malignant hypertensive heart disease
G2100	Malignant hypertensive heart disease without CCF
G2101	Malignant hypertensive heart disease with CCF
G211.	Benign hypertensive heart disease
G2110	Benign hypertensive heart disease without CCF
G2111	Benign hypertensive heart disease with CCF
G21z.	Hypertensive heart disease NOS
G21z0	Hypertensive heart disease NOS without CCF
G21z1	Hypertensive heart disease NOS with CCF
G21zz	Hypertensive heart disease NOS
G22..	Hypertensive renal disease
G220.	Malignant hypertensive renal disease
G221.	Benign hypertensive renal disease
G222.	Hypertensive renal disease with renal failure
G22z.	Hypertensive renal disease NOS
G23..	Hypertensive heart and renal disease
G230.	Malignant hypertensive heart and renal disease
G231.	Benign hypertensive heart and renal disease
G232.	Hypertensive heart&renal dis wth (congestive) heart failure
G233.	Hypertensive heart and renal disease with renal failure
G234.	Hyperten heart&renal dis+both(congestv)heart and renal fail
G23z.	Hypertensive heart and renal disease NOS
G24..	Secondary hypertension
G240.	Secondary malignant hypertension
G2400	Secondary malignant renovascular hypertension

For peer review only

G240z	Secondary malignant hypertension NOS
G241.	Secondary benign hypertension
G2410	Secondary benign renovascular hypertension
G241z	Secondary benign hypertension NOS
G244.	Hypertension secondary to endocrine disorders
G24z.	Secondary hypertension NOS
G24z0	Secondary renovascular hypertension NOS
G24z1	Hypertension secondary to drug
G24zz	Secondary hypertension NOS
G2y..	Other specified hypertensive disease
G2z..	Hypertensive disease NOS
G672.	Hypertensive encephalopathy
Gyu2.	[X]Hypertensive diseases
Gyu21	[X]Hypertension secondary to other renal disorders
L122.	Other pre-existing hypertension in preg/childbirth/puerp
L1220	Other pre-existing hypertension in preg/childb/puerp unspec
L1221	Other pre-existing hypertension in preg/childb/puerp - deliv
L1223	Other pre-exist hypertension in preg/childb/puerp-not deliv
L122z	Other pre-existing hypertension in preg/childb/puerp NOS
L127.	Pre-eclampsia or eclampsia with pre-existing hypertension
L127z	Pre-eclampsia or eclampsia + pre-existing hypertension NOS
L128.	Pre-exist hypertension compl preg childbirth and puerperium
L1280	Pre-exist hyperten heart dis compl preg childbth+puerperium
L1282	Pre-exist 2ndry hypertens comp preg childbth and puerperium
TJC7.	Adverse reaction to other antihypertensives
TJC7z	Adverse reaction to antihypertensives NOS
U60C5	[X]Oth antihyperten drug caus advers eff in therap use, NEC
**Ischaemic heart disease**	
G3...	Ischaemic heart disease
G30..	Acute myocardial infarction
G300.	Acute anterolateral infarction
G301.	Other specified anterior myocardial infarction
G301z	Anterior myocardial infarction NOS
G302.	Acute inferolateral infarction
G303.	Acute inferoposterior infarction
G304.	Posterior myocardial infarction NOS
G305.	Lateral myocardial infarction NOS
G307.	Acute subendocardial infarction
G3070	Acute non-Q wave infarction
G3071	Acute non-ST segment elevation myocardial infarction
G308.	Inferior myocardial infarction NOS
G309.	Acute Q-wave infarct
G30A.	Mural thrombosis
G30X0	Acute ST segment elevation myocardial infarction
G30yz	Other acute myocardial infarction NOS
G30z.	Acute myocardial infarction NOS
G31..	Other acute and subacute ischaemic heart disease
G310.	Postmyocardial infarction syndrome

For peer review only

G311.	Preinfarction syndrome
G3110	Myocardial infarction aborted
G3111	Unstable angina
G3112	Angina at rest
G3114	Worsening angina
G3115	Acute coronary syndrome
G31y0	Acute coronary insufficiency
G31yz	Other acute and subacute ischaemic heart disease NOS
G32..	Old myocardial infarction
G33..	Angina pectoris
G3300	Nocturnal angina
G330z	Angina decubitus NOS
G331.	Prinzmetal's angina
G332.	Coronary artery spasm
G33z.	Angina pectoris NOS
G33z3	Angina on effort
G33z4	Ischaemic chest pain
G33z6	New onset angina
G33z7	Stable angina
G33zz	Angina pectoris NOS
G340.	Coronary atherosclerosis
G3400	Single coronary vessel disease
G3401	Double coronary vessel disease
G341.	Aneurysm of heart
G3410	Ventricular cardiac aneurysm
G342.	Atherosclerotic cardiovascular disease
G343.	Ischaemic cardiomyopathy
G34z.	Other chronic ischaemic heart disease NOS
G34z0	Asymptomatic coronary heart disease
G35..	Subsequent myocardial infarction
G366.	Thrombosis of atrium, auricular appendage, and ventricle as current complications following acute myocardial infarction
G37..	Cardiac syndrome X
G38..	Postoperative myocardial infarction
G39..	Coronary microvascular disease
G3z..	Ischaemic heart disease NOS
**Lung cancer**	
B22..	Malignant neoplasm of trachea, bronchus and lung
B2211	Malignant neoplasm of hilus of lung
B222.	Malignant neoplasm of upper lobe, bronchus or lung
B2221	Malignant neoplasm of upper lobe of lung
B2231	Malignant neoplasm of middle lobe of lung
B224.	Malignant neoplasm of lower lobe, bronchus or lung
B2241	Malignant neoplasm of lower lobe of lung
B22z.	Malignant neoplasm of bronchus or lung NOS
B570.	Secondary malignant neoplasm of lung
**Osteoarthritis**	
14G2.	H/O: osteoarthritis

For peer review only

2G26.	O/E - hands - Heberden's nodes
7P204	Delivery of rehabilitation for osteoarthritis
N05..	Osteoarthritis and allied disorders
N050.	Generalised osteoarthritis - OA
N0500	Generalised osteoarthritis of unspecified site
N0501	Generalised osteoarthritis of the hand
N0502	Generalised osteoarthritis of multiple sites
N0503	Bouchard's nodes with arthropathy
N0504	Primary generalized osteoarthrosis
N0505	Secondary multiple arthrosis
N0506	Erosive osteoarthrosis
N0507	Heberden's nodes with arthropathy
N050z	Generalised osteoarthritis NOS
N051.	Localised, primary osteoarthritis
N0510	Localised, primary osteoarthritis of unspecified site
N0511	Localised, primary osteoarthritis of the shoulder region
N0512	Localised, primary osteoarthritis of the upper arm
N0513	Localised, primary osteoarthritis of the forearm
N0514	Localised, primary osteoarthritis of the hand
N0515	Localised, primary osteoarthritis of the pelvic region/thigh
N0516	Localised, primary osteoarthritis of the lower leg
N0517	Localised, primary osteoarthritis of the ankle and foot
N0518	Localised, primary osteoarthritis of other specified site
N0519	Primary coxarthrosis, bilateral
N051A	Coxarthrosis resulting from dysplasia, bilateral
N051B	Primary gonarthrosis, bilateral
N051C	Primary arthrosis of first carpometacarpal joints, bilateral
N051D	Localised, primary osteoarthritis of the wrist
N051E	Localised, primary osteoarthritis of toe
N051F	Localised, primary osteoarthritis of elbow
N051G	Osteoarthritis of spinal facet joint
N051z	Localised, primary osteoarthritis NOS
N052.	Localised, secondary osteoarthritis
N0520	Localised, secondary osteoarthritis of unspecified site
N0521	Localised, secondary osteoarthritis of the shoulder region
N0522	Localised, secondary osteoarthritis of the upper arm
N0523	Localised, secondary osteoarthritis of the forearm
N0524	Localised, secondary osteoarthritis of the hand
N0525	Localised, secondary osteoarthritis of pelvic region/thigh
N0526	Localised, secondary osteoarthritis of the lower leg
N0527	Localised, secondary osteoarthritis of the ankle and foot
N0528	Localised, secondary osteoarthritis of other specified site
N052z	Localised, secondary osteoarthritis NOS
N053.	Localised osteoarthritis, unspecified
N0530	Localised osteoarthritis, unspecified, of unspecified site
N0531	Localised osteoarthritis, unspecified, of shoulder region
N0532	Localised osteoarthritis, unspecified, of the upper arm
N0533	Localised osteoarthritis, unspecified, of the forearm

For peer review only

N0534	Localised osteoarthritis, unspecified, of the hand
N0535	Localised osteoarthritis, unspecified, pelvic region/thigh
N0536	Localised osteoarthritis, unspecified, of the lower leg
N0537	Localised osteoarthritis, unspecified, of the ankle and foot
N0538	Localised osteoarthritis, unspecified, of other spec site
N0539	Arthrosis of first carpometacarpal joint, unspecified
N053z	Localised osteoarthritis, unspecified, NOS
N054.	Oligoarticular osteoarthritis, unspecified
N0540	Oligoarticular osteoarthritis, unspec, of unspecified sites
N0541	Oligoarticular osteoarthritis, unspecified, of shoulder
N0542	Oligoarticular osteoarthritis, unspecified, of upper arm
N0544	Oligoarticular osteoarthritis, unspecified, of hand
N0545	Oligoarticular osteoarthritis, unspecified, of pelvis/thigh
N0546	Oligoarticular osteoarthritis, unspecified, of lower leg
N0547	Oligoarticular osteoarthritis, unspecified, of ankle/foot
N0548	Oligoarticular osteoarthritis, unspecified, other spec sites
N0549	Oligoarticular osteoarthritis, unspecified, multiple sites
N054z	Osteoarthritis of more than one site, unspecified, NOS
N05z.	Osteoarthritis NOS
N05z0	Osteoarthritis NOS, of unspecified site
N05z1	Osteoarthritis NOS, of shoulder region
N05z4	Osteoarthritis NOS, of the hand
N05z5	Osteoarthritis NOS, pelvic region/thigh
N05z6	Osteoarthritis NOS, of the lower leg
N05z7	Osteoarthritis NOS, of ankle and foot
N05z8	Osteoarthritis NOS, other specified site
N05z9	Osteoarthritis NOS, of shoulder
N05zA	Osteoarthritis NOS, of sternoclavicular joint
N05zB	Osteoarthritis NOS, of acromioclavicular joint
N05zC	Osteoarthritis NOS, of elbow
N05zD	Osteoarthritis NOS, of distal radio-ulnar joint
N05zE	Osteoarthritis NOS, of wrist
N05zF	Osteoarthritis NOS, of MCP joint
N05zG	Osteoarthritis NOS, of PIP joint of finger
N05zH	Osteoarthritis NOS, of DIP joint of finger
N05zJ	Osteoarthritis NOS, of hip
N05zK	Osteoarthritis NOS, of sacro-iliac joint
N05zL	Osteoarthritis NOS, of knee
N05zM	Osteoarthritis NOS, of tibio-fibular joint
N05zN	Osteoarthritis NOS, of ankle
N05zP	Osteoarthritis NOS, of subtalar joint
N05zQ	Osteoarthritis NOS, of talonavicular joint
N05zR	Osteoarthritis NOS, of other tarsal joint
N05zS	Osteoarthritis NOS, of 1st MTP joint
N05zT	Osteoarthritis NOS, of lesser MTP joint
N05zU	Osteoarthritis NOS, of IP joint of toe
N05zz	Osteoarthritis NOS
Nyu2.	[X]Arthrosis

For peer review only

Nyu20	[X]Other polyarthrosis
Nyu21	[X]Other primary coxarthrosis
Nyu22	[X]Other dysplastic coxarthrosis
Nyu24	[X]Other secondary coxarthrosis, bilateral
Nyu25	[X]Other primary gonarthrosis
Nyu27	[X]Other secondary gonarthrosis, bilateral
Nyu28	[X]Other secondary gonarthrosis
Nyu29	[X]Other primary arthrosis of first carpometacarpal joint
Nyu2D	[X]Other specified arthrosis
Nyu2E	[X]Other secondary coxarthrosis
**Prostate cancer**	
7B365	Radical prostatectomy without pelvic node excision
4M01.	Gleason prostate grade 5-7 (medium)
4M02.	Gleason prostate grade 8-10 (high)
4M00.	Gleason prostate grade 2-4 (low)
B46..	Malignant neoplasm of prostate
7B360	Radical prostatectomy - unspecified excision of pelvic nodes
ZV104	[V]Personal history of malignant neoplasm of prostate
7B367	Radical prostatectomy with pelvic lymphadenectomy
4M0..	Gleason grading of prostate cancer
7B200	Radical cystoprostatourethrectomy
7B366	Radical prostatectomy with pelvic node sampling
14270	H/O: prostate cancer
7B202	Radical cystoprostatectomy
**Rheumatoid arthritis**	
14G1.	H/O: rheumatoid arthritis
2G25.	O/E - hands - ulnar deviation
2G27.	O/E-hands-rheumatoid spindling
66H..	Rheumatoid arthrit. monitoring
7P203	Delivery of rehabilitation for rheumatoid arthritis
9mM..	Rheumatoid arthritis monitoring invitation
9mM0.	Rheumatoid arthritis monitoring invitation first letter
9mM1.	Rheumatoid arthritis monitoring invitation second letter
9mM2.	Rheumatoid arthritis monitoring invitation third letter
9mM3.	Rheumatoid arthritis monitoring verbal invitation
9mM4.	Rheumatoid arthritis monitoring telephone invitation
F3712	Polyneuropathy in rheumatoid arthritis
F3964	Myopathy due to rheumatoid arthritis
G5y8.	Rheumatoid myocarditis
G5yA.	Rheumatoid carditis
H570.	Rheumatoid lung
N005.	Adult Still's Disease
N04..	Rheumatoid arthritis and other inflammatory polyarthropathy
N040.	Rheumatoid arthritis
N0400	Rheumatoid arthritis of cervical spine
N0401	Other rheumatoid arthritis of spine
N0402	Rheumatoid arthritis of shoulder
N0403	Rheumatoid arthritis of sternoclavicular joint

For peer review only

N0404	Rheumatoid arthritis of acromioclavicular joint
N0405	Rheumatoid arthritis of elbow
N0406	Rheumatoid arthritis of distal radio-ulnar joint
N0407	Rheumatoid arthritis of wrist
N0408	Rheumatoid arthritis of MCP joint
N0409	Rheumatoid arthritis of PIP joint of finger
N040A	Rheumatoid arthritis of DIP joint of finger
N040B	Rheumatoid arthritis of hip
N040C	Rheumatoid arthritis of sacro-iliac joint
N040D	Rheumatoid arthritis of knee
N040E	Rheumatoid arthritis of tibio-fibular joint
N040F	Rheumatoid arthritis of ankle
N040G	Rheumatoid arthritis of subtalar joint
N040H	Rheumatoid arthritis of talonavicular joint
N040J	Rheumatoid arthritis of other tarsal joint
N040K	Rheumatoid arthritis of 1st MTP joint
N040L	Rheumatoid arthritis of lesser MTP joint
N040M	Rheumatoid arthritis of IP joint of toe
N040N	Rheumatoid vasculitis
N040P	Seronegative rheumatoid arthritis
N040Q	Rheumatoid bursitis
N040R	Rheumatoid nodule
N040S	Rheumatoid arthritis - multiple joint
N040T	Flare of rheumatoid arthritis
N041.	Felty's syndrome
N042.	Other rheumatoid arthropathy + visceral/systemic involvement
N0421	Rheumatoid lung disease
N0422	Rheumatoid nodule
N042z	Rheumatoid arthropathy + visceral/systemic involvement NOS
N047.	Seropositive errosive rheumatoid arthritis
N04X.	Seropositive rheumatoid arthritis
N04y0	Rheumatoid lung
N04y2	Adult-onset Still's disease
N3622	Swan-neck finger deformity
Nyu10	[X]Rheumatoid arthritis+involvement/other organs or systems
Nyu11	[X]Other seropositive rheumatoid arthritis
Nyu12	[X]Other specified rheumatoid arthritis
Nyu1G	[X]Seropositive rheumatoid arthritis unspecified
**Stroke**	
G6400	Cerebral infarction due to thrombosis of cerebral arteries
G6W..	Cereb infarct due unsp occlus/stenos precerebr arteries
Gyu63	[X]Cerebrl infarctn due/unspcf occlusn or sten/cerebrl artrs
G63y0	Cerebral infarct due to thrombosis of precerebral arteries
G683.	Sequelae of cerebral infarction
G64z3	Right sided cerebral infarction
Gyu6G	[X]Cereb infarct due unsp occlus/stenos precerebr arteries
G640.	Cerebral thrombosis
G64z.	Cerebral infarction NOS

For peer review only

G641.	Cerebral embolism
G63..	Infarction - precerebral
G64z0	Brainstem infarction
G6410	Cerebral infarction due to embolism of cerebral arteries
G64z4	Infarction of basal ganglia
G6X..	Cerebrl infarctn due/unspcf occlusn or sten/cerebrl artrs
G64z1	Wallenberg syndrome
G64..	Cerebral arterial occlusion
G63y1	Cerebral infarction due to embolism of precerebral arteries
G64z2	Left sided cerebral infarction
Gyu64	[X]Other cerebral infarction
14A7.	H/O: CVA/stroke
14AK.	H/O: Stroke in last year
1M4..	Central post-stroke pain
661M7	Stroke self-management plan agreed
661N7	Stroke self-management plan review
662e.	Stroke/CVA annual review
662M.	Stroke monitoring
662M1	Stroke 6 month review
662M2	Stroke initial post discharge review
7P242	Delivery of rehabilitation for stroke
8HHM.	Ref to multidisciplinary stroke function improvement service
8IEC.	Ref multidisciplinary stroke function improvement declined
9h2..	Exception reporting: stroke quality indicators
9h21.	Excepted from stroke quality indicators: Patient unsuitable
9h22.	Excepted from stroke quality indicators: Informed dissent
Fyu56	[X]Other lacunar syndromes
G66..	Stroke and cerebrovascular accident unspecified
G663.	Brain stem stroke syndrome
G664.	Cerebellar stroke syndrome
G665.	Pure motor lacunar syndrome
G666.	Pure sensory lacunar syndrome
G667.	Left sided CVA
G668.	Right sided CVA
G68X.	Sequelae of stroke, not specified as haemorrhage or infarction
Gyu6C	[X]Sequelae of stroke, not specified as haemorrhage or infarction
L440.	Stroke in the puerperium
ZV125	[V]Personal history of stroke
COVID-19	
1JX1.	Suspected disease caused by 2019-nCoV (novel coronavirus)
A7951	Disease caused by 2019-nCoV (novel coronavirus)
4J3R.	2019-nCoV (novel coronavirus) serology
4J3R1	2019-nCoV (novel coronavirus) detected

For peer review only

Appendix E. Summary of the top 50 most cited publications using GS data. A full list of publications can be found at https://www.ed.ac.uk/generation-scotland/what-found/publications.

First Author	Title	Year Published	Journal	Keywords	Citations
Wray	Genome-wide association analyses identify 44 risk variants and refine the genetic architecture of major depression.	2018	Nature Genetics	Cognition and Mental Health	2,243
Lee	Gene discovery and polygenic prediction from a genome-wide association study of educational attainment in 1.1 million individuals.	2018	Nature Genetics	Data	1,714
Howard	Genome-wide meta-analysis of depression identifies 102 independent variants and highlights the importance of the prefrontal brain regions.	2019	Nature Neuroscience	Cognition and Mental Health	1,483
Pardiñas	Common schizophrenia alleles are enriched in mutation-intolerant genes and in regions under strong background selection.	2018	Nature Genetics	Cognition and Mental Health	1,376
Okbay	Genome-wide association study identifies 74 loci associated with educational attainment.	2016	Nature	Cognition and Mental Health	1,292
Anttila	Analysis of shared heritability in common disorders of the brain.	2018	Science	Cognition and Mental Health	1,202
Pairo-Castineira	Genetic mechanisms of critical illness in Covid-19.	2020	Nature	COVID-19	1,162
UK10K Consortium	The UK10K project identifies rare variants in health and disease.	2015	Nature	Data	1,030
Rahbari	Timing, rates and spectra of human germline mutation.	2015	Nature Genetics	Data	859
Lazaridis	Genomic insights into the origin of farming in the ancient Near East.	2016	Nature	Data	850
Fitzgerald	Large-scale discovery of novel genetic causes of developmental disorders.	2014	Nature	Cognition and Mental Health	693
Repapi	Genome-wide association study identifies five loci associated with lung function.	2009	Nature Genetics	Respiratory Disease	646
Davies	Study of 300,486 individuals identifies 148 independent genetic loci influencing general cognitive function.	2018	Nature Communications	Cognition and Mental Health	551
Warren	Genome-wide association analysis identifies novel blood pressure loci and offers biological insights into cardiovascular risk.	2017	Nature genetics	Cardiovascular Disease	541
Roselli	Multi-Ethnic Genome-wide Association Study for Atrial Fibrillation.	2018	Nature Genetics	Cardiovascular	522
Grotzinger	Genomic structural equation modelling provides insights into the multivariate genetic architecture of complex traits.	2019	Nature Human Behaviour	Methodology	521

Liu	Biological and clinical insights from genomic analysis of plasma lipids in >300,000 individuals.	2017	Nature	Cardiovascular Disease	511
Wuttke	A catalogue of genetic loci associated with kidney function from analyses of a million individuals.	2019	Nature Genetics	Kidney Disease	509
Day	Genomic analyses identify hundreds of variants associated with age at menarche and support a role for puberty timing in cancer risk.	2017	Nature Genetics	Cancer	466
Kwong	Mental health before and during the COVID-19 pandemic in two longitudinal UK population cohorts.	2020	British Journal of Psychiatry	COVID-19	461
Soler Artigas	Genome-wide association and large-scale follow-up identifies 16 new loci influencing lung function.	2011	Nature Genetics	Respiratory Disease	458
Messner	Ultra-high-throughput clinical proteomics reveals classifiers of COVID-19 infection.	2020	Cell Systems	COVID-19	433
Bethlehem	Brain charts for the human lifespan.	2022	Nature	Brain Charts	416
Davies	Genetic contributions to variation in general cognitive function: a meta-analysis of genome-wide association studies in the CHARGE consortium (N=53,949).	2015	Molecular Psychiatry	Cognition and Mental Health	416
Davies	Genome-wide association study of cognitive functions and educational attainment in UK Biobank (N=112,151).	2016	Molecular Psychiatry	Cognition and Mental Health	370
Clarke	Genome-wide association study of alcohol consumption and genetic overlap with other health-related traits in UK Biobank (N=112,117).	2017	Molecular Psychiatry	Alcohol	368
Schmidt	PCSK9 genetic variants and risk of type 2 diabetes: a Mendelian randomisation study.	2016	The Lancet Diabetes & Endocrinology	Diabetes	366
Day	Large-scale genomic analyses link reproductive aging to hypothalamic signalling, breast cancer susceptibility and BRCA1-mediated DNA repair.	2015	Nature Genetics	Cancer	366
Shrine	New genetic signals for lung function highlight pathways and pleiotropy, and chronic obstructive pulmonary disease associations across multiple ancestries.	2019	Nature Genetics	Cardiovascular	332
Luciano	Association analysis in over 329,000 individuals identifies 116 independent variants influencing neuroticism.	2017	Nature Genetics	Cognition and Mental Health	312
Turcot	Protein-altering variants associated with body mass index implicate pathways that control energy intake and expenditure in obesity.	2017	Nature Genetics	Lifestyle	300

Rietveld	Common genetic variants associated with cognitive performance identified using the proxy-phenotype method.	2014	PNAS 2014	Cognition and Mental Health	296
Surendran	Trans-ancestry meta-analyses identify rare and common variants associated with blood pressure and hypertension.	2016	Nature Genetics	Cardiovascular Disease	294
de Moor	Meta-analysis of Genome-wide Association Studies for Neuroticism, and the Polygenic Association with Major Depressive Disorder.	2015	JAMA Psychiatry	Cognition and Mental Health	293
Christopherson	Large-scale analyses of common and rare variants identify 12 new loci associated with atrial fibrillation.	2017	Nature Genetics	Cardiovascular Disease	290
Wain	Genome-wide association analyses for lung function and chronic obstructive pulmonary disease identify new loci and potential druggable targets.	2017	Nature genetics	Respiratory Disease	283
Brown	Vascular Consequences of Pre-eclampsia.	2015	Journal of Hypertension Volume	Cardiovascular Disease	283
Smith, D. J.	Genome-wide analysis of over 106,000 individuals identifies 9 neuroticism-associated loci.	2016	Molecular Psychiatry	Cognition and Mental Health	266
Graham	The power of genetic diversity in genome-wide association studies of lipids.	2021	Nature	GWAS	261
Chen	The Trans-Ancestral Genomic Architecture of Glycaemic Traits.	2021	Nature Genetics	Other	250
Tin	Target genes, variants, tissues and transcriptional pathways influencing human serum urate levels.	2019	Nature Genetics	Kidney Disease	250
Schumann	KLB is associated with alcohol drinking, and its gene product β-Klotho is necessary for FGF21 regulation of alcohol preference.	2016	PNAS	Alcohol	226
Abul-Husn	Personalized Medicine and the Power of Electronic Health Records.	2019	Cell	Ethical, Legal and Social Issues	218
Clarke	Common polygenic risk for autism spectrum disorder (ASD) is associated with cognitive ability in the general population.	2015	Molecular Psychiatry	Cognition and Mental Health	217
Wessel	Low-frequency and rare exome chip variants associate with fasting glucose and type 2 diabetes susceptibility.	2015	Nature Communications	Diabetes	215
Welsh	Cardiac Troponin T and Troponin I in the general population: contrasting genetic determinants and outcomes.	2019	Circulation	Cardiovascular	214
Graff	Genome-wide physical activity interactions in adiposity — a meta-analysis of 200,452 adults.	2017	PLoS Genetics	Obesity	200

Justice	Genome-Wide Meta-Analysis of 241,258 Adults Accounting for Smoking Behaviour Identifies Novel Loci for Obesity Traits.	2017	Nature Communications	Smoking, Obesity	200
Marioni	Molecular genetic contributions to socioeconomic status and intelligence.	2014	Intelligence	Cognition and Mental Health	196
Power	Genome-wide Association for Major Depression Through Age at Onset Stratification: Major Depressive Disorder Working Group of the Psychiatric Genomics Consortium.	2016	Biological Psychiatry	Cognition and Mental Health	193

For peer review only

Appendix F. List of current GS management and delivery team, investigators and members of the Scientific Steering Committee.

**GS Scientific Steering Committee**
Professor Dame Anna F Dominiczak (University of Glasgow & Chief Scientist Office, Scotland) – Chair<br>Professor Cathie Sudlow (University of Edinburgh) – GS Director<br>Professor Heather Whalley (University of Edinburgh) – GS Chief Scientist<br>Professor Julie Brittenden (University of Glasgow)<br>Dr Christian Cole (University of Dundee)<br>Professor Riccardo Marioni (University of Edinburgh)<br>Professor Zosia Miedzybrodzka (University of Aberdeen)<br>Professor Sandosh Padmanabhan (University of Glasgow)<br>Professor Blair Smith (University of Dundee)<br>Katie Wilde (University of Aberdeen)
**GS Scientific Leadership Team**
Professor Cathie Sudlow – GS Director and Principal Investigator (PI)<br>Professor David Porteous - Co-Investigator (Former Director and PI)<br>Professor Andrew McIntosh- Co-Investigator<br>Professor Riccardo Marioni- Co-Investigator<br>Professor Caroline Hayward- Co-Investigator<br>Professor Heather Whalley – GS Chief Scientist and Co-Investigator
**GS Management and Delivery Team**
Professor Cathie Sudlow (University of Edinburgh) – Director<br>Archie Campbell (University of Edinburgh) - Chief Technology Officer<br>Robin Flaig (University of Edinburgh) - Chief Operations Officer<br>Dr Daniel McCartney (University of Edinburgh) - Senior Bioinformatics Analyst<br>Professor Heather Whalley (University of Edinburgh) - Chief Scientist
Fiona Clark (University of Edinburgh) – Partnership Coordinator<br>Elly Darrah (University of Edinburgh) – Administrator<br>Liz Kirby (University of Edinburgh) - Research Support Officer<br>Hannah Milbourn (University of Edinburgh) - Health Data Scientist<br>Anne Richmond (University of Edinburgh) - Bioinformatics Analyst<br>Sarah Robertson (University of Edinburgh) - Young Person Engagement & Recruitment Co-ordinator<br>Rosie Tatham (University of Edinburgh) – Administrator<br>Alex Wood (University of Edinburgh) - Data Analyst / Developer<br>Dr Zhuoni Xiao (University of Edinburgh) – Research Fellow<br>Leah Young (University of Edinburgh) - Research Support Officer

For peer review only

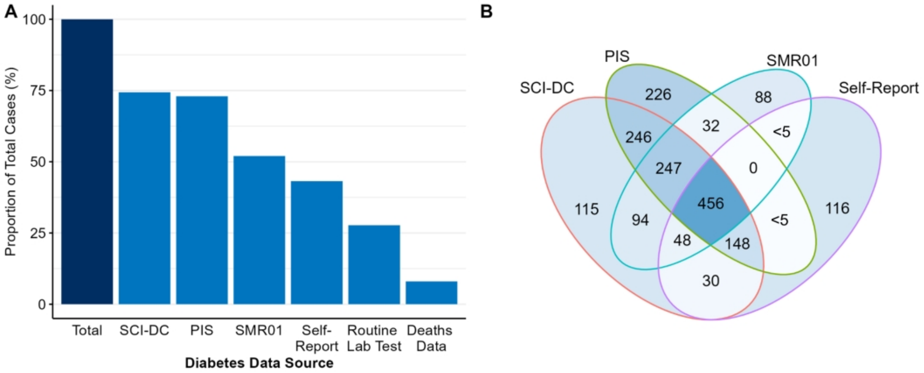

Figure 1: 1A. Proportion of diabetes cases captured in each data source (total cases N=1,861). 1B. Venn diagram of the concordance of cases among data sources (N=1,852, excluding routine laboratory testing data for glycated haemoglobin (HbA1c) and deaths data).
SCI-DC - Scottish Care Information - Diabetes Collaboration.
PIS - Prescribing Information System.
SMR01 - Scottish Morbidity Records: General/Acute Inpatient and Day Case.
HbA1c - Routine laboratory testing data for haemoglobin A1c (level above 6.5%).
Self-report- prevalence reported by participant at baseline.
Deaths data – National records of Scotland deaths data.

678x284mm (130 x 130 DPI)

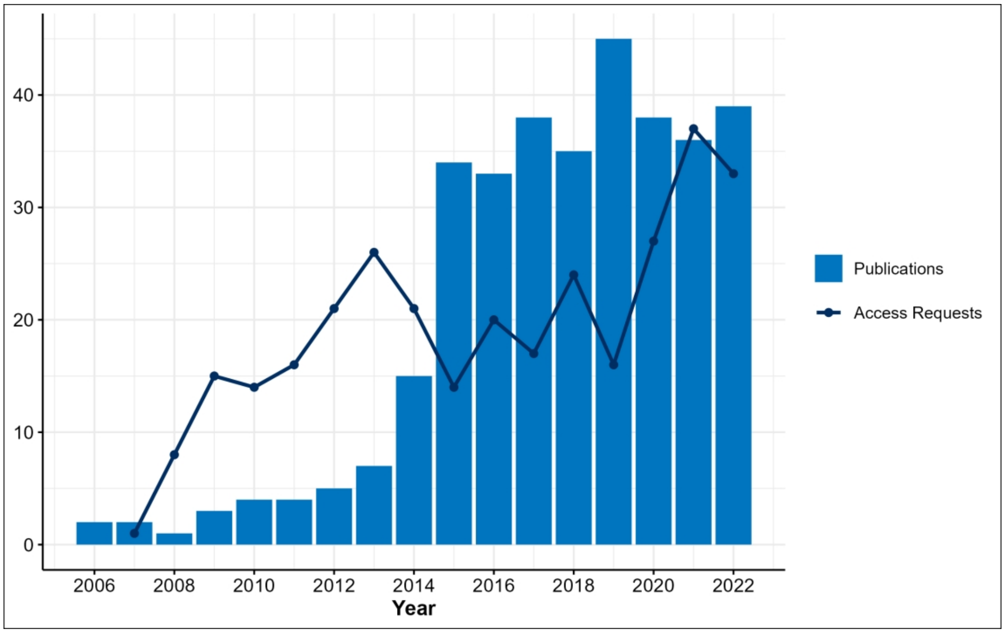

Figure 2: Yearly number of access requests and publications using GS data over time.

516x322mm (118 x 118 DPI)

BMJ Open

Cohort Profile: Generation Scotland – an update on Scotland's longitudinal family health study

Journal:	*BMJ Open*
Manuscript ID	bmjopen-2024-084719.R1
Article Type:	Cohort profile
Date Submitted by the Author:	21-May-2024
Complete List of Authors:	Milbourn, Hannah; The University of Edinburgh Centre for Genomic and Experimental Medicine, Institute of Genetics and Cancer<br>McCartney, Daniel; The University of Edinburgh Centre for Genomic and Experimental Medicine, Institute of Genetics and Cancer<br>Richmond, Anne; The University of Edinburgh Centre for Genomic and Experimental Medicine, Institute of Genetics and Cancer<br>Campbell, Archie; The University of Edinburgh Centre for Genomic and Experimental Medicine, Institute of Genetics and Cancer<br>Flaig, Robin; The University of Edinburgh Usher Institute of Population Health Sciences and Informatics, Centre for Medical Informatics<br>Robertson, Sarah; The University of Edinburgh Centre for Genomic and Experimental Medicine, Institute of Genetics and Cancer<br>Fawns-Ritchie, Chloe; University of Dundee Division of Psychology, School of Humanities, Social Sciences and Law; The University of Edinburgh Psychology Department<br>Hayward, Caroline; The University of Edinburgh Centre for Genomic and Experimental Medicine, Institute of Genetics and Cancer<br>Marioni, Riccardo; The University of Edinburgh Centre for Genomic and Experimental Medicine, Institute of Genetics and Cancer<br>McIntosh, Andrew; The University of Edinburgh Division of Psychiatry<br>Porteous, David; The University of Edinburgh Centre for Genomic and Experimental Medicine, Institute of Genetics and Cancer<br>Whalley, Heather; The University of Edinburgh Centre for Genomic and Experimental Medicine, Institute of Genetics and Cancer<br>Sudlow, Cathie; The University of Edinburgh Usher Institute of Population Health Sciences and Informatics
**Primary Subject Heading**:	Epidemiology
Secondary Subject Heading:	Genetics and genomics, Mental health
Keywords:	EPIDEMIOLOGY, GENETICS, Electronic Health Records

SCHOLARONE™
Manuscripts

I, the Submitting Author has the right to grant and does grant on behalf of all authors of the Work (as defined in the below author licence), an exclusive licence and/or a non-exclusive licence for contributions from authors who are: i) UK Crown employees; ii) where BMJ has agreed a CC-BY licence shall apply, and/or iii) in accordance with the terms applicable for US Federal Government officers or employees acting as part of their official duties; on a worldwide, perpetual, irrevocable, royalty-free basis to BMJ Publishing Group Ltd ("BMJ") its licensees and where the relevant Journal is co-owned by BMJ to the co-owners of the Journal, to publish the Work in this journal and any other BMJ products and to exploit all rights, as set out in our licence.

The Submitting Author accepts and understands that any supply made under these terms is made by BMJ to the Submitting Author unless you are acting as an employee on behalf of your employer or a postgraduate student of an affiliated institution which is paying any applicable article publishing charge ("APC") for Open Access articles. Where the Submitting Author wishes to make the Work available on an Open Access basis (and intends to pay the relevant APC), the terms of reuse of such Open Access shall be governed by a Creative Commons licence – details of these licences and which Creative Commons licence will apply to this Work are set out in our licence referred to above.

Other than as permitted in any relevant BMJ Author's Self Archiving Policies, I confirm this Work has not been accepted for publication elsewhere, is not being considered for publication elsewhere and does not duplicate material already published. I confirm all authors consent to publication of this Work and authorise the granting of this licence.

For peer review only

Cohort Profile: Generation Scotland – an update on Scotland's longitudinal family health study

Hannah Milbourn, Daniel L McCartney, Anne Richmond, Archie Campbell, Robin Flaig, Sarah Robertson, Chloe Fawns-Ritchie, Caroline Hayward, Riccardo E Marioni, Andrew M McIntosh, David J Porteous, Heather Whalley, Cathie Sudlow

Correspondence to Professor Heather Whalley: Heather.Whalley@ed.ac.uk
Centre for Genomic and Experimental Medicine, Institute of Genetics and Cancer, University of Edinburgh, Western General Hospital, Edinburgh, UK, EH4 2XU

Hannah Milbourn

Centre for Genomic and Experimental Medicine, Institute of Genetics and Cancer, University of Edinburgh, Western General Hospital, Edinburgh, UK, EH4 2XU

Hannah.Milbourn@ed.ac.uk

ORCID: 0000-0003-4479-8635

Dr Daniel L McCartney

Centre for Genomic and Experimental Medicine, Institute of Genetics and Cancer, University of Edinburgh, Western General Hospital, Edinburgh, UK, EH4 2XU

Daniel.McCartney@ed.ac.uk

ORCID: 0000-0003-3242-0360

Anne Richmond

Centre for Genomic and Experimental Medicine, Institute of Genetics and Cancer, University of Edinburgh, Western General Hospital, Edinburgh, UK, EH4 2XU

Anne.Richmond@ed.ac.uk

ORCID: 0000-0002-9504-510X

Archie Campbell

Centre for Genomic and Experimental Medicine, Institute of Genetics and Cancer, University of Edinburgh, Western General Hospital, Edinburgh, UK, EH4 2XU

Archie.Campbell@ed.ac.uk

ORCID: 0000-0003-0198-5078

Robin Flaig

For peer review only

Centre for Medical Informatics, Usher Institute, University of Edinburgh, Nine, Edinburgh Bioquarter, 9 Little France Road, Edinburgh, UK, EH16 4UX

Robin.Flaig@ed.ac.uk

ORCID: 0000-0001-8007-1279

Sarah Robertson

Centre for Genomic and Experimental Medicine, Institute of Genetics and Cancer, University of Edinburgh, Western General Hospital, Edinburgh, UK, EH4 2XU

Sarah.Robertson@ed.ac.uk

Dr Chloe Fawns-Ritchie

Division of Psychology, School of Humanities, Social Sciences and Law, University of Dundee, Scrymgeour Building, Dundee, UK, DD1 4HN

Department of Psychology, University of Edinburgh, 7 George Square, Edinburgh, UK, EH6 5PQ

cfawnsritchie001@dundee.ac.uk

ORCID: 0000-0002-7493-2228

Professor Caroline Hayward

Centre for Genomic and Experimental Medicine, Institute of Genetics and Cancer, University of Edinburgh, Western General Hospital, Edinburgh, UK, EH4 2XU

Caroline.Hayward@ed.ac.uk

ORCID: 0000-0002-9405-9550

Professor Riccardo E Marioni

Centre for Genomic and Experimental Medicine, Institute of Genetics and Cancer, University of Edinburgh, Western General Hospital, Edinburgh, UK, EH4 2XU

Riccardo.Marioni@ed.ac.uk

ORCID: 0000-0003-4430-4260

Professor Andrew M McIntosh

Division of Psychiatry, University of Edinburgh, Royal Edinburgh Hospital, Edinburgh, UK, EH10 5HF

Andrew.McIntosh@ed.ac.uk

ORCID: 0000-0002-0198-4588

Professor David J Porteous

For peer review only

Centre for Genomic and Experimental Medicine, Institute of Genetics and Cancer, University of Edinburgh, Western General Hospital, Edinburgh, UK, EH4 2XU

David.Porteous@ed.ac.uk

ORCID: 0000-0003-1249-6106

Professor Heather Whalley

Centre for Genomic and Experimental Medicine, Institute of Genetics and Cancer, University of Edinburgh, Western General Hospital, Edinburgh, UK, EH4 2XU

Heather.Whalley@ed.ac.uk

ORCID: 0000-0002-4505-8869

Professor Cathie Sudlow

Centre for Medical Informatics, Usher Institute, University of Edinburgh, Nine, Edinburgh Bioquarter, 9 Little France Road, Edinburgh, UK, EH16 4UX

Cathie.Sudlow@ed.ac.uk

ORCID: 0000-0002-7725-7520

For peer review only

Abstract

Purpose: Generation Scotland (GS) is a large family-based cohort study established as a longitudinal resource for research into the genetic, lifestyle and environmental determinants of physical and mental health. It comprises extensive genetic, sociodemographic and clinical data from volunteers in Scotland.

Participants: A total of 24,084 adult participants, including 5,501 families, were recruited between 2006 and 2011. Within the cohort 59% are female, with an average age at recruitment of 49 years. Participants completed a health questionnaire and attended an in-person clinic visit where detailed baseline data was collected on lifestyle information, cognitive function, personality traits and mental and physical health. Genotype array data is available for 20,026 (83%) participants, and blood-based DNA methylation (DNAm) data for 18,869 (78%). Linkage to routine NHS datasets has been possible for 93% (N=22,402) of the cohort, creating a longitudinal resource that includes primary care, hospital attendance, prescription and mortality records. Multimodal brain imaging is available in 1,069 individuals.

Findings to date: GS has been widely used by researchers across the world to study the genetic and environmental basis of common complex diseases. Over 350 peer-reviewed papers have been published using GS data, contributing to research areas such as ageing, cancer, cardiovascular disease and mental health. Re-contact studies have built on the GS cohort to collect additional prospective data to study chronic pain, major depressive disorder and COVID-19.

Future plans: To create a larger, richer, longitudinal resource, "Next Generation Scotland" (NGS) launched in May 2022 to expand the existing cohort by a target of 20,000 additional volunteers, now including anyone aged 12+. New participants complete online consent and questionnaires and provide postal saliva samples, from which genotype and salivary DNA methylation array data will be generated. The latest cohort information and how to access data can be found on the GS website (www.generationscotland.org).

For peer review only

Strengths and Limitations

- The GS cohort has a wide range of demographic, lifestyle, health, and genetic data, and includes participants from a wide range of sociodemographic backgrounds.
- Linkage to a variety of longstanding, routine NHS datasets, which are particularly rich and diverse in Scotland, creates a breadth of longitudinal phenotype information.
- Planned linkages to medical images and radiology reports from across the NHS in Scotland will enrich the linked health data, while additional linkages to administrative data (e.g., education records) will broaden the information on participants and support research on the wider determinants of health and well-being.
- The cohort is relatively small, by contemporary standards for population-based cohorts. However, this issue can often be addressed through joint analyses with other population-based cohorts and participation in genetic data consortia.

For peer review only

Introduction

Generation Scotland (GS) is a longitudinal health study established as a family- and population-based resource for the study of the genetic, lifestyle and environmental determinants of common complex diseases. Non-communicable diseases, such as cancer, diabetes, stroke, heart, liver and lung disease are the leading cause of morbidity and mortality in Scotland [1,2]. The majority of common health disorders of public health concern are a result of a complex interaction between genes and environment. The GS cohort is rich in genetic and phenotypic information through data collection, sample assays and linkage to routine electronic health records. As a bio-resource for medical research, GS aims to support research to establish the determinants of physical and mental health and improve the prevention, diagnosis and treatment of common diseases.

GS was founded as a multi-institutional, cross-disciplinary collaboration between the Universities of Aberdeen, Dundee, Edinburgh and Glasgow, and the National Health Service (NHS) Scotland, with key resources, expertise and input from the Medical Research Council Human Genetics Unit, the National eScience Centre and the Scottish School of Primary Care. A list of current staff working on developing and maintaining the GS resource and members of the Scientific Steering Committee is provided in Appendix A.

Between 2006 and 2011, over 24,000 adult volunteer participants completed questionnaires, attended a clinic visit or participated by post, and consented to genetic studies, linkage to their medical records and to be re-contacted for future research [3].

In 2022 GS launched Next Generation Scotland (NGS), aiming to expand the existing cohort by recruiting 20,000 new participants, newly including 12-17 year olds, meeting an unmet need to study adolescent health. Data is collected via an online questionnaire and saliva sample collection by post.

An earlier cohort profile paper described the baseline recruitment [4]. Here we report data enrichment of the cohort, including new biological data, longitudinal data linkage and re-contact studies. We highlight the extent and nature of the data now available to researchers, summarise the use and impact of GS since commencement in 2006, and outline the current and future plans for NGS.

For peer review only

Cohort Description

Participant recruitment

The original GS: Scottish Family Health Study (SFHS) protocol and baseline data profile have been described previously [3,4]. Briefly, potential participants aged 35-65 years (study probands) were selected from lists of collaborating general medical practices in Scotland. They were invited to participate in the study and asked to identify at least one adult (18+ years old) first-degree relative to invite to the study. This included volunteers from the Glasgow and Tayside areas from 2006-2011 and was extended to include Ayrshire, Arran and Grampian in 2010. Participants completed a pre-clinic questionnaire (PCQ) before attending a research clinic in Glasgow, Dundee, Perth, Aberdeen or Kilmarnock. In total, 126,000 individuals were invited to participate, of whom 6,665 responded and met the study criteria (response rate of 5.3%). An additional 17,419 family members were recruited via these probands. The original GS:SFHS cohort therefore consists of 24,084 participants.

Baseline data collection

All 24,084 participants completed a PCQ collecting a range of demographic, social characteristics, personal behaviours, and self-reported health data. Information collected included smoking status, alcohol consumption, and personal and family disease history. Information was also collected on the birthplaces, by local council area, of participants and their parents and grandparents born in Scotland. In 2009, revisions were made to several questions within the PCQ for machine readability; the period prior to this is was termed phase 1 (N=9,967) and the period thereafter phase 2 (N=14,117).

Most participants (21,476) also attended a research clinic, where physical measurements included height, weight, heart rate, systolic and diastolic blood pressure, electrocardiogram (ECG), and body composition analysis. Standardised and well-validated assessments of cognitive function, personality, and mental health included the 28-item General Health Questionnaire, Eysenck personality questionnaire, Structured Clinical Interview for DSM Disorders, Mood Disorder Questionnaire, Schizotypal Personality Questionnaire, Mill Hill Vocabulary test and WAIS-III logical memory test. All baseline measures collected are reported in the previous cohort profile [4].

Cohort characteristics at recruitment

Of the 24,084 participants within the original GS:SFHS cohort, 59% are female, with an average age at recruitment of 49 years. In total, 87% of participants were born in Scotland and 97% born within the UK. The cohort includes a range of socio-demographic characteristics, although compared to the Scottish population participants have a higher education level and lower deprivation index (Table 1).

Family groups of at least two first-degree relatives were identified and assigned a shared family identity number. Pedigrees were constructed using relationship information provided by study participants and validated with genetic kinship information following genotyping. The cohort contained 1,361 singletons (with no relatives in the study) and 5,501 families of at least two people, with a mean size of 4.1 family members.

For peer review only

Table 1: Demographic and lifestyle characteristics of GS:SFHS cohort (N=24,084) and comparison to the Scottish population.
[a] Based on 2011 Scottish Census [5–7].
[b] Based on National Records of Scotland (NRS) Population Estimates by Scottish Index of Multiple Deprivation (SIMD) [8].
[c] Based on NRS Scottish Government Urban Rural Classification 2020 [9].
[d] Based on the ONS Annual Population Survey 2021 [10]. Individuals aged 18-64.
[e] Based on The Scottish Health Survey 2021 [1].

Characteristic	N	% (or median and IQR)	Scotland average
Sex			
Female	14,157	58.8%	51.5%[a]
Male	9,927	41.2%	48.5%[a]
Age			
Median Age (interquartile range, IQR)	24,084	49 (36 - 59)	41.3[a]
Ethnicity			
White	22,826	94.8%	96.0%[a]
Other	262	1.1%	4.0%[a]
Missing	996	4.1%	
SIMD quintiles			
5 (Least Deprived)	6,571	27.3%	20.6%[b]
4	5,419	22.5%	21.1%[b]
3	3,437	14.3%	19.8%[b]
2	2,987	12.4%	19.3%[b]
1 (Most Deprived)	2,724	11.3%	19.2%[b]
Missing	2,946	12.2%	
Rural Urban Classification codes			
1 - Large Urban Areas	7,369	30.6%	37.8%[c]
2	6,679	27.7%	33.9%[c]
3	2,032	8.4%	8.6%[c]
4	1,006	4.2%	2.6%[c]
5	2,662	11.1%	11.6%[c]
6 - Remote Rural Areas	1,390	5.8%	5.5%[c]
Missing	2,946	12.2%	
Employment Status (up to 75 years)			
Employed (full or part-time)	14,808	64.6%	73.2%[d]
Unemployed	970	4.2%	3.90%[d]
Retired	3,080	13.4%	
Other	1,808	7.9%	
Missing	2,255	9.8%	
Education - Highest qualification attained			
No qualification	3,145	13.3%	26.8%[a]
Lower secondary school	3,611	15.3%	23.1%[a]
Higher secondary school	2,452	10.4%	14.3%[a]
College level	6,450	27.3%	9.7%[a]
University level	7,330	31.0%	26.1%[a]
Other	624	2.6%	

Missing	1,738	7.2%	
Smoking Status			
Current Smoker	3,997	16.6%	11.3% [e]
Ex-Smoker	6,964	28.9%	23.2% [e]
Non-Smoker	12,227	50.8%	65.6% [e]
Missing	896	3.7%	
Alcohol consumption			
Median alcohol units per week (IQR)	21,737	8 (2 - 15)	6.1 [e]

Longitudinal Data linkage

Linkage to extensive and longstanding NHS Scotland records, both retrospective and prospective, creates a longitudinal cohort from baseline. The linkage data available within GS has not previously been described. At the time of writing, participants have up to 16-years follow-up data since recruitment in 2006. Routine NHS data is obtained through collaboration with the Health Informatics Centre (HIC) at the University of Dundee, with linkage performed using the community health index (CHI) number. CHI numbers are used across NHS Scotland services and are unique to each GP-registered individual living in Scotland. In total, 93% of GS participants consented to linkage and had a CHI number available. For individuals with CHI linkage, 89% also have genome-wide genotype data available (see Laboratory Samples and Molecular Assays below).

Table 2 and Figure 1 shows the range of datasets linked to the GS cohort, their periods of coverage and the numbers of participants with linked data available for each. Additional details are provided in Appendix B. Beyond linkages to hospital episodes, primary care, cancer and death registries and community electronic prescribing, GS has linkage to a range of other datasets via participants' CHI numbers, including routine laboratory tests, dental data (from the Management Information & Dental Accounting System, MIDAS) and the Scottish Drug Misuse Database (SDMD), offering unique phenotype information distinct from other population-based cohort research resources. COVID-19 testing, diagnoses and vaccination records are also available for the period of 2020 to 2022.

Regular data refreshes are received and new datasets are added to enhance and continue the follow-up of participants over time. Planned additional linkages include incorporating NHS Scotland routine NHS radiology images, including X-rays, computerised tomography (CT) and magnetic resonance imaging (MRI) scans (Scottish Medical Imaging, SMI), imaging reports, and retinal scans, which will provide new research opportunities not available in other population-based cohorts. Text-based radiology report linkage has already been applied to a study of stroke phenotyping in GS participants [11].

For peer review only

Table 2: Summary of linked data sources. Total number of participants and proportion of individuals with genome wide genotyping data available for all linked data.
[a] Scottish national telehealth and telecare organisation.
[b] Scottish Intensive Care Society Audit Group (SICSAG).
[c] National Records of Scotland (NRS).
[d] Scottish Care Information – Diabetes Collaboration (SCI-DC).
[e] Prescribing Information System (PIS). Shaded portion represents incomplete coverage for data up to 2009.
[f] Electronic Communication of Surveillance in Scotland (ECOSS).
[g] Management Information & Dental Accounting System (MIDAS).

	Total Participants	Genome-wide genotype data available	
		N	%
CHI Linkage	22,403	19,960	89.1%
**Scottish Morbidity Records**			
Outpatient Attendance (SMR00)	21,271	19,159	90.1%
General/Acute Inpatient and Day Case (SMR01)	18,249	16,467	90.2%
Maternity Inpatient and Day Case (SMR02)	8,239	7,537	91.5%
Mental Health Inpatient and Day Case (SMR04)	578	516	89.3%
Scottish Cancer Registry (SMR06)	3,606	3,207	88.9%
Scottish Birth Record (SMR11)	3,246	2,864	88.2%
**Primary Care**			
General Practice (GP)	19,676	17,823	90.6%
GP Out of Hours	8,533	7,700	90.2%
NHS24[a]	12,326	11,108	90.1%
Accident and Emergency (A&E)	15,249	13,778	90.4%
**Other Datasets**			
Routine Laboratory Test Results	19,090	17,521	91.8%
ICU Episode Data (SICSAG)[b]	361	324	89.8%
Deaths (NRS deaths data)[c]	1,659	1,376	82.9%
Diabetes Registry (SCI-DC)[d]	1423	1,241	87.2%
Prescription Dispensing (PIS)[e]	21,486	19,347	90.0%
COVID-19 Vaccinations	19,128	17,358	90.7%
COVID-19 Testing (ECOSS)[f]	16,537	14,995	90.7%
Dental (MIDAS)[g]	19,871	17,904	90.1%
Scottish Drug Misuse Database (SDMD)	76	63	82.9%

Cohort morbidities

Participant self-reported disease prevalence (at recruitment) is shown in Table 3 alongside longitudinal data on morbidities obtained through data linkage to primary care (GP) data, Scottish morbidity records (SMR) and National Records of Scotland (NRS) death records. ICD and Read Codes to define disease prevalence were derived from Gadd et al [12] using CALIBER code lists, detailed in full in Appendix Tables C-E. Data is available up to 2020 for GP records, cancer registries and 2022 for hospital admissions (SMR01) and mortality records. Diagnoses of 3,006 hypertension, 2,197 asthma, 2,371 depression, 2,558 osteoarthritis and 1,701 heart disease cases are reported across all primary and secondary care, and mortality linked data sources.

As an extended example, Figure 2 shows the proportion of diabetes cases captured in secondary care and deaths records as described above and enhanced with additional data sources available within GS (The Scottish Care Information – Diabetes Collaboration (SCI-DC), the Prescribing Information System (PIS) and routine laboratory testing data). A total of 1,861 diabetes (type 1 and 2) cases were recorded in at least one source, cohort prevalence of 7.7%. The SCI-DC captures 74% of all recorded cases. Prescriptions of Metformin Hydrochloride or Insulin within the PIS captured 73% of diabetes cases. Linked routine laboratory testing data contained results for any glycated haemoglobin (HbA1c) tests conducted as an indication of average blood sugar levels. Individuals with percentage glycated haemoglobin in blood (HbA1c levels) above 6.5 (48mmol/mol) was classified as diabetic (28% of cases captured) [13]. We note that lower proportions of cases within the self-reported source reflects that these were collected at baseline while other sources extend to 2020/2022. Use of a combination of data sources provides an opportunity to capture a range of cases and develop detailed phenotype definitions.

For peer review only

Table 3: Self-reported prevalence of common morbidities at baseline and morbidities in GS participants using linked data up to 2022. ICD and Read Code lists are detailed in full in Appendix Tables C-E.

	Baseline Self-Reported Morbidities 2006-2011		Morbidity Diagnoses from NHS Linked Data Sources 1980-2022[d]				
**Disease Outcome**	**N**	**% Participants**	**Primary Care (GP Data)**	**General/Acute Inpatient and Day Case (SMR01)**	**NRS Deaths Data**	**Scottish Cancer Registry (SMR06)**	**Total[e]**
Hypertension	3,257	13.8%	1,669	2,436	160		3,464
Asthma	2,652	11.2%	1,719	1,111	10		2,292
Depression[a]	2,196	9.3%	2,139	334	<10		2,346
Osteoarthritis	1,783	7.5%	1,861	1,639	<10		2,817
Heart Disease	935	3.9%	960	1,673	342		1,885
Diabetes	804	3.4%	536	968	149		1,200
Rheumatoid arthritis	431	1.8%	199	207	16		322
Stroke	352	1.5%	514	434	72		753
Breast Cancer	345	1.5%	355	596	82	541	650
COPD[b]	276	1.2%	554	693	150		952
Bowel Cancer	142	0.6%	155	245	39	213	298
Prostate Cancer	104	0.4%	164	271	53	252	323
Lung Cancer	63	0.3%	94	205	152	174	234
Dementia[c]	41	0.2%	198	245	175		356
COVID-19			<10	145	38		162

[a] Phase 1 participants (N=9,967) were asked if they have been diagnosed with depression and in phase 2 (N=14,117) if they had been diagnosed with severe depression

[b] Chronic obstructive pulmonary disease (COPD) data for phase 2 only (N=14,117).

[c] Participants were asked if they have been diagnosed with Alzheimer's disease.

[d] Dataset date coverage: GP data (1980-2020), SMR01 (1980-2022), National Records of Scotland (NRS) deaths data (2007-2022), SMR06 (1996-2020), SMR04 (1980-2020).

[e] Deduplicated across data sources.

Laboratory samples and molecular assays

Participants who attended a research clinic also provided biological samples (including blood and urine) for genotyping and other assays (N=23,979). Saliva was provided for DNA extraction by a subset of participants not attending a clinic (2,608 sent a saliva sample by post) and was used for DNA extraction for an additional 984 participants from whom blood could not be obtained (total 3,592). DNA was extracted from blood and saliva for 85% of participants (N= 20,471). Basic biochemistry assays were performed on the baseline serum samples measuring creatinine, glucose, potassium, sodium, urea, and cholesterol levels. Here we provide an update on the genotyping methods conducted since baseline collection.

Genomics

Genome-wide genotyping data is available for 20,026 (83%) of the original GS:SFHS participants [4]. Samples were genotyped using the Illumina HumanOmniExpressExome8v1-2_A and HumanOmniExpressExome-8v1_A and the Beadstudio-Gencall v3 genotype calling algorithm. Quality control measures were implemented, filtering out samples with a call rate of <98% and SNPs with a call rate of <98%, HWE of <1x10-06 and MAF of ≤1%, leaving 20,026 samples and 630,207 SNPs. Phasing of the genotyped SNPs was carried out using SHAPEIT2 [14].

Genetic profiles have been imputed using three different reference panels: 1000 Genomes (1000G) [15], Haplotype Reference Consortium (HRC) [16] and Trans-Omics for Precision Medicine (TOPMed) (Table 4) [17]. After imputation, further quality control procedures have removed duplicate and monomorphic SNPs and those with an imputation quality score of <0.4.

Table 4: GS genotyping and imputation summary for three imputation panels.

	1000G	HRC	TOPMed
Reference panel version	1000G Phase 1 v3	HRC.r1-1	TOPMed Freeze5
Imputation software	IMPUTE2	PBWT	Minimac4
Build	GRCh37	GRCh37	GRCh38
Number of SNPs (post QC)	9,438,897	24,161,581	64,616,987
Indels	✗	✗	✓

Methylomics

DNA methylation (DNAm) data has been generated using the Illumina HumanMethylationEPIC BeadChip array for 18,869 GS samples at > 850,000 CpG sites, from blood collected at the baseline appointment (2006-2011). At the time of writing, this is the largest DNA methylation dataset from a single population-based cohort. These samples were processed in four batches between 2017 and 2021, and are referred to as set 1 (N=5,087), set 2 (N=459), set 3 (N=4,450) and set 4 (N=8,873). A subsequent genome-wide DNA methylation measurement is also available for 880 individuals across set 2 (N=508) and set 3 (N=372), from additional blood collected between 2015 and 2019. The DNA methylation resource will be described in detail in a separate report. Briefly, quality control was carried out in R using the packages ShinyMethyl and WateRmelon. Probes with a bead count of less than three or a high detection P value (>0.05) in more than 5% of samples were removed. Outlier probes were also removed based on visual inspection of the log median intensity of the methylated versus unmethylated signal per array. Samples were removed where there were sex mismatches or

where 1% or more of cytosine-guanine dinucleotides had a high detection P value (>0.05). A superset of 18,869 baseline samples has also been generated from the four individual sets, comprising 831,733 CpGs that passed quality control in all sets.

Proteomics and metabolomics

Protein levels have been quantified in plasma samples from 1,065 participants using the 5k SOMAscan v4 array from SomaLogic Inc. Tandem mass spectrometry (MS/MS) has been performed on a subset of 860 participants' blood samples for which peripheral blood mononuclear cells (PBMCs) were available. Quantification of 54 urinary metabolite biomarkers in 2,743 GS participants' samples has been conducted by Nightingale Health using nuclear magnetic resonance (NMR).

Re-contact studies

Participants provided broad consent permitting use of data and samples for 'future medical research into health, illness and medical treatment'. This included consent to be re-contacted for new studies, which has led to additional data collections since recruitment, summarised in Table 5. Data from re-contact studies can be linked to GS data and are retained by GS to be made available for other researchers through the GS access process.

The Stratifying Resilience and Depression Longitudinally (STRADL) sub-study recruited from the existing GS cohort to subtype major depressive disorder (MDD), using detailed clinical, cognitive, and brain imaging assessments. From 2015-2017, 9,905 GS participants completed a remote depression-focused questionnaire (including psychological resilience, coping style, and response to psychological distress) and a subset (N=1,189) attended a face-to-face assessment to conduct cognitive testing, multimodal MRI brain scans (N=1,085) and further bio-sample collection [18].

In 2016, the DOLORisk study enhanced GS to study neuropathic pain (NP). The study received responses to a survey regarding presence or absence of chronic pain and NP from 7,238 of 20,221 members of the GS cohort invited to participate (35.8% response rate), with a follow-up repeat survey (N= 5,292 responses) after 18 months (Table 5) [19].

GS is a member of the European Prevention of Alzheimer's Dementia Consortium (EPAD), an interdisciplinary research initiative with partners across European organisations aiming to improve the understanding of the early stages of Alzheimer's disease [20]. In 2016, 53 GS participants attended a 'screening visit' for the collection of fasting blood samples and a brain scan (MRI) with follow up visits after 6 months, 1 year, 2 years, 3 years, and 4 years.

GS is partnering with Healthy AGeing in Scotland (HAGIS), a study of the health, economic and social circumstances of people over 50 years old in Scotland [21]. HAGIS is part of the Health & Retirement Study family of longitudinal ageing studies which currently consists of longitudinal ageing studies in 16 countries around the world. GS re-contacted 14,891 individuals in 2021-22, with 2,826 (19.0%) taking part in the HAGIS: COVID-19 Impact & Recovery Study.

Additional data collections conducted by the GS team include the CovidLife surveys launched in April 2020 in response to the COVID-19 pandemic. The aim was to determine the impact of the pandemic on health and well-being. In total, 18,518 adult members of the UK public, including 4,968 GS participants, participated in the surveys. Three CovidLife surveys [22] and a Rural CovidLife survey [23] specific to rural Scottish volunteers (Total N=3,365, GS participants N=712). In addition, three TeenCovidLife surveys [24], for young people aged 12-18 (N = 7,058), were run between April 2020 and June 2021. GS was part of the National Core Studies Longitudinal Health and Wellbeing programme established as part of the UK's pandemic response, including the coronavirus post-acute

For peer review only

long-term effects: constructing an evidence base (CONVALESCENCE) long COVID study [25]. GS is also a participating cohort in COVIDMENT, a large-scale collaborative project between Northern European countries using data-rich population-based registry resources, biobanks, and ongoing questionnaire data to further understanding of the mental health impact of the COVID pandemic [26].

Table 5: Summary of re-contact studies and the number of participating GS volunteers. The number of eligible GS participants invited to each study varies due to study criteria. All eligible GS participants consented to re-contact.

Study Eligibility Criteria:

[a] Known email or postal address.

[b] Participated in the baseline DOLORisk survey and consented to re-contact for the follow-up survey.

[c] Living in Scotland and had a valid CHI number.

[d] Indicated willingness to undergo face-to-face assessment in the remote STRADL questionnaire.

[e] Resident in Scotland, known email address or postal address.

[f] Living in rural Scotland.

[g] Aged 50+, no diagnosis of dementia or medical/psychiatric disorders.

[h] Aged 50+, living in Scotland.

Re-contact study		Study Dates	GS participants eligible for re-contact	GS Participants (% Responded)
DOLORisk	Baseline questionnaire	May 16-Dec 16	20,221[a]	7,238 (35.8%)
	Follow up survey	Jun 18-Jun 19	6,657[b]	5,292 (79.5%)
STRADL	Remote questionnaire	2015-2017	21,525[c]	9,905 (46.0%)
	Face-to-face clinic visit		9,618[d]	1,189 (12.4%)
COVIDLife	COVIDLife1 survey	Apr 20-Jun 21	22,796[e]	4,968 (21.8%)
	Rural COVIDLife survey	Oct 20-Nov 20	1,559[f]	712 (45.7%)
EPAD	Alzheimer's dementia	2016-2018	3,779[g]	53 (1.4%)
HAGIS	COVID impact survey	2021-2022	14,891[h]	2,826 (19.0%)

For peer review only

New Recruitment and Data Collection Plans

In 2019, funding was obtained from the Wellcome Trust to expand the GS cohort using remote data collection and extended eligibility to younger individuals (12+ years). Because of the COVID-19 outbreak in 2020, field studies other than those directly relating to the pandemic were paused. Active recruitment of new volunteers to join GS started in May 2022. Original GS cohort members have been contacted with the option to move online to complete new questionnaires and invite friends and family members to join the next phase of the study (snowball recruitment). Other recruitment methods to date have included: email invitations to Scotland-based participants of the COVIDLife study, news coverage (TV segments, radio, newspaper and online news articles), a paid TV advertisement and social media advertising.

NGS aims to recruit 20,000 new participants and will use established methods for linkage to routine NHS data to create a larger, richer, longitudinal resource. Anyone living in Scotland aged 12 years and over is eligible to join; those aged 12-15 require parental confirmation of their capacity to consent. Participants sign-up on our online portal, complete study consents and a baseline questionnaire to collect lifestyle measures and medical history.

Saliva samples are being collected by post for genotyping of new participants. At the time of writing over 10,000 new participants have been recruited, adding to the 2006-2011 cohort recruits.

Adolescence and early adulthood are critical periods in the development of mental and physical health [27]. The extension to younger individuals, along with potentially other family members, will make the cohort a valuable resource for research into genetic and environmental determinants of health among adolescents and young adults. There are few comparable genetic cohorts using routine data linkage in young people. Early approved studies are planned to focus on mental health, sleep and loneliness in this age group.

New questionnaires will be regularly added to the online portal to enable ongoing engagement with participants and collect enhanced data such as cognitive testing. Researchers will be able to submit approved research questions for prospective data collections. Through a broad range of recruitment strategies, participant involvement and engagement and the use of remote data collection, we hope to improve geographic coverage and sociodemographic diversity across Scotland, aiming to engage groups typically under-represented in large-scale studies. Completion of the expansion phase, combined with the original GS participants, should create an overall cohort of over 40,000 individuals across Scotland with rich genetic and phenotypic data.

Participant and Patient Involvement

A key component of the GS:SFHS was to conduct a public consultation programme which was used to ask the public their thoughts on genetics in healthcare and research and use this to develop principles of participation and data access [28,29]. Regular newsletters are distributed to participants to provide updates on the latest cohort information and recent findings. Patient and public involvement and engagement (PPIE) is being developed within the new NGS cohort recruitment. A survey receiving 1,000 responses invited participants to become GS ambassadors in their local area, take part in focus groups and test new questionnaires. These volunteers have already helped with survey testing and provided feedback on recruitment materials. Development of a Young Persons Advisory Group (YPAG) has helped direct teen recruitment activities and will shape future GS health research with teenagers themselves.

For peer review only

Findings to Date

The GS cohort has facilitated research contributions to a wide range of health conditions and scientific areas including aging, cancer, cardiovascular disease, mental health and the role of DNA methylation in understanding and predicting disease. Over 350 papers have been published using GS data (Figure 3). Appendix F lists the 50 most cited papers using GS data and a full and growing publication list can be found on the GS website (https://www.ed.ac.uk/generation-scotland/what-found/publications). Examples of some key contributions are summarised below.

Welsh et al used residual blood samples from GS participants (N=19,501) to assay cardiac troponin T (cTnT) and troponin I (cTnI), proteins essential for heart contraction, and to investigate their association with cardiovascular outcomes [30]. The research team identified deaths or hospitalisations of interest using Scotland's morbidity records and deaths data from GS recruitment to September 2017. They found that cTnT and cTnI were both associated with heart failure and cardiovascular disease death. Individuals with high levels of cTnT were more likely to suffer from heart disease, stroke or other heart conditions. Troponin level testing is inexpensive, and this study demonstrated the potential benefit of testing for future health screening.

Given its size, the GS DNA methylation resource is well-placed to serve as a training dataset for the development of risk predictors. Cheng et al used GS methylation data to develop and validate a model for 10-year risk prediction of type 2 diabetes [31]. They combined standard risk prediction information such as age, sex, body mass index (BMI) and family history of the disease with DNA methylation data, which improved prediction for likelihood of developing diabetes. The results were tested using a hypothetical screening scenario of 10,000 people, which correctly classified an additional 449 individuals using methylation data compared with traditional risk factors alone.

Green et al investigated the aetiology of major depressive disorder among individuals from the STRADL re-contact study [32]. They reported the associations of serological and methylomic signatures of C-Reactive Protein (considered to represent acute and chronic measures of inflammation respectively) with depression status/symptoms, and structural neuroimaging phenotypes. The study provided evidence for the involvement of peripheral inflammation in brain morphology and depression symptoms, and demonstrated the combined use of survey, neuroimaging, serological and methylation data from the GS cohort.

GS facilitated a pilot study to investigate the use of newborn blood spots in longitudinal research. Heel prick blood spots are used routinely to test for treatable neo-natal metabolic conditions and have been retained in Scotland for all children born since 1965. Researchers showed that archival blood spots contain enough information to link to the volunteer health records and samples were of sufficient quality to generate biologically meaningful results [33]. For example, epigenetic signatures of perinatal maternal smoking status could be identified. This pilot study confirmed the feasibility of the use of these archived newborn blood spots in a population-level retrospective birth cohort study. It has the potential to scale to a linked collection of 3 million archived blood spots across Scotland, making it one of only two such resources available worldwide [34]. Future work is dependent on a Scottish government led public consultation to review the current pause on research access [35].

For peer review only - http://bmjopen.bmj.com/site/about/guidelines.xhtml

Strengths and Limitations

Important strengths of GS are the breadth of demographic, lifestyle and health factors, and inclusion of participants from a wide range of sociodemographic backgrounds. The cohort is rich in genetic and linkage data. Scotland is ideally suited to a longitudinal cohort study given its comparatively static and stable population and relatively high prevalence of common conditions and adverse lifestyle risk factors [1,2]. The family-based recruitment approach delivers increased kinship among participants and pedigree mapping enables measurement of heritability and familial aggregation of traits.

Linkage to a variety of routine NHS datasets creates a wealth of research opportunities, while participants' consent for future re-contact studies provides potential for additional data collections. Using linkage to gather longitudinal data makes the cohort more robust to attrition as passive linkage allows us to link to new data even if a participant does not take part in future data collections such as re-contact studies. Planned linkages to routine NHS medical images, radiology reports and administrative data, such as education, income and benefits, will provide uniquely rich information about the participants and its relationship with future health and wellbeing, further enhancing the research potential of the cohort.

There are some limitations of the GS cohort. The cohort is relatively small, by contemporary standards for population-based cohorts, which can limit the statistical power to address some research questions definitively (e.g., to study rare diseases or small effect sizes). However, this issue can often be addressed through joint analyses with other population-based cohorts and participation in genetic data consortia, which GS actively contributes to. The current expansion of the cohort will also help to address this limitation. Many phenotypes are assessed using self-reported measures which may be subject to recall or response bias. These potential biases are minimised in GS by using validated questionnaires applied widely in research and confirmation of outcomes through linkage to medical records. Compared to the Scottish population, individuals in the cohort are generally older, more likely to be female and less socially deprived. This may limit the power of research studies to pick up relationships with health outcomes and factors such as education/deprivation at the lowest ends of the scale. However, it is hoped that increased diversity of the cohort will be achieved with the current expansion to reach a total cohort size of over 40,000 individuals in Scotland. GS aims to be the UK's largest multi-generational longitudinal life-course study of genetic, epigenetic, clinical, lifestyle and environmental health determinants.

Data Access and Collaborations

Researchers can submit proposals to access GS data and samples through our website (https://www.ed.ac.uk/generation-scotland/for-researchers). This also includes data from re-contact studies which can be accessed through a single application to GS. Research proposals are subject to review by the GS access process, under the guidance of the Scientific Steering Committee, based on criteria set out in the management, access and publications policy. We welcome proposals for data and sample access, and for prospective data collections using the NGS online portal. Further information about the cohort, details of the application process, and conditions for access are available at the study website.

Generation Scotland also collaborates with – and makes its data and/or metadata available via – the Dementias Platform UK (DPUK), UK Longitudinal Linkage Collaboration (UK LLC), CLOSER, BC Platforms and Health Data Research UK (HDR UK) Innovation Gateway. Access requests can be made through DPUK and UK LLC using the standard GS access process as well as directly to GS. All

applications via these platforms are reviewed by the GS access process. Study metadata is available through CLOSER Discovery, BC Platforms and HDR UK.

GS genetic data has contributed to large-scale consortia including Cohorts for Heart and Aging Research in Genomic Epidemiology (CHARGE)[36], Chronic Kidney Disease Genetics (CKDGen)[37], Genetic Investigation of ANthropometric Traits (GIANT)[38], SpiroMeta[39], Global Biobank Meta-analysis Initiative (GBMI)[40], COVID-19 host genetics initiative (HGI)[41], Global Lipids Genetics Consortium (GLGC)[42] and The Psychiatric Genomics Consortium (PGC) [43].

Funding

GS:SFHS was funded by a grant from the Chief Scientist Office of the Scottish Government Health Directorates [CZD/16/6] and the Scottish Funding Council [HR03006]. Genotyping of the GS:SFHS samples was carried out by the Genetics Core Laboratory at the Edinburgh Clinical Research Facility, University of Edinburgh, Scotland and was funded by the Medical Research Council UK and the Wellcome Trust (Wellcome Trust Strategic Award "STratifying Resilience and Depression Longitudinally" (STRADL) Reference 104036/Z/14/Z). NGS is funded by the Wellcome Trust (216767/Z/19/Z). DNAm analysis was funded by the Wellcome Trust (Reference 220857/Z/20/Z).

Ethics Statement

All components of Generation Scotland received ethical approval from the NHS Tayside Committee on Medical Research Ethics (REC Reference Number: 05/S1401/89). Generation Scotland has also been granted Research Tissue Bank status by the East of Scotland Research Ethics Service (REC Reference Number: 20-ES-0021), providing ethical approval for a wide range of uses within medical research. Written informed consent was obtained from all participants in GS:SFHS. NGS participants gave consent online. All participants aged 16 or over provide their own informed consent whilst those aged 12-15 require parental confirmation of their capacity to consent.

Contributorship Statement

The concept and design of Generation Scotland was developed by CH, REM, AMM, DJP, CS and HW ☐who obtained all funding, necessary ethical approvals and acted as principal investigators and chief scientist (HW). Acquisition and curation of data relating to Generation Scotland were conducted by AC, CFR, RF, DLM, HM and AR. SR led patient and public involvement and engagement activities including development of a Young Persons Advisory Group. HM drafted the manuscript. All authors critically revised the manuscript for important intellectual content and approved the final version for publication.

Data Sharing Statement

Data are available on reasonable request. Researchers may request access to Generation Scotland data through our website (https://www.ed.ac.uk/generation-scotland/for-researchers).

Competing Interests

Riccardo E Marioni is a scientific advisor to Optima Partners and the Epigenetic Clock Development Foundation. Daniel McCartney is a part-time employee of Optima Partners Ltd.

Acknowledgements

We are grateful to all the people and their families who have taken part in GS to date, the general practitioners and the Scottish School of Primary Care for their help in recruiting them, the GS Scientific Steering Committee, and the whole GS team, which includes interviewers, computer and

For peer review only

laboratory technicians, clerical workers, research scientists, volunteers, managers, receptionists, healthcare assistants and nurses.

For peer review only

Figure 1. Summary of linked data sources periods of coverage. Linkage dataset coverage ranges from 1975 to 2022.

[a] Scottish national telehealth and telecare organisation.
[b] Scottish Intensive Care Society Audit Group (SICSAG).
[c] National Records of Scotland (*NRS*).
[d] Scottish Care Information – *Diabetes* Collaboration (*SCI-DC*).
[e] Prescribing Information System (PIS). Shaded portion represents incomplete coverage for data up to 2009.
[f] Electronic Communication of Surveillance in Scotland (ECOSS).
[g] Management Information & Dental Accounting System (MIDAS).

Figure 2: 2A. Proportion of diabetes cases captured in each data source (total cases N=1,861). 2B. Venn diagram of the concordance of cases among data sources (N=1,852, excluding routine laboratory testing data for glycated haemoglobin (HbA1c) and deaths data).

SCI-DC - Scottish Care Information - Diabetes Collaboration.
PIS - Prescribing Information System.
SMR01 - Scottish Morbidity Records: General/Acute Inpatient and Day Case.
HbA1c - Routine laboratory testing data for haemoglobin A1c (level above 6.5%).
Self-report- prevalence reported by participant at baseline.
Deaths data – National records of Scotland deaths data.

Figure 3: Yearly number of access requests and publications using GS data over time.

For peer review only

References

1 The Scottish Government. The Scottish Health Survey 2021 Edition. 2021. http://www.gov.scot/publications/scottish-health-survey-2018-volume-1-main-report/ (accessed 29 March 2023)

2 The Scottish Government. Public Health Priorities for Scotland. 2018. https://www.gov.scot/binaries/content/documents/govscot/publications/corporate-report/2018/06/scotlands-public-health-priorities/documents/00536757-pdf/00536757-pdf/govscot%3Adocument/00536757.pdf (accessed 29 March 2023)

3 Smith BH, Campbell H, Blackwood D, *et al.* Generation Scotland: The Scottish Family Health Study; a new resource for researching genes and heritability. *BMC Med Genet*. 2006;7:1–9.

4 Smith BH, Campbell A, Linksted P, *et al.* Cohort Profile: Generation Scotland: Scottish Family Health Study (GS:SFHS). The study, its participants and their potential for genetic research on health and illness. *Int J Epidemiol*. 2013;42:689–700.

5 National Records of Scotland. Scotland 2011 census table: DC1117SC - Age by sex. 2011. https://www.scotlandscensus.gov.uk/documents/2011-census-table-data-scotland/

6 National Records of Scotland. Scotland 2011 census table: LC2101SC - Ethnic group by age. 2011. https://www.scotlandscensus.gov.uk/documents/2011-census-table-data-scotland/

7 National Records of Scotland. Scotland 2011 census table: LC5102SC - Highest level of qualification by sex by age. 2011. https://www.scotlandscensus.gov.uk/documents/2011-census-table-data-scotland/

8 National Records of Scotland. Population Estimates by Scottish Index of Multiple Deprivation (SIMD). 2022. https://www.nrscotland.gov.uk/statistics-and-data/statistics/statistics-by-theme/population/population-estimates/2011-based-special-area-population-estimates/population-estimates-by-simd-2016

9 National Records of Scotland. Population Estimates by Urban Rural Classification (2011 Data Zone based). 2022. https://www.nrscotland.gov.uk/statistics-and-data/statistics/statistics-by-theme/population/population-estimates/2011-based-special-area-population-estimates/population-estimates-by-urban-rural-classification

10 The Scottish Government. Scotland's Labour Market: People, Places and Regions – Protected Characteristics. Statistics from the Annual Population Survey 2021. 2022. https://www.gov.scot/publications/scotlands-labour-market-people-places-regions-protected-characteristics-statistics-annual-population-survey-2021/pages/1/

11 Davidson EM, Casey A, Grover C, *et al.* The epidemiological characteristics of stroke phenotypes defined with ICD-10 and free-text: a cohort study linked to electronic health records. *medRxiv*. 2023;2023.04.03.23288096.

12 Gadd DA, Hillary RF, McCartney DL, *et al.* Epigenetic scores for the circulating proteome as tools for disease prediction. *bioRxiv*. 2021;8:2020.12.01.404681.

13 Bennett CM, Guo M, Dharmage SC. HbA(1c) as a screening tool for detection of Type 2 diabetes: a systematic review. *Diabet Med*. 2007;24:333–43.

For peer review only

14 Delaneau O, Howie B, Cox AJ, *et al.* Haplotype estimation using sequencing reads. *Am J Hum Genet*. 2013;93:687–96.

15 Altshuler DM, Durbin RM, Abecasis GR, *et al.* An integrated map of genetic variation from 1,092 human genomes. *Nature 2012 491:7422*. 2012;491:56–65.

16 McCarthy S, Das S, Kretzschmar W, *et al.* A reference panel of 64,976 haplotypes for genotype imputation. *Nat Genet*. 2016;48:1279–83.

17 Taliun D, Harris DN, Kessler MD, *et al.* Sequencing of 53,831 diverse genomes from the NHLBI TOPMed Program. *Nature 2021 590:7845*. 2021;590:290–9.

18 Habota T, Sandu AL, Waiter GD, *et al.* Cohort profile for the STratifying Resilience and Depression Longitudinally (STRADL) study: A depression-focused investigation of Generation Scotland, using detailed clinical, cognitive, and neuroimaging assessments. *Wellcome Open Res*. 2021;4. doi: 10.12688/WELLCOMEOPENRES.15538.2

19 Hébert HL, Veluchamy A, Baskozos G, *et al.* Cohort profile: DOLORisk Dundee: a longitudinal study of chronic neuropathic pain. *BMJ Open*. 2021;11:e042887.

20 Solomon A, Kivipelto M, Molinuevo JL, *et al.* European Prevention of Alzheimer's Dementia Longitudinal Cohort Study (EPAD LCS): study protocol. *BMJ Open*. 2018;8:e021017.

21 Douglas E, Rutherford A, Bell D. Pilot study protocol to inform a future longitudinal study of ageing using linked administrative data: Healthy AGeing in Scotland (HAGIS). *BMJ Open*. 2018;8:e018802.

22 Fawns-Ritchie C, Altschul DM, Campbell A, *et al.* CovidLife: a resource to understand mental health, well-being and behaviour during the COVID-19 pandemic in the UK. *Wellcome Open Res*. 2021;6:176.

23 Stevenson AJ, Huggins CF, Forbes A, *et al.* RuralCovidLife: A new resource for the impact of the pandemic on rural Scotland. *Wellcome Open Res*. 2022;6:317.

24 Huggins CF, Fawns-Ritchie C, Altschul DM, *et al.* TeenCovidLife: a resource to understand the impact of the COVID-19 pandemic on adolescents in Scotland. *Wellcome Open Res*. 2021;6. doi: 10.12688/WELLCOMEOPENRES.17252.2

25 Bowyer RCE, Huggins C, Toms R, *et al.* Characterising patterns of COVID-19 and long COVID symptoms: Evidence from nine UK longitudinal studies. *Eur J Epidemiol*. 2023;38:199–210.

26 Unnarsdóttir AB, Lovik A, Fawns-Ritchie C, *et al.* Cohort Profile: COVIDMENT: COVID-19 cohorts on mental health across six nations. *Int J Epidemiol*. 2022;51:e108–22.

27 Solmi M, Radua J, Olivola M, *et al.* Age at onset of mental disorders worldwide: large-scale meta-analysis of 192 epidemiological studies. *Mol Psychiatry*. 2022;27:281–95.

28 Haddow G, Cunningham-Burley S, Bruce A, *et al.* Generation Scotland: consulting publics and specialists at an early stage in a genetic database's development. *Crit Public Health*. 2008;18:139–49.

29 Haddow G, Cunningham-Burley S, Murray L. Can the governance of a population genetic data bank effect recruitment? Evidence from the public consultation of Generation Scotland. *Public Understanding of Science*. 2011;20:117–29.

For peer review only

30 Welsh P, Preiss D, Hayward C, *et al.* Cardiac Troponin T and Troponin I in the General Population. *Circulation*. 2019;139:2754–64.

31 Cheng Y, Gadd DA, Gieger C, *et al.* Development and validation of DNA methylation scores in two European cohorts augment 10-year risk prediction of type 2 diabetes Check for updates. *Nat Aging*. 2023;3:450–8.

32 Green C, Shen X, Stevenson AJ, *et al.* Structural brain correlates of serum and epigenetic markers of inflammation in major depressive disorder. *Brain Behav Immun*. 2021;92:39–48.

33 Cunningham-Burley S, McCartney DL, Campbell A, *et al.* Feasibility and ethics of using data from the Scottish newborn blood spot archive for research. *Communications Medicine 2022 2:1*. 2022;2:1–8.

34 Nørgaard-Pedersen B, Hougaard DM. Storage policies and use of the Danish Newborn Screening Biobank. *J Inherit Metab Dis*. 2007;30:530–6.

35 The Scottish Government. Guthrie Cards in Scotland: Ethical, Legal and Social Issues. 2013. https://www.gov.scot/publications/guthrie-cards-scotland-ethical-legal-social-issues/documents/ (accessed 12 October 2023)

36 Psaty BM, O'Donnell CJ, Gudnason V, *et al.* Cohorts for Heart and Aging Research in Genomic Epidemiology (CHARGE) Consortium. *Circ Cardiovasc Genet*. 2009;2:73–80.

37 Köttgen A, Pattaro C. The CKDGen Consortium: ten years of insights into the genetic basis of kidney function. *Kidney Int*. 2020;97:236–42.

38 Yengo L, Vedantam S, Marouli E, *et al.* A saturated map of common genetic variants associated with human height. *Nature*. 2022;610:704–12.

39 Repapi E, Sayers I, Wain L V, *et al.* Genome-wide association study identifies five loci associated with lung function. *Nat Genet*. Published Online First: 2009. doi: 10.1038/ng.501

40 Zhou W, Kanai M, Wu K-HH, *et al.* Global Biobank Meta-analysis Initiative: Powering genetic discovery across human disease. *Cell Genomics*. 2022;2. doi: 10.1016/j.xgen.2022.100192

41 The COVID-19 Host Genetics Initiative. The COVID-19 Host Genetics Initiative, a global initiative to elucidate the role of host genetic factors in susceptibility and severity of the SARS-CoV-2 virus pandemic. *European Journal of Human Genetics 2020 28:6*. 2020;28:715–8.

42 Graham SE, Clarke SL, Wu K-HH, *et al.* The power of genetic diversity in genome-wide association studies of lipids. *Nature*. 2021;600:675–9.

43 Sullivan PF, Agrawal A, Bulik CM, *et al.* Psychiatric Genomics: An Update and an Agenda. *Am J Psychiatry*. 2018;175:15.

For peer review only

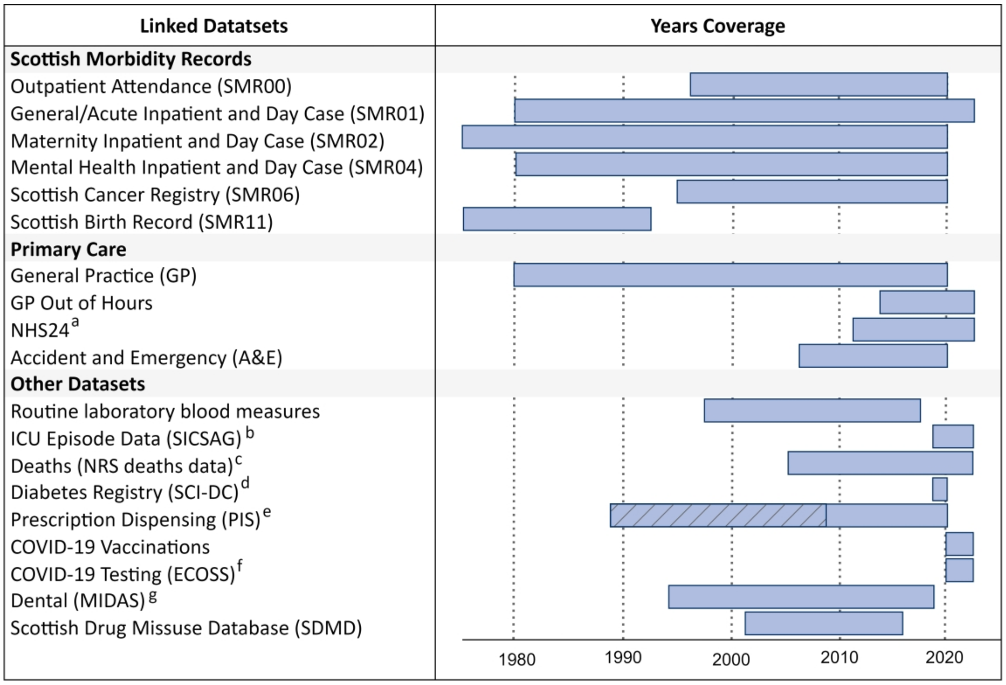

Figure 1. Summary of linked data sources years of coverage. Linkage dataset coverage ranges from 1975 to 2022.[a]Scottish national telehealth and telecare organisation.[b]Scottish Intensive Care Society Audit Group (SICSAG).[c]National Records of Scotland (NRS).[d]Scottish Care Information – Diabetes Collaboration (SCI-DC).[e]Prescribing Information System (PIS). Shaded portion represents incomplete coverage for data up to 2009.[f]Electronic Communication of Surveillance in Scotland (ECOSS).[g]Management Information & Dental Accounting System (MIDAS).

1546x1049mm (72 x 72 DPI)

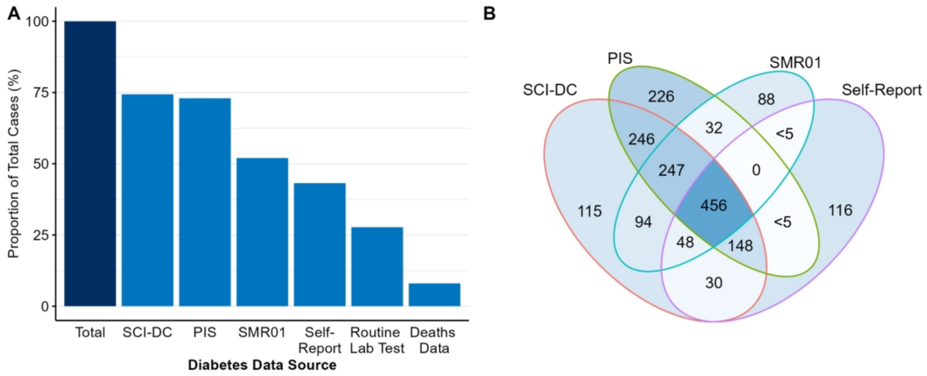

Figure 1: 1A. Proportion of diabetes cases captured in each data source (total cases N=1,861). 1B. Venn diagram of the concordance of cases among data sources (N=1,852, excluding routine laboratory testing data for glycated haemoglobin (HbA1c) and deaths data).
SCI-DC - Scottish Care Information - Diabetes Collaboration.
PIS - Prescribing Information System.
SMR01 - Scottish Morbidity Records: General/Acute Inpatient and Day Case.
HbA1c - Routine laboratory testing data for haemoglobin A1c (level above 6.5%).
Self-report- prevalence reported by participant at baseline.
Deaths data – National records of Scotland deaths data.

678x284mm (130 x 130 DPI)

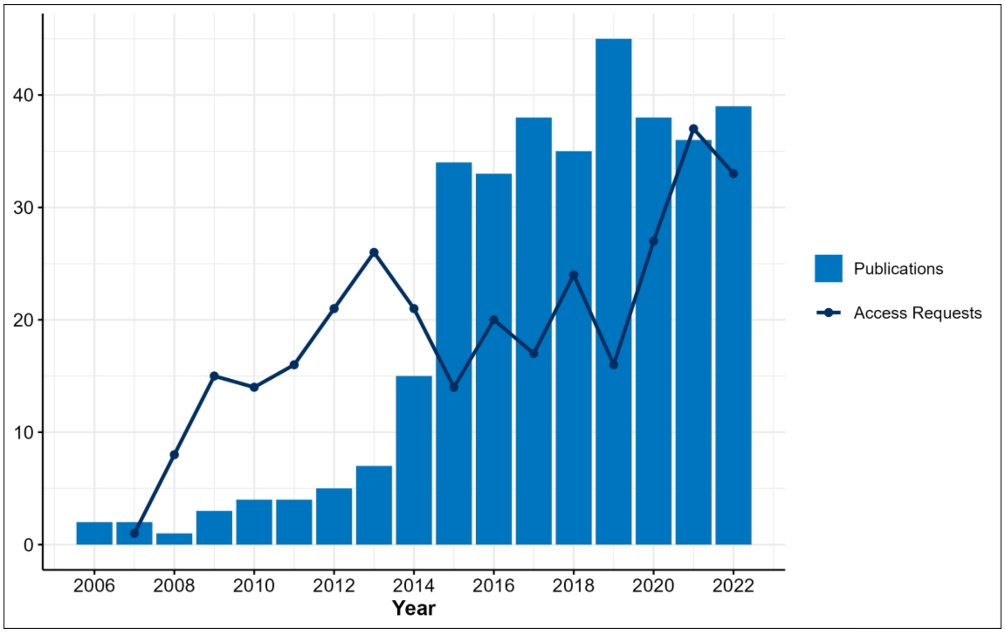

Figure 2: Yearly number of access requests and publications using GS data over time.

516x322mm (118 x 118 DPI)

Supplementary Material

Appendix A. List of current GS management and delivery team, investigators and members of the Scientific Steering Committee.

**GS Scientific Steering Committee**
Professor Dame Anna F Dominiczak (University of Glasgow & Chief Scientist Office, Scotland) – Chair<br>Professor Cathie Sudlow (University of Edinburgh) – GS Director<br>Professor Heather Whalley (University of Edinburgh) – GS Chief Scientist<br>Professor Julie Brittenden (University of Glasgow)<br>Dr Christian Cole (University of Dundee)<br>Professor Riccardo Marioni (University of Edinburgh)<br>Professor Zosia Miedzybrodzka (University of Aberdeen)<br>Professor Sandosh Padmanabhan (University of Glasgow)<br>Professor Blair Smith (University of Dundee)<br>Katie Wilde (University of Aberdeen)
**GS Scientific Leadership Team**
Professor Cathie Sudlow – GS Director and Principal Investigator (PI)<br>Professor David Porteous - Co-Investigator (Former Director and PI)<br>Professor Andrew McIntosh- Co-Investigator<br>Professor Riccardo Marioni- Co-Investigator<br>Professor Caroline Hayward- Co-Investigator<br>Professor Heather Whalley – GS Chief Scientist and Co-Investigator
**GS Management and Delivery Team**
Professor Cathie Sudlow (University of Edinburgh) – Director<br>Archie Campbell (University of Edinburgh) - Chief Technology Officer<br>Robin Flaig (University of Edinburgh) - Chief Operations Officer<br>Dr Daniel McCartney (University of Edinburgh) - Senior Bioinformatics Analyst<br>Professor Heather Whalley (University of Edinburgh) - Chief Scientist
Fiona Clark (University of Edinburgh) – Partnership Coordinator<br>Elly Darrah (University of Edinburgh) – Administrator<br>Liz Kirby (University of Edinburgh) - Research Support Officer<br>Hannah Milbourn (University of Edinburgh) - Health Data Scientist<br>Anne Richmond (University of Edinburgh) - Bioinformatics Analyst<br>Sarah Robertson (University of Edinburgh) - Young Person Engagement & Recruitment Co-ordinator<br>Rosie Tatham (University of Edinburgh) – Administrator<br>Alex Wood (University of Edinburgh) - Data Analyst / Developer<br>Dr Zhuoni Xiao (University of Edinburgh) – Research Fellow<br>Leah Young (University of Edinburgh) - Research Support Officer

For peer review only

Appendix B. Full list of available linked NHS datasets and descriptions.

[a] Scottish National healthcare data is collected as a series of Scottish Morbidity Records (SMR).

Linked Dataset	Coding	Date Range	N	Description
**Scottish Morbidity Records (SMR)**				
Outpatient Attendance (SMR00)[a]	None	1996-2020	21,270	Outpatient attendance information excluding A&E and genitourinary medicine.
General/Acute Inpatient and Day Case (SMR01)[a]	ICD9/ICD10	1980-2022	19,611	Acute inpatient and daycase hospital admissions: disease and procedure codes. Scotland-wide, around 750 000 admissions per annum since 1981.
Maternity Inpatient and Day Case (SMR02)[a]	ICD9/ICD10	1975-2020	8,238	Pregnancies and births: disease and procedure codes. Scotland-wide, around 58,000 deliveries per annum since 1975.
Mental Health Inpatient and Day Case (SMR04)[a]	ICD9/ICD10	1980-2020	578	Psychiatric hospital discharges and diagnostic information.
Scottish Cancer Registry (SMR06)[a]	ICD10	1996-2020	3,606	Personal, demographic, and diagnostic information on all new cases of cancer.
Scottish Birth Record (SMR11)[a]	ICD9/ICD10	1975-1992	3,246	Linked maternity, neonatal and stillbirth and infant death records from 1975, with records pertaining to mother and baby held together.
**Primary Care**				
General Practice (GP)	ReadV2	1980-2020	19,675	GP primary care attendances.
GP Out-of-Hours Data	ReadV2	2014-2022	8,533	Data on patients seen by GP Out of Hours (OOH) services across Scotland since 2014.
NHS24	Outcome Codes	2011-2022	12,326	Records of telehealth and telecare services provided through nhs24.scot website and NHS 24 111 phone service.
Accident and Emergency (A&E)	ICD10	2007-2020	15,248	Patient attendance information at Emergency Department (EDs), Minor Injuries Units (MIUs) and community hospital A&Es across NHS Scotland.
**Other Datasets**				

Routine laboratory testing	ReadV2	1997-2018	19,089	Laboratory test results from primary and secondary care provided by the Scottish Care Information (SCI) Store.
ICU Daily Data (SICSAG)	None	2019-2022	361	Scottish Intensive Care Society Audit Group (SICSAG) national database of patients admitted to adult Critical Care Units in Scotland since 1995.
ICU Episode Data (SICSAG)	None	2019-2022	361	
National Records of Scotland (NRS) Deaths Data	ICD10	2007-2022	1,659	All deaths occurring in Scotland. NRS Death Records are linked with NHS Scotland Scottish Morbidity Database.
Diabetes Registry (SCI-DC)	None	2019	1,423	The Scottish Care Information – Diabetes Collaboration (SCI-DC), established in 2002. Integrated electronic patient record for individuals with diabetes.
Pre-2009 Dispensing Data (PIS)	BNF	1989-2009	12,981	The Prescribing Information System (PIS) covers all NHS prescriptions prescribed, dispensed and reimbursed within the community setting. Pre-2009 data is poorly completed.
Post-2009 Dispensing Data (PIS)	BNF	2009-2020	21,486	
COVID-19 Vaccinations	None	2020-2022	19,128	Scottish Covid-19 vaccination data contains COVID-19 vaccination events since December 2020.
Testing (ECOSS)	None	2020-2022	16,537	Electronic Communication of Surveillance in Scotland (ECOSS) system surveillance data on COVID-19 infections reported from diagnostics and reference laboratories.
Dental (MIDAS)	SDR	1994-2019	19,870	Management Information & Dental Accounting System (MIDAS), the payment system for GDS dentists, information on NHS dentist appointments.
Scottish Drug Misuse Database	None	2001-2016	76	Scottish Drug Misuse Database (SDMD), attendances at broad range of services.

Appendix C. Secondary Care ICD-10 codes used to investigate cohort morbidities. Codes were derived from Gadd et al [43] using CALIBER code lists. ICD-10 codes are used for all records after 1994 when they replaced the use of ICD-9 codes. Codes were used to query SMR01 (General/Acute Inpatient and Day Case), SMR06 (Scottish Cancer Registry) and NRS death registration datasets.

Secondary Care ICD-10 Codes	
**Code**	**Description**
**Asthma**	
J45	Asthma
J46	Status asthmaticus
**Bowel cancer**	
C18	Malignant neoplasm of colon
**Breast cancer**	
C50	Malignant neoplasm of breast
**Chronic obstructive pulmonary disease**	
J40	Bronchitis, not specified as acute or chronic
J41	Simple and mucopurulent chronic bronchitis
J42	Unspecified chronic bronchitis
J43	Emphysema
J44	Other chronic obstructive pulmonary disease
**Dementia**	
F00	Dementia in Alzheimer's disease
F01	Vascular dementia
F03	Unspecified dementia
F051	Delirium superimposed on dementia
G30	Alzheimer's disease
**Depression**	
F32	Major depressive disorder
F33	Major depressive disorder
**Diabetes**	
E10	Type 1 diabetes mellitus
E11	Type 2 diabetes mellitus
E12	Malnutrition-related diabetes mellitus
E13	Other specified diabetes mellitus
E14	Unspecified diabetes mellitus
H360	Diabetic retinopathy
O243	Diabetes mellitus in pregnancy: Pre-existing diabetes mellitus, unspecified
N083	Glomerular disorders in diabetes mellitus
H280	Diabetic cataract
O242	Diabetes mellitus in pregnancy: Pre-existing malnutrition-related diabetes mellitus
M142	Diabetic arthropathy
G632	Diabetic polyneuropathy
O240	Diabetes mellitus in pregnancy: Pre-existing diabetes mellitus, insulin-dependent
O241	Diabetes mellitus in pregnancy: Pre-existing diabetes mellitus, non-insulin-dependent
G590	Diabetic mononeuropathy
**Hypertension**	
I10	Essential (primary) hypertension

I11	Hypertensive heart disease
I12	Hypertensive renal disease
I13	Hypertensive heart and renal disease
I15	Secondary hypertension
**Ischaemic heart disease**	
I20	Angina pectoris
I21	Acute myocardial infarction
I22	Subsequent myocardial infarction
I23	Certain current complications following acute myocardial infarction
I24	Other acute ischaemic heart diseases
I25	Chronic ischaemic heart disease
**Lung Cancer**	
C34	Malignant neoplasm of bronchus and lung
**Osteoarthritis**	
M15	Polyarthrosis
M16	Coxarthrosis [arthrosis of hip]
M17	Gonarthrosis [arthrosis of knee]
M18	Arthrosis of first carpometacarpal joint
M19	Other arthrosis
**Prostate cancer**	
C61	Malignant neoplasm of prostate
**Rheumatoid arthritis**	
M05	Seropositive rheumatoid arthritis
M06	Other rheumatoid arthritis
J99.0	Rheumatoid lung disease
**Stroke**	
I63	Cerebral infarction
I64	Stroke, not specified as haemorrhage or infarction
G46.7	Other lacunar syndromes
G46.6	Pure sensory lacunar syndrome
G46.5	Pure motor lacunar syndrome
G46.3	Brain stem stroke syndrome
G46.4	Cerebellar stroke syndrome
I69.4	Sequelae of stroke, not specified as haemorrhage or infarction
G46.8	Other vascular syndromes of brain in cerebrovascular diseases
**COVID-19**	
U071	COVID-19, virus identified

Appendix D. Secondary Care ICD-9 Codes used to investigate cohort morbidities. ICD-9 codes were used for records up to 1994 when they were replaced with ICD-10. Codes were used to query SMR01 (General/Acute Inpatient and Day Case), SMR06 (Scottish Cancer Registry) and NRS death records datasets.

**Secondary Care ICD-9 Codes**	
**Code**	**Description**
**Asthma**	
493	Asthma
**Bowel cancer**	
153	Malignant neoplasm of colon
154	Malignant neoplasm of rectum rectosigmoid junction and anus
**Breast cancer**	
174	Malignant neoplasm of female breast
**chronic obstructive pulmonary disease**	
490	Bronchitis, not specified as acute or chronic
491	Chronic bronchitis
492	Emphysema
496	Chronic airway obstruction, not elsewhere classified
**Dementia**	
290	Dementias
2912	Alcohol-induced persisting dementia
2941	Dementia in conditions classified elsewhere
331	Other cerebral degenerations
**Depression**	
2962	Major depressive disorder single episode
2963	Major depressive disorder recurrent episode
311	Depressive disorder not elsewhere classified
**Diabetes**	
250	Diabetes mellitus
249	Secondary diabetes mellitus
**Hypertension**	
401	Essential hypertension
402	Hypertensive heart disease
403	Hypertensive renal disease
404	Hypertensive heart and renal disease
405	Secondary hypertension
**Ischaemic heart disease**	
410	Acute myocardial infarction
411	Other acute and subacute forms of ischemic heart disease
412	Old myocardial infarction
413	Angina pectoris
414	Other forms of chronic ischemic heart disease
**Lung cancer**	
162	Malignant neoplasm of trachea bronchus and lung
**Osteoarthritis**	
715	Osteoarthrosis and allied disorders
**Prostate cancer**	

185	Malignant neoplasm of prostate
**Rheumatoid arthritis**	
714	Rheumatoid arthritis and other inflammatory polyarthropathies
**Stroke**	
430	Subarachnoid haemorrhage
431	Intracerebral haemorrhage
433	Occlusion and stenosis of precerebral arteries
434	Occlusion of cerebral arteries

For peer review only

Appendix E. Primary Care ReadCode v2 Codes used to investigate cohort morbidities. Codes were derived from Gadd et al [43] using CALIBER code lists.

**Primary Care ReadCode v2 Codes**	
**Code**	**Description**
**Asthma**	
14B4.	H/O: asthma
173A.	Exercise induced asthma
173c.	Occupational asthma
173d.	Work aggravated asthma
1780	Aspirin induced asthma
1O2..	Asthma confirmed
21262	Asthma resolved
212G.	Asthma resolved
H3120	Chronic asthmatic bronchitis
H33..	Asthma
H330.	Extrinsic (atopic) asthma
H3300	Extrinsic asthma without status asthmaticus
H3301	Extrinsic asthma with status asthmaticus
H330z	Extrinsic asthma NOS
H331.	Intrinsic asthma
H3310	Intrinsic asthma without status asthmaticus
H3311	Intrinsic asthma with status asthmaticus
H331z	Intrinsic asthma NOS
H332.	Mixed asthma
H333.	Acute exacerbation of asthma
H334.	Brittle asthma
H335.	Chronic asthma with fixed airflow obstruction
H33z.	Asthma unspecified
H33z0	Status asthmaticus NOS
H33z1	Asthma attack
H33z2	Late-onset asthma
H33zz	Asthma NOS
**Bowel cancer**	
B13..	Malignant neoplasm of colon
B130.	Malignant neoplasm of hepatic flexure of colon
B131.	Malignant neoplasm of transverse colon
B132.	Malignant neoplasm of descending colon
B133.	Malignant neoplasm of sigmoid colon
B134.	Malignant neoplasm of caecum
B135.	Malignant neoplasm of appendix
B136.	Malignant neoplasm of ascending colon
B137.	Malignant neoplasm of splenic flexure of colon
B139.	Hereditary nonpolyposis colon cancer
B13z.	Malignant neoplasm of colon NOS
B1z0.	Malignant neoplasm of intestinal tract, part unspecified
**Breast cancer**	
B3251	Malignant melanoma of breast

For peer review only

B3351	Malignant neoplasm of skin of chest, excluding breast
B3352	Malignant neoplasm of skin of breast
B34..	Malignant neoplasm of female breast
B3401	Malignant neoplasm of areola of female breast
B34y.	Malignant neoplasm of other site of female breast
B34yz	Malignant neoplasm of other site of female breast NOS
B34z.	Malignant neoplasm of female breast NOS
B35..	Malignant neoplasm of male breast
B58y0	Secondary malignant neoplasm of breast
B830.	Carcinoma in situ of breast
BB94.	[M]Juvenile breast carcinoma
Byu6.	[X]Malignant neoplasm of breast
ZV103	[V]Personal history of malignant neoplasm of breast
**Chronic obstructive pulmonary disease**	
14B3.	History of chronic obstructive pulmonary disease
H3...	Chronic obstructive pulmonary disease
H31..	Chronic bronchitis
H310.	Simple chronic bronchitis
H3100	Chronic catarrhal bronchitis
H310z	Simple chronic bronchitis NOS
H311.	Mucopurulent chronic bronchitis
H3110	Purulent chronic bronchitis
H3111	Fetid chronic bronchitis
H311z	Mucopurulent chronic bronchitis NOS
H312.	Obstructive chronic bronchitis
H3120	Chronic asthmatic bronchitis
H3121	Emphysematous bronchitis
H3122	Acute exacerbation of chronic obstructive airways disease
H3123	Bronchiolitis obliterans
H312z	Obstructive chronic bronchitis NOS
H313.	Mixed simple and mucopurulent chronic bronchitis
H31y.	Other chronic bronchitis
H31y1	Chronic tracheobronchitis
H31yz	Other chronic bronchitis NOS
H31z.	Chronic bronchitis NOS
H32..	Emphysema
H320.	Chronic bullous emphysema
H3200	Segmental bullous emphysema
H3201	Zonal bullous emphysema
H3202	Giant bullous emphysema
H3203	Bullous emphysema with collapse
H320z	Chronic bullous emphysema NOS
H321.	Panlobular emphysema
H322.	Centrilobular emphysema
H32y.	Other emphysema
H32y0	Acute vesicular emphysema
H32y1	Atrophic (senile) emphysema
H32y2	MacLeod's unilateral emphysema

For peer review only

H32yz	Other emphysema NOS
H32z.	Emphysema NOS
H36..	Mild chronic obstructive pulmonary disease
H37..	Moderate chronic obstructive pulmonary disease
H38..	Severe chronic obstructive pulmonary disease
H39..	Very severe chronic obstructive pulmonary disease
H3A..	End stage chronic obstructive airways disease
H3y..	Other specified chronic obstructive airways disease
H3y0.	Chronic obstruct pulmonary dis with acute lower respiratory infection
H3y1.	Chronic obstruct pulmonary dis with acute exacerbation, unspecified
H3z..	Chronic obstructive airways disease NOS
H4640	Chronic emphysema due to chemical fumes
H4641	Obliterative bronchiolitis due to chemical fumes
H5832	Eosinophilic bronchitis
Hyu30	[X]Other emphysema
Hyu31	[X]Other specified chronic obstructive pulmonary disease
**Dementia**	
1461	H/O: dementia
66h..	Dementia monitoring
6AB..	Dementia annual review
8CMZ.	Dementia care plan
9hD..	Exception reporting: dementia quality indicators
9hD0.	Excepted from dementia quality indicators: Patient unsuitable
9hD1.	Excepted from dementia quality indicators: Informed dissent
9Ou..	Dementia monitoring administration
9Ou1.	Dementia monitoring first letter
9Ou2.	Dementia monitoring second letter
9Ou3.	Dementia monitoring third letter
9Ou4.	Dementia monitoring verbal invite
9Ou5.	Dementia monitoring telephone invite
E00..	Senile and presenile organic psychotic conditions
E000.	Uncomplicated senile dementia
E001.	Presenile dementia
E0010	Uncomplicated presenile dementia
E0011	Presenile dementia with delirium
E0012	Presenile dementia with paranoia
E0013	Presenile dementia with depression
E001z	Presenile dementia NOS
E002.	Senile dementia with depressive or paranoid features
E0020	Senile dementia with paranoia
E0021	Senile dementia with depression
E002z	Senile dementia with depressive or paranoid features NOS
E003.	Senile dementia with delirium
E004.	Arteriosclerotic dementia
E0040	Uncomplicated arteriosclerotic dementia
E0041	Arteriosclerotic dementia with delirium
E0042	Arteriosclerotic dementia with paranoia

For peer review only

E0043	Arteriosclerotic dementia with depression
E004z	Arteriosclerotic dementia NOS
E00y.	Other senile and presenile organic psychoses
E00z.	Senile or presenile psychoses NOS
E041.	Dementia in conditions EC
Eu00.	[X]Dementia in Alzheimer's disease
Eu000	[X]Dementia in Alzheimer's disease with early onset
Eu001	[X]Dementia in Alzheimer's disease with late onset
Eu002	[X]Dementia in Alzheimer's dis, atypical or mixed type
Eu00z	[X]Dementia in Alzheimer's disease, unspecified
Eu01.	[X]Vascular dementia
Eu010	[X]Vascular dementia of acute onset
Eu011	[X]Multi-infarct dementia
Eu012	[X]Subcortical vascular dementia
Eu013	[X]Mixed cortical and subcortical vascular dementia
Eu01y	[X]Other vascular dementia
Eu01z	[X]Vascular dementia, unspecified
Eu02z	[X] Unspecified dementia
Eu041	[X]Delirium superimposed on dementia
F110.	Alzheimer's disease
F1100	Alzheimer's disease with early onset
F1101	Alzheimer's disease with late onset
Fyu30	[X]Other Alzheimer's disease
ZS7C5	Language disorder of dementia
**Depression**	
1465	H/O: depression
212S.	Depression resolved
8BK0.	Depression management programme
8CAa.	Patient given advice about management of depression
8HHq.	Referral for guided self-help for depression
9H90.	Depression annual review
9H91.	Depression medication review
9H92.	Depression interim review
9HA0.	On depression register
9k4..	Depression - enhanced services administration
9k40.	Depression - enhanced service completed
9kQ..	On full dose long term treatment depression - enh serv admin
9Ov..	Depression monitoring administration
9Ov0.	Depression monitoring first letter
9Ov1.	Depression monitoring second letter
9Ov2.	Depression monitoring third letter
9Ov3.	Depression monitoring verbal invite
9Ov4.	Depression monitoring telephone invite
E0013	Presenile dementia with depression
E0021	Senile dementia with depression
E0043	Arteriosclerotic dementia with depression
E112.	Single major depressive episode
E1120	Single major depressive episode, unspecified

For peer review only

E1121	Single major depressive episode, mild
E1122	Single major depressive episode, moderate
E1123	Single major depressive episode, severe, without psychosis
E1124	Single major depressive episode, severe, with psychosis
E1125	Single major depressive episode, partial or unspec remission
E1126	Single major depressive episode, in full remission
E112z	Single major depressive episode NOS
E113.	Recurrent major depressive episode
E1130	Recurrent major depressive episodes, unspecified
E1131	Recurrent major depressive episodes, mild
E1132	Recurrent major depressive episodes, moderate
E1133	Recurrent major depressive episodes, severe, no psychosis
E1134	Recurrent major depressive episodes, severe, with psychosis
E1135	Recurrent major depressive episodes,partial/unspec remission
E1136	Recurrent major depressive episodes, in full remission
E1137	Recurrent depression
E113z	Recurrent major depressive episode NOS
E118.	Seasonal affective disorder
E11y2	Atypical depressive disorder
E11z2	Masked depression
E130.	Reactive depressive psychosis
E135.	Agitated depression
E2003	Anxiety with depression
E291.	Prolonged depressive reaction
E2B..	Depressive disorder NEC
E2B1.	Chronic depression
Eu204	[X]Post-schizophrenic depression
Eu251	[X]Schizoaffective disorder, depressive type
Eu32.	[X]Depressive episode
Eu320	[X]Mild depressive episode
Eu321	[X]Moderate depressive episode
Eu322	[X]Severe depressive episode without psychotic symptoms
Eu323	[X]Severe depressive episode with psychotic symptoms
Eu324	[X]Mild depression
Eu325	[X]Major depression, mild
Eu326	[X]Major depression, moderately severe
Eu327	[X]Major depression, severe without psychotic symptoms
Eu328	[X]Major depression, severe with psychotic symptoms
Eu329	[X]Single major depr ep, severe with psych, psych in remiss
Eu32A	[X]Recurr major depr ep, severe with psych, psych in remiss
Eu32y	[X]Other depressive episodes
Eu32z	[X]Depressive episode, unspecified
Eu33.	[X]Recurrent depressive disorder
Eu330	[X]Recurrent depressive disorder, current episode mild
Eu331	[X]Recurrent depressive disorder, current episode moderate
Eu332	[X]Recurr depress disorder cur epi severe without psyc sympt
Eu333	[X]Recurrent depress disorder cur epi severe with psyc symp
Eu334	[X]Recurrent depressive disorder, currently in remission

For peer review only

Eu33y	[X]Other recurrent depressive disorders
Eu33z	[X]Recurrent depressive disorder, unspecified
Eu341	[X]Dysthymia
Eu412	[X]Mixed anxiety and depressive disorder
**Diabetes**	
C108.	Type 1 diabetes mellitus
C1080	Type 1 diabetes mellitus with renal complications
C1082	Type 1 diabetes mellitus with neurological complications
C1084	Unstable type 1 diabetes mellitus
C1085	Type 1 diabetes mellitus with ulcer
C1087	Type 1 diabetes mellitus with retinopathy
C1088	Type 1 diabetes mellitus - poor control
C1089	Type 1 diabetes mellitus maturity onset
C108A	Type 1 diabetes mellitus without complication
C108D	Type 1 diabetes mellitus with nephropathy
C108E	Type 1 diabetes mellitus with hypoglycaemic coma
C108F	Type 1 diabetes mellitus with diabetic cataract
C108H	Type 1 diabetes mellitus with arthropathy
C108J	Type 1 diabetes mellitus with neuropathic arthropathy
C109.	Type 2 diabetes mellitus
C1090	Type 2 diabetes mellitus with renal complications
C1091	Type 2 diabetes mellitus with ophthalmic complications
C1092	Type 2 diabetes mellitus with neurological complications
C1094	Type 2 diabetes mellitus with ulcer
C1095	Type 2 diabetes mellitus with gangrene
C1096	Type 2 diabetes mellitus with retinopathy
C1097	Type 2 diabetes mellitus - poor control
C109A	Type 2 diabetes mellitus with mononeuropathy
C109B	Type 2 diabetes mellitus with polyneuropathy
C109C	Type 2 diabetes mellitus with nephropathy
C109D	Type 2 diabetes mellitus with hypoglycaemic coma
C109E	Type 2 diabetes mellitus with diabetic cataract
C109F	Type 2 diabetes mellitus with peripheral angiopathy
C109G	Type 2 diabetes mellitus with arthropathy
C109H	Type 2 diabetes mellitus with neuropathic arthropathy
C109J	Insulin treated Type 2 diabetes mellitus
C109K	Hyperosmolar non-ketotic state in type 2 diabetes mellitus
C10E.	Type 1 diabetes mellitus
C10E0	Type 1 diabetes mellitus with renal complications
C10E1	Type 1 diabetes mellitus with ophthalmic complications
C10E2	Type 1 diabetes mellitus with neurological complications
C10E3	Type 1 diabetes mellitus with multiple complications
C10E4	Unstable type 1 diabetes mellitus
C10E5	Type 1 diabetes mellitus with ulcer
C10E6	Type 1 diabetes mellitus with gangrene
C10E7	Type 1 diabetes mellitus with retinopathy
C10E8	Type 1 diabetes mellitus - poor control
C10E9	Type 1 diabetes mellitus maturity onset

For peer review only

C10EA	Type 1 diabetes mellitus without complication
C10EB	Type 1 diabetes mellitus with mononeuropathy
C10EC	Type 1 diabetes mellitus with polyneuropathy
C10ED	Type 1 diabetes mellitus with nephropathy
C10EE	Type 1 diabetes mellitus with hypoglycaemic coma
C10EF	Type 1 diabetes mellitus with diabetic cataract
C10EG	Type 1 diabetes mellitus with peripheral angiopathy
C10EH	Type 1 diabetes mellitus with arthropathy
C10EJ	Type 1 diabetes mellitus with neuropathic arthropathy
C10EK	Type 1 diabetes mellitus with persistent proteinuria
C10EL	Type 1 diabetes mellitus with persistent microalbuminuria
C10EM	Type 1 diabetes mellitus with ketoacidosis
C10EN	Type 1 diabetes mellitus with ketoacidotic coma
C10EP	Type 1 diabetes mellitus with exudative maculopathy
C10EQ	Type 1 diabetes mellitus with gastroparesis
C10F.	Type 2 diabetes mellitus
C10F0	Type 2 diabetes mellitus with renal complications
C10F1	Type 2 diabetes mellitus with ophthalmic complications
C10F2	Type 2 diabetes mellitus with neurological complications
C10F3	Type 2 diabetes mellitus with multiple complications
C10F4	Type 2 diabetes mellitus with ulcer
C10F5	Type 2 diabetes mellitus with gangrene
C10F6	Type 2 diabetes mellitus with retinopathy
C10F7	Type 2 diabetes mellitus - poor control
C10F9	Type 2 diabetes mellitus without complication
C10FA	Type 2 diabetes mellitus with mononeuropathy
C10FB	Type 2 diabetes mellitus with polyneuropathy
C10FC	Type 2 diabetes mellitus with nephropathy
C10FD	Type 2 diabetes mellitus with hypoglycaemic coma
C10FE	Type 2 diabetes mellitus with diabetic cataract
C10FF	Type 2 diabetes mellitus with peripheral angiopathy
C10FG	Type 2 diabetes mellitus with arthropathy
C10FH	Type 2 diabetes mellitus with neuropathic arthropathy
C10FJ	Insulin treated Type 2 diabetes mellitus
C10FK	Hyperosmolar non-ketotic state in type 2 diabetes mellitus
C10FL	Type 2 diabetes mellitus with persistent proteinuria
C10FM	Type 2 diabetes mellitus with persistent microalbuminuria
C10FN	Type 2 diabetes mellitus with ketoacidosis
C10FP	Type 2 diabetes mellitus with ketoacidotic coma
C10FQ	Type 2 diabetes mellitus with exudative maculopathy
C10FR	Type 2 diabetes mellitus with gastroparesis
**Hypertension**	
14A2.	H/O: hypertension
21261	Hypertension resolved
212K.	Hypertension resolved
61462	Hypertension induced by oral contraceptive pill
6624	Borderline hypertension: yearly observation
6627	Good hypertension control

For peer review only

6628	Poor hypertension control
662b.	Moderate hypertension control
662c.	Hypertension six month review
662d.	Hypertension annual review
662F.	Hypertension treatment. started
662G.	Hypertensive treatment changed
662O.	On treatment for hypertension
662r.	Trial withdrawal of antihypertensive therapy
7Q01.	High cost hypertension drugs
8B26.	Antihypertensive therapy
8BL0.	Patient on maximal tolerated antihypertensive therapy
8I3N.	Hypertension treatment refused
9OI9.	Hypertension monitor deleted
F4042	Blind hypertensive eye
F4213	Hypertensive retinopathy
G2...	Hypertensive disease
G20..	Essential hypertension
G200.	Malignant essential hypertension
G201.	Benign essential hypertension
G202.	Systolic hypertension
G203.	Diastolic hypertension
G20z.	Essential hypertension NOS
G21..	Hypertensive heart disease
G210.	Malignant hypertensive heart disease
G2100	Malignant hypertensive heart disease without CCF
G2101	Malignant hypertensive heart disease with CCF
G211.	Benign hypertensive heart disease
G2110	Benign hypertensive heart disease without CCF
G2111	Benign hypertensive heart disease with CCF
G21z.	Hypertensive heart disease NOS
G21z0	Hypertensive heart disease NOS without CCF
G21z1	Hypertensive heart disease NOS with CCF
G21zz	Hypertensive heart disease NOS
G22..	Hypertensive renal disease
G220.	Malignant hypertensive renal disease
G221.	Benign hypertensive renal disease
G222.	Hypertensive renal disease with renal failure
G22z.	Hypertensive renal disease NOS
G23..	Hypertensive heart and renal disease
G230.	Malignant hypertensive heart and renal disease
G231.	Benign hypertensive heart and renal disease
G232.	Hypertensive heart&renal dis wth (congestive) heart failure
G233.	Hypertensive heart and renal disease with renal failure
G234.	Hyperten heart&renal dis+both(congestv)heart and renal fail
G23z.	Hypertensive heart and renal disease NOS
G24..	Secondary hypertension
G240.	Secondary malignant hypertension
G2400	Secondary malignant renovascular hypertension

For peer review only

G240z	Secondary malignant hypertension NOS
G241.	Secondary benign hypertension
G2410	Secondary benign renovascular hypertension
G241z	Secondary benign hypertension NOS
G244.	Hypertension secondary to endocrine disorders
G24z.	Secondary hypertension NOS
G24z0	Secondary renovascular hypertension NOS
G24z1	Hypertension secondary to drug
G24zz	Secondary hypertension NOS
G2y..	Other specified hypertensive disease
G2z..	Hypertensive disease NOS
G672.	Hypertensive encephalopathy
Gyu2.	[X]Hypertensive diseases
Gyu21	[X]Hypertension secondary to other renal disorders
L122.	Other pre-existing hypertension in preg/childbirth/puerp
L1220	Other pre-existing hypertension in preg/childb/puerp unspec
L1221	Other pre-existing hypertension in preg/childb/puerp - deliv
L1223	Other pre-exist hypertension in preg/childb/puerp-not deliv
L122z	Other pre-existing hypertension in preg/childb/puerp NOS
L127.	Pre-eclampsia or eclampsia with pre-existing hypertension
L127z	Pre-eclampsia or eclampsia + pre-existing hypertension NOS
L128.	Pre-exist hypertension compl preg childbirth and puerperium
L1280	Pre-exist hyperten heart dis compl preg childbth+puerperium
L1282	Pre-exist 2ndry hypertens comp preg childbth and puerperium
TJC7.	Adverse reaction to other antihypertensives
TJC7z	Adverse reaction to antihypertensives NOS
U60C5	[X]Oth antihyperten drug caus advers eff in therap use, NEC
**Ischaemic heart disease**	
G3...	Ischaemic heart disease
G30..	Acute myocardial infarction
G300.	Acute anterolateral infarction
G301.	Other specified anterior myocardial infarction
G301z	Anterior myocardial infarction NOS
G302.	Acute inferolateral infarction
G303.	Acute inferoposterior infarction
G304.	Posterior myocardial infarction NOS
G305.	Lateral myocardial infarction NOS
G307.	Acute subendocardial infarction
G3070	Acute non-Q wave infarction
G3071	Acute non-ST segment elevation myocardial infarction
G308.	Inferior myocardial infarction NOS
G309.	Acute Q-wave infarct
G30A.	Mural thrombosis
G30X0	Acute ST segment elevation myocardial infarction
G30yz	Other acute myocardial infarction NOS
G30z.	Acute myocardial infarction NOS
G31..	Other acute and subacute ischaemic heart disease
G310.	Postmyocardial infarction syndrome

For peer review only

G311.	Preinfarction syndrome
G3110	Myocardial infarction aborted
G3111	Unstable angina
G3112	Angina at rest
G3114	Worsening angina
G3115	Acute coronary syndrome
G31y0	Acute coronary insufficiency
G31yz	Other acute and subacute ischaemic heart disease NOS
G32..	Old myocardial infarction
G33..	Angina pectoris
G3300	Nocturnal angina
G330z	Angina decubitus NOS
G331.	Prinzmetal's angina
G332.	Coronary artery spasm
G33z.	Angina pectoris NOS
G33z3	Angina on effort
G33z4	Ischaemic chest pain
G33z6	New onset angina
G33z7	Stable angina
G33zz	Angina pectoris NOS
G340.	Coronary atherosclerosis
G3400	Single coronary vessel disease
G3401	Double coronary vessel disease
G341.	Aneurysm of heart
G3410	Ventricular cardiac aneurysm
G342.	Atherosclerotic cardiovascular disease
G343.	Ischaemic cardiomyopathy
G34z.	Other chronic ischaemic heart disease NOS
G34z0	Asymptomatic coronary heart disease
G35..	Subsequent myocardial infarction
G366.	Thrombosis of atrium, auricular appendage, and ventricle as current complications following acute myocardial infarction
G37..	Cardiac syndrome X
G38..	Postoperative myocardial infarction
G39..	Coronary microvascular disease
G3z..	Ischaemic heart disease NOS
**Lung cancer**	
B22..	Malignant neoplasm of trachea, bronchus and lung
B2211	Malignant neoplasm of hilus of lung
B222.	Malignant neoplasm of upper lobe, bronchus or lung
B2221	Malignant neoplasm of upper lobe of lung
B2231	Malignant neoplasm of middle lobe of lung
B224.	Malignant neoplasm of lower lobe, bronchus or lung
B2241	Malignant neoplasm of lower lobe of lung
B22z.	Malignant neoplasm of bronchus or lung NOS
B570.	Secondary malignant neoplasm of lung
**Osteoarthritis**	
14G2.	H/O: osteoarthritis

For peer review only

2G26.	O/E - hands - Heberden's nodes
7P204	Delivery of rehabilitation for osteoarthritis
N05..	Osteoarthritis and allied disorders
N050.	Generalised osteoarthritis - OA
N0500	Generalised osteoarthritis of unspecified site
N0501	Generalised osteoarthritis of the hand
N0502	Generalised osteoarthritis of multiple sites
N0503	Bouchard's nodes with arthropathy
N0504	Primary generalized osteoarthrosis
N0505	Secondary multiple arthrosis
N0506	Erosive osteoarthrosis
N0507	Heberden's nodes with arthropathy
N050z	Generalised osteoarthritis NOS
N051.	Localised, primary osteoarthritis
N0510	Localised, primary osteoarthritis of unspecified site
N0511	Localised, primary osteoarthritis of the shoulder region
N0512	Localised, primary osteoarthritis of the upper arm
N0513	Localised, primary osteoarthritis of the forearm
N0514	Localised, primary osteoarthritis of the hand
N0515	Localised, primary osteoarthritis of the pelvic region/thigh
N0516	Localised, primary osteoarthritis of the lower leg
N0517	Localised, primary osteoarthritis of the ankle and foot
N0518	Localised, primary osteoarthritis of other specified site
N0519	Primary coxarthrosis, bilateral
N051A	Coxarthrosis resulting from dysplasia, bilateral
N051B	Primary gonarthrosis, bilateral
N051C	Primary arthrosis of first carpometacarpal joints, bilateral
N051D	Localised, primary osteoarthritis of the wrist
N051E	Localised, primary osteoarthritis of toe
N051F	Localised, primary osteoarthritis of elbow
N051G	Osteoarthritis of spinal facet joint
N051z	Localised, primary osteoarthritis NOS
N052.	Localised, secondary osteoarthritis
N0520	Localised, secondary osteoarthritis of unspecified site
N0521	Localised, secondary osteoarthritis of the shoulder region
N0522	Localised, secondary osteoarthritis of the upper arm
N0523	Localised, secondary osteoarthritis of the forearm
N0524	Localised, secondary osteoarthritis of the hand
N0525	Localised, secondary osteoarthritis of pelvic region/thigh
N0526	Localised, secondary osteoarthritis of the lower leg
N0527	Localised, secondary osteoarthritis of the ankle and foot
N0528	Localised, secondary osteoarthritis of other specified site
N052z	Localised, secondary osteoarthritis NOS
N053.	Localised osteoarthritis, unspecified
N0530	Localised osteoarthritis, unspecified, of unspecified site
N0531	Localised osteoarthritis, unspecified, of shoulder region
N0532	Localised osteoarthritis, unspecified, of the upper arm
N0533	Localised osteoarthritis, unspecified, of the forearm

For peer review only

N0534	Localised osteoarthritis, unspecified, of the hand
N0535	Localised osteoarthritis, unspecified, pelvic region/thigh
N0536	Localised osteoarthritis, unspecified, of the lower leg
N0537	Localised osteoarthritis, unspecified, of the ankle and foot
N0538	Localised osteoarthritis, unspecified, of other spec site
N0539	Arthrosis of first carpometacarpal joint, unspecified
N053z	Localised osteoarthritis, unspecified, NOS
N054.	Oligoarticular osteoarthritis, unspecified
N0540	Oligoarticular osteoarthritis, unspec, of unspecified sites
N0541	Oligoarticular osteoarthritis, unspecified, of shoulder
N0542	Oligoarticular osteoarthritis, unspecified, of upper arm
N0544	Oligoarticular osteoarthritis, unspecified, of hand
N0545	Oligoarticular osteoarthritis, unspecified, of pelvis/thigh
N0546	Oligoarticular osteoarthritis, unspecified, of lower leg
N0547	Oligoarticular osteoarthritis, unspecified, of ankle/foot
N0548	Oligoarticular osteoarthritis, unspecified, other spec sites
N0549	Oligoarticular osteoarthritis, unspecified, multiple sites
N054z	Osteoarthritis of more than one site, unspecified, NOS
N05z.	Osteoarthritis NOS
N05z0	Osteoarthritis NOS, of unspecified site
N05z1	Osteoarthritis NOS, of shoulder region
N05z4	Osteoarthritis NOS, of the hand
N05z5	Osteoarthritis NOS, pelvic region/thigh
N05z6	Osteoarthritis NOS, of the lower leg
N05z7	Osteoarthritis NOS, of ankle and foot
N05z8	Osteoarthritis NOS, other specified site
N05z9	Osteoarthritis NOS, of shoulder
N05zA	Osteoarthritis NOS, of sternoclavicular joint
N05zB	Osteoarthritis NOS, of acromioclavicular joint
N05zC	Osteoarthritis NOS, of elbow
N05zD	Osteoarthritis NOS, of distal radio-ulnar joint
N05zE	Osteoarthritis NOS, of wrist
N05zF	Osteoarthritis NOS, of MCP joint
N05zG	Osteoarthritis NOS, of PIP joint of finger
N05zH	Osteoarthritis NOS, of DIP joint of finger
N05zJ	Osteoarthritis NOS, of hip
N05zK	Osteoarthritis NOS, of sacro-iliac joint
N05zL	Osteoarthritis NOS, of knee
N05zM	Osteoarthritis NOS, of tibio-fibular joint
N05zN	Osteoarthritis NOS, of ankle
N05zP	Osteoarthritis NOS, of subtalar joint
N05zQ	Osteoarthritis NOS, of talonavicular joint
N05zR	Osteoarthritis NOS, of other tarsal joint
N05zS	Osteoarthritis NOS, of 1st MTP joint
N05zT	Osteoarthritis NOS, of lesser MTP joint
N05zU	Osteoarthritis NOS, of IP joint of toe
N05zz	Osteoarthritis NOS
Nyu2.	[X]Arthrosis

For peer review only

Nyu20	[X]Other polyarthrosis
Nyu21	[X]Other primary coxarthrosis
Nyu22	[X]Other dysplastic coxarthrosis
Nyu24	[X]Other secondary coxarthrosis, bilateral
Nyu25	[X]Other primary gonarthrosis
Nyu27	[X]Other secondary gonarthrosis, bilateral
Nyu28	[X]Other secondary gonarthrosis
Nyu29	[X]Other primary arthrosis of first carpometacarpal joint
Nyu2D	[X]Other specified arthrosis
Nyu2E	[X]Other secondary coxarthrosis
**Prostate cancer**	
7B365	Radical prostatectomy without pelvic node excision
4M01.	Gleason prostate grade 5-7 (medium)
4M02.	Gleason prostate grade 8-10 (high)
4M00.	Gleason prostate grade 2-4 (low)
B46..	Malignant neoplasm of prostate
7B360	Radical prostatectomy - unspecified excision of pelvic nodes
ZV104	[V]Personal history of malignant neoplasm of prostate
7B367	Radical prostatectomy with pelvic lymphadenectomy
4M0..	Gleason grading of prostate cancer
7B200	Radical cystoprostatourethrectomy
7B366	Radical prostatectomy with pelvic node sampling
14270	H/O: prostate cancer
7B202	Radical cystoprostatectomy
**Rheumatoid arthritis**	
14G1.	H/O: rheumatoid arthritis
2G25.	O/E - hands - ulnar deviation
2G27.	O/E-hands-rheumatoid spindling
66H..	Rheumatoid arthrit. monitoring
7P203	Delivery of rehabilitation for rheumatoid arthritis
9mM..	Rheumatoid arthritis monitoring invitation
9mM0.	Rheumatoid arthritis monitoring invitation first letter
9mM1.	Rheumatoid arthritis monitoring invitation second letter
9mM2.	Rheumatoid arthritis monitoring invitation third letter
9mM3.	Rheumatoid arthritis monitoring verbal invitation
9mM4.	Rheumatoid arthritis monitoring telephone invitation
F3712	Polyneuropathy in rheumatoid arthritis
F3964	Myopathy due to rheumatoid arthritis
G5y8.	Rheumatoid myocarditis
G5yA.	Rheumatoid carditis
H570.	Rheumatoid lung
N005.	Adult Still's Disease
N04..	Rheumatoid arthritis and other inflammatory polyarthropathy
N040.	Rheumatoid arthritis
N0400	Rheumatoid arthritis of cervical spine
N0401	Other rheumatoid arthritis of spine
N0402	Rheumatoid arthritis of shoulder
N0403	Rheumatoid arthritis of sternoclavicular joint

For peer review only

N0404	Rheumatoid arthritis of acromioclavicular joint
N0405	Rheumatoid arthritis of elbow
N0406	Rheumatoid arthritis of distal radio-ulnar joint
N0407	Rheumatoid arthritis of wrist
N0408	Rheumatoid arthritis of MCP joint
N0409	Rheumatoid arthritis of PIP joint of finger
N040A	Rheumatoid arthritis of DIP joint of finger
N040B	Rheumatoid arthritis of hip
N040C	Rheumatoid arthritis of sacro-iliac joint
N040D	Rheumatoid arthritis of knee
N040E	Rheumatoid arthritis of tibio-fibular joint
N040F	Rheumatoid arthritis of ankle
N040G	Rheumatoid arthritis of subtalar joint
N040H	Rheumatoid arthritis of talonavicular joint
N040J	Rheumatoid arthritis of other tarsal joint
N040K	Rheumatoid arthritis of 1st MTP joint
N040L	Rheumatoid arthritis of lesser MTP joint
N040M	Rheumatoid arthritis of IP joint of toe
N040N	Rheumatoid vasculitis
N040P	Seronegative rheumatoid arthritis
N040Q	Rheumatoid bursitis
N040R	Rheumatoid nodule
N040S	Rheumatoid arthritis - multiple joint
N040T	Flare of rheumatoid arthritis
N041.	Felty's syndrome
N042.	Other rheumatoid arthropathy + visceral/systemic involvement
N0421	Rheumatoid lung disease
N0422	Rheumatoid nodule
N042z	Rheumatoid arthropathy + visceral/systemic involvement NOS
N047.	Seropositive errosive rheumatoid arthritis
N04X.	Seropositive rheumatoid arthritis
N04y0	Rheumatoid lung
N04y2	Adult-onset Still's disease
N3622	Swan-neck finger deformity
Nyu10	[X]Rheumatoid arthritis+involvement/other organs or systems
Nyu11	[X]Other seropositive rheumatoid arthritis
Nyu12	[X]Other specified rheumatoid arthritis
Nyu1G	[X]Seropositive rheumatoid arthritis unspecified
**Stroke**	
G6400	Cerebral infarction due to thrombosis of cerebral arteries
G6W..	Cereb infarct due unsp occlus/stenos precerebr arteries
Gyu63	[X]Cerebrl infarctn due/unspcf occlusn or sten/cerebrl artrs
G63y0	Cerebral infarct due to thrombosis of precerebral arteries
G683.	Sequelae of cerebral infarction
G64z3	Right sided cerebral infarction
Gyu6G	[X]Cereb infarct due unsp occlus/stenos precerebr arteries
G640.	Cerebral thrombosis
G64z.	Cerebral infarction NOS

For peer review only

G641.	Cerebral embolism
G63..	Infarction - precerebral
G64z0	Brainstem infarction
G6410	Cerebral infarction due to embolism of cerebral arteries
G64z4	Infarction of basal ganglia
G6X..	Cerebrl infarctn due/unspcf occlusn or sten/cerebrl artrs
G64z1	Wallenberg syndrome
G64..	Cerebral arterial occlusion
G63y1	Cerebral infarction due to embolism of precerebral arteries
G64z2	Left sided cerebral infarction
Gyu64	[X]Other cerebral infarction
14A7.	H/O: CVA/stroke
14AK.	H/O: Stroke in last year
1M4..	Central post-stroke pain
661M7	Stroke self-management plan agreed
661N7	Stroke self-management plan review
662e.	Stroke/CVA annual review
662M.	Stroke monitoring
662M1	Stroke 6 month review
662M2	Stroke initial post discharge review
7P242	Delivery of rehabilitation for stroke
8HHM.	Ref to multidisciplinary stroke function improvement service
8IEC.	Ref multidisciplinary stroke function improvement declined
9h2..	Exception reporting: stroke quality indicators
9h21.	Excepted from stroke quality indicators: Patient unsuitable
9h22.	Excepted from stroke quality indicators: Informed dissent
Fyu56	[X]Other lacunar syndromes
G66..	Stroke and cerebrovascular accident unspecified
G663.	Brain stem stroke syndrome
G664.	Cerebellar stroke syndrome
G665.	Pure motor lacunar syndrome
G666.	Pure sensory lacunar syndrome
G667.	Left sided CVA
G668.	Right sided CVA
G68X.	Sequelae of stroke, not specified as haemorrhage or infarction
Gyu6C	[X]Sequelae of stroke, not specified as haemorrhage or infarction
L440.	Stroke in the puerperium
ZV125	[V]Personal history of stroke
COVID-19	
1JX1.	Suspected disease caused by 2019-nCoV (novel coronavirus)
A7951	Disease caused by 2019-nCoV (novel coronavirus)
4J3R.	2019-nCoV (novel coronavirus) serology
4J3R1	2019-nCoV (novel coronavirus) detected

For peer review only

Appendix F. Summary of the top 50 most cited publications using GS data. A full list of publications can be found at https://www.ed.ac.uk/generation-scotland/what-found/publications.

First Author	Title	Year Published	Journal	Keywords	Citations
Wray	Genome-wide association analyses identify 44 risk variants and refine the genetic architecture of major depression.	2018	Nature Genetics	Cognition and Mental Health	2,243
Lee	Gene discovery and polygenic prediction from a genome-wide association study of educational attainment in 1.1 million individuals.	2018	Nature Genetics	Data	1,714
Howard	Genome-wide meta-analysis of depression identifies 102 independent variants and highlights the importance of the prefrontal brain regions.	2019	Nature Neuroscience	Cognition and Mental Health	1,483
Pardiñas	Common schizophrenia alleles are enriched in mutation-intolerant genes and in regions under strong background selection.	2018	Nature Genetics	Cognition and Mental Health	1,376
Okbay	Genome-wide association study identifies 74 loci associated with educational attainment.	2016	Nature	Cognition and Mental Health	1,292
Anttila	Analysis of shared heritability in common disorders of the brain.	2018	Science	Cognition and Mental Health	1,202
Pairo-Castineira	Genetic mechanisms of critical illness in Covid-19.	2020	Nature	COVID-19	1,162
UK10K Consortium	The UK10K project identifies rare variants in health and disease.	2015	Nature	Data	1,030
Rahbari	Timing, rates and spectra of human germline mutation.	2015	Nature Genetics	Data	859
Lazaridis	Genomic insights into the origin of farming in the ancient Near East.	2016	Nature	Data	850
Fitzgerald	Large-scale discovery of novel genetic causes of developmental disorders.	2014	Nature	Cognition and Mental Health	693
Repapi	Genome-wide association study identifies five loci associated with lung function.	2009	Nature Genetics	Respiratory Disease	646
Davies	Study of 300,486 individuals identifies 148 independent genetic loci influencing general cognitive function.	2018	Nature Communications	Cognition and Mental Health	551
Warren	Genome-wide association analysis identifies novel blood pressure loci and offers biological insights into cardiovascular risk.	2017	Nature genetics	Cardiovascular Disease	541
Roselli	Multi-Ethnic Genome-wide Association Study for Atrial Fibrillation.	2018	Nature Genetics	Cardiovascular	522
Grotzinger	Genomic structural equation modelling provides insights into the multivariate genetic architecture of complex traits.	2019	Nature Human Behaviour	Methodology	521
Liu	Biological and clinical insights from genomic analysis of plasma lipids in >300,000 individuals.	2017	Nature	Cardiovascular Disease	511

For peer review only

Wuttke	A catalogue of genetic loci associated with kidney function from analyses of a million individuals.	2019	Nature Genetics	Kidney Disease	509
Day	Genomic analyses identify hundreds of variants associated with age at menarche and support a role for puberty timing in cancer risk.	2017	Nature Genetics	Cancer	466
Kwong	Mental health before and during the COVID-19 pandemic in two longitudinal UK population cohorts.	2020	British Journal of Psychiatry	COVID-19	461
Soler Artigas	Genome-wide association and large-scale follow-up identifies 16 new loci influencing lung function.	2011	Nature Genetics	Respiratory Disease	458
Messner	Ultra-high-throughput clinical proteomics reveals classifiers of COVID-19 infection.	2020	Cell Systems	COVID-19	433
Bethlehem	Brain charts for the human lifespan.	2022	Nature	Brain Charts	416
Davies	Genetic contributions to variation in general cognitive function: a meta-analysis of genome-wide association studies in the CHARGE consortium (N=53,949).	2015	Molecular Psychiatry	Cognition and Mental Health	416
Davies	Genome-wide association study of cognitive functions and educational attainment in UK Biobank (N=112,151).	2016	Molecular Psychiatry	Cognition and Mental Health	370
Clarke	Genome-wide association study of alcohol consumption and genetic overlap with other health-related traits in UK Biobank (N=112,117).	2017	Molecular Psychiatry	Alcohol	368
Schmidt	PCSK9 genetic variants and risk of type 2 diabetes: a Mendelian randomisation study.	2016	The Lancet Diabetes & Endocrinolo gy	Diabetes	366
Day	Large-scale genomic analyses link reproductive aging to hypothalamic signalling, breast cancer susceptibility and BRCA1-mediated DNA repair.	2015	Nature Genetics	Cancer	366
Shrine	New genetic signals for lung function highlight pathways and pleiotropy, and chronic obstructive pulmonary disease associations across multiple ancestries.	2019	Nature Genetics	Cardiovascular	332
Luciano	Association analysis in over 329,000 individuals identifies 116 independent variants influencing neuroticism.	2017	Nature Genetics	Cognition and Mental Health	312
Turcot	Protein-altering variants associated with body mass index implicate pathways that control energy intake and expenditure in obesity.	2017	Nature Genetics	Lifestyle	300
Rietveld	Common genetic variants associated with cognitive performance identified using the proxy-phenotype method.	2014	PNAS 2014	Cognition and Mental Health	296

For peer review only

Surendran	Trans-ancestry meta-analyses identify rare and common variants associated with blood pressure and hypertension.	2016	Nature Genetics	Cardiovascular Disease	294
de Moor	Meta-analysis of Genome-wide Association Studies for Neuroticism, and the Polygenic Association with Major Depressive Disorder.	2015	JAMA Psychiatry	Cognition and Mental Health	293
Christopherson	Large-scale analyses of common and rare variants identify 12 new loci associated with atrial fibrillation.	2017	Nature Genetics	Cardiovascular Disease	290
Wain	Genome-wide association analyses for lung function and chronic obstructive pulmonary disease identify new loci and potential druggable targets.	2017	Nature genetics	Respiratory Disease	283
Brown	Vascular Consequences of Pre-eclampsia.	2015	Journal of Hypertension Volume	Cardiovascular Disease	283
Smith, D. J.	Genome-wide analysis of over 106,000 individuals identifies 9 neuroticism-associated loci.	2016	Molecular Psychiatry	Cognition and Mental Health	266
Graham	The power of genetic diversity in genome-wide association studies of lipids.	2021	Nature	GWAS	261
Chen	The Trans-Ancestral Genomic Architecture of Glycaemic Traits.	2021	Nature Genetics	Other	250
Tin	Target genes, variants, tissues and transcriptional pathways influencing human serum urate levels.	2019	Nature Genetics	Kidney Disease	250
Schumann	KLB is associated with alcohol drinking, and its gene product β-Klotho is necessary for FGF21 regulation of alcohol preference.	2016	PNAS	Alcohol	226
Abul-Husn	Personalized Medicine and the Power of Electronic Health Records.	2019	Cell	Ethical, Legal and Social Issues	218
Clarke	Common polygenic risk for autism spectrum disorder (ASD) is associated with cognitive ability in the general population.	2015	Molecular Psychiatry	Cognition and Mental Health	217
Wessel	Low-frequency and rare exome chip variants associate with fasting glucose and type 2 diabetes susceptibility.	2015	Nature Communications	Diabetes	215
Welsh	Cardiac Troponin T and Troponin I in the general population: contrasting genetic determinants and outcomes.	2019	Circulation	Cardiovascular	214
Graff	Genome-wide physical activity interactions in adiposity — a meta-analysis of 200,452 adults.	2017	PLoS Genetics	Obesity	200
Justice	Genome-Wide Meta-Analysis of 241,258 Adults Accounting for Smoking Behaviour Identifies Novel Loci for Obesity Traits.	2017	Nature Communications	Smoking, Obesity	200
Marioni	Molecular genetic contributions to socioeconomic status and intelligence.	2014	Intelligence	Cognition and Mental Health	196

Power	Genome-wide Association for Major Depression Through Age at Onset Stratification: Major Depressive Disorder Working Group of the Psychiatric Genomics Consortium.	2016	Biological Psychiatry	Cognition and Mental Health	193

For peer review only
